# Inflammation gene variants and susceptibility to albuminuria in the U.S. population: analysis in the Third National Health and Nutrition Examination Survey (NHANES III), 1991-1994

**DOI:** 10.1186/1471-2350-11-155

**Published:** 2010-11-05

**Authors:** Renée M Ned, Ajay Yesupriya, Giuseppina Imperatore, Diane T Smelser, Ramal Moonesinghe, Man-huei Chang, Nicole F Dowling

**Affiliations:** 1 Office of Public Health Genomics, Office of Surveillance, Epidemiology, and Laboratory Services, Centers for Disease Control and Prevention, Atlanta, GA, USA; 2 Division of Diabetes Translation, National Center for Chronic Disease Prevention and Health Promotion, Centers for Disease Control and Prevention, Atlanta, GA, USA; 3 American Society of Human Genetics Fellow, Centers for Disease Control and Prevention, Atlanta, GA, USA; 4 Office of Minority Health and Health Disparities, Office of the Director, Centers for Disease Control and Prevention, Atlanta, GA, USA

## Abstract

**Background:**

Albuminuria, a common marker of kidney damage, serves as an important predictive factor for the progression of kidney disease and for the development of cardiovascular disease. While the underlying etiology is unclear, chronic, low-grade inflammation is a suspected key factor. Genetic variants within genes involved in inflammatory processes may, therefore, contribute to the development of albuminuria.

**Methods:**

We evaluated 60 polymorphisms within 27 inflammatory response genes in participants from the second phase (1991-1994) of the Third National Health and Nutrition Examination Survey (NHANES III), a population-based and nationally representative survey of the United States. Albuminuria was evaluated as logarithm-transformed albumin-to-creatinine ratio (ACR), as ACR ≥ 30 mg/g, and as ACR above sex-specific thresholds. Multivariable linear regression and haplotype trend analyses were conducted to test for genetic associations in 5321 participants aged 20 years or older. Differences in allele and genotype distributions among non-Hispanic whites, non-Hispanic blacks, and Mexican Americans were tested in additive and codominant genetic models.

**Results:**

Variants in several genes were found to be marginally associated (uncorrected P value < 0.05) with log(ACR) in at least one race/ethnic group, but none remained significant in crude or fully-adjusted models when correcting for the false-discovery rate (FDR). In analyses of sex-specific albuminuria, *IL1B *(rs1143623) among Mexican Americans remained significantly associated with increased odds, while *IL1B *(rs1143623), *CRP *(rs1800947) and *NOS3 *(rs2070744) were significantly associated with ACR ≥ 30 mg/g in this population (additive models, FDR-P < 0.05). In contrast, no variants were found to be associated with albuminuria among non-Hispanic blacks after adjustment for multiple testing. The only variant among non-Hispanic whites significantly associated with any outcome was *TNF *rs1800750, which failed the test for Hardy-Weinberg proportions in this population. Haplotypes within *MBL2*, *CRP*, *ADRB2, IL4R*, *NOS3*, and *VDR *were significantly associated (FDR-P < 0.05) with log(ACR) or albuminuria in at least one race/ethnic group.

**Conclusions:**

Our findings suggest a small role for genetic variation within inflammation-related genes to the susceptibility to albuminuria. Additional studies are needed to further assess whether genetic variation in these, and untested, inflammation genes alter the susceptibility to kidney damage.

## Background

Persistent kidney damage is a defining criterion for chronic kidney disease, a condition that exists in at least 12% of adults in the United States using data from 1999-2004 [1,2]. The presence of kidney damage is often indicated by increased urine albumin, which is present in approximately 9-14% of the adult U.S. population [1,2]. Albuminuria serves not just as an important marker of kidney damage but also as a prognostic factor for the progression of kidney disease. In fact, kidney damage often precedes a decline in normal kidney function. Of great clinical importance, albuminuria is also a strong and independent predictor of cardiovascular disease (CVD), and of all-cause and CVD-related mortality (reviewed in [3,4]), even at levels conventionally considered within the normal range [5].

In adults, the prevalence of albuminuria rises with age and is further increased in the presence of hypertension or diabetes. In the U.S. population, the estimated prevalence of microalbuminuria (typically defined as urinary albumin-to-creatinine ratio (ACR) between 30 and 299 mg/g) is approximately 3% in adults without adverse health conditions, but is approximately 17% in hypertensive persons and approximately 29% in those with diabetes [6].

The underlying cause(s) of albuminuria are numerous; but, the mechanisms of injury that lead to loss of albumin in the kidneys are unclear. However, some data suggest that albuminuria is a consequence of widespread vascular endothelial dysfunction or of chronic, low-grade inflammation [7,8], two pathophysiologic processes that may also underlie the strong link between albuminuria and cardiovascular disease. These processes are themselves tightly linked, as inflammation has been shown to be a major determinant, and consequence, of endothelial dysfunction [9,10]. Since cytokines and other molecules that mediate inflammation can mediate vascular damage, they may play an important role in the pathogenesis of kidney damage. However, most studies that have found an association between albuminuria and various serum or plasma markers of inflammation (e.g., CRP, IL-6, TNF-α, fibrinogen, white blood cell count) have been cross-sectional [11-16]; therefore, no determination of temporality or causality could be made. Prospective studies have found that the development of increased urinary albumin excretion is preceded by elevated levels of inflammation markers: CRP in a general white population [17] and both CRP and fibrinogen in persons with type 2 diabetes [18]. Such studies lend support to the notion that inflammation may contribute to the development of kidney damage.

Accordingly, we were interested in testing genetic associations with albuminuria for genes involved in the inflammatory response. There have been few such studies to date, with the majority of analyses having tested for association specifically with diabetic nephropathy, end-stage renal disease, or with morbidity or mortality in these patient populations [19-22], but not with albuminuria or other measures of kidney damage. Therefore, the goal of this study was to assess the genetic contribution to the development of albuminuria by testing polymorphisms in 27 genes involved in the inflammatory response in DNA samples collected from participants in the Third National Health and Nutrition Examination Survey (NHANES III). This work is the first to examine the association of genetic polymorphisms with albuminuria using a population-based, nationally representative sample of the U.S. population.

## Methods

### Study population

NHANES III is a population-based and nationally representative survey of the civilian, noninstitutionalized population of the United States aged 2 months or older that was conducted from 1988-1994 by the National Center for Health Statistics (NCHS) at the Centers for Disease Control and Prevention (CDC) [23,24]. The survey relied on a multistage, complex survey design that included four self-identified race/ethnic groups: non-Hispanic white, non-Hispanic black, Mexican American, and "other." Oversampling in certain populations-- the very young, the elderly, non-Hispanic blacks, and Mexican Americans--was performed in order to produce more unbiased estimates of disease prevalence and other health indicators in these groups [25]. During the second phase of NHANES III (1991-1994), white blood cells were frozen and cell lines immortalized with Epstein-Barr virus, creating a DNA bank [25]. This DNA bank contains specimens from 7159 participants aged 12 years or older [26], whose characteristics have been described [27].

Analyses were limited to DNA samples of participants aged 20 years or older (N = 5948) since normal values for albumin excretion in children are not well established and the assessment of proteinuria in children differs from adults [28,29]. The following persons were then excluded: pregnant women (N = 111), persons in the "other" race/ethnic group (N = 276), and individuals with a self-reported history of kidney cancer (N = 7). We also excluded menstruating women (N = 233), as others have done [1,6,30], after finding elevated urine albumin excretion in this group (data not shown). The final sample size included 5321 adult participants: 2249 non-Hispanic whites, 1502 non-Hispanic blacks, and 1570 Mexican Americans.

### Selection of genes and polymorphisms

Specific genes were included in our analysis based on their involvement in inflammation as determined by information gathered from GeneCards [31], Gene Ontology [32,33], and the published literature in the case of *VDR *[34]. All available variants within these genes were included in the current study without regard to allele frequency or the amount of linkage disequilibrium between variants. The majority of the polymorphisms were chosen from a larger set that had been genotyped for an NHANES III study on allele frequencies and genotype prevalence in the U.S. population [27]. In addition, the two available variants from relevant genome-wide association studies were also included: *GCKR *variant rs1260326 in relation to plasma C-reactive protein levels [35]; and rs890945 in an unknown gene in relation to kidney function [36].

### Genotyping and quality control methods

Polymorphisms in *APOE *(N = 2), *CRP *(N = 15), *GCKR *(N = 1), and an unknown gene (N = 1) were genotyped in NHANES III by various investigators [26]. Our group genotyped all remaining variants included in this study; and details have been published [27]. Briefly, rs890945 and eight *CRP *variants (rs35500644, rs11265260, rs12093699, rs12744244, rs2027471, rs2592887, rs2794520, and rs3093075) were genotyped by a custom Illumina GoldenGate assay (San Diego, CA). *GCKR *variant rs1260326 was genotyped on a matrix-assisted laser-desorption ionization time-of-flight mass spectroscopy platform (Sequenom, San Diego, CA) [37]. All remaining polymorphisms were genotyped using TaqMan (Applied Biosystems, Foster City, CA) or MGB Eclipse (Nanogen, Bothell, WA) assays [27,38,39]. Genotyping was performed by laboratory staff that were blinded to the phenotypic data of the study participants. All genetic variants passed quality control criteria as defined by NCHS [26,27].

### Measurements and definitions

Urinary albumin (μg/mL) and urinary creatinine (mg/dL) were measured from one random, untimed spot urine collection as previously described [6]. Urinary albumin concentration was measured by solid-phase fluorescent immunoassay; and urinary creatinine concentration was measured by the modified kinetic Jaffe method. Further details of all NHANES III laboratory methods are available online [40]. Albuminuria was defined using the continuous variable urinary albumin-to-creatinine ratio (ACR) and as dichotomous variables using either a single threshold (urinary ACR ≥ 30 mg/g) or sex-specific cut-offs (urinary ACR ≥ 17 mg/g in men and ≥ 25 mg/g in women) to account for greater creatinine excretion in men than in women.

Several variables were used to characterize study participants and were included in multivariable-adjusted analyses. Age was categorized as 20-50 yrs or ≥ 50 yrs. Waist-to-hip ratio was defined by sex-specific cut-offs: 0.90 for males and 0.85 for females. Diabetes was defined as self-reported diabetes (answering "yes" to "ever been told you have sugar/diabetes"), but excluding women with a history of only gestational diabetes. Hypertension was defined as systolic blood pressure (bp) ≥ 140 mmHg, diastolic bp ≥ 90 mmHg, or if the participant was currently taking prescribed medicine for hypertension. Estimated glomerular filtration rate (eGFR) was calculated using the Modification of Diet in Renal Disease Study equation re-expressed for use with a standardized serum creatinine (S_cr_) assay [41-43]:

eGFR = 175 × (standardized S_cr_^-1.154^) × (age^-0.203^) × (0.742 if female) × (1.212 if black). NHANES III serum creatinine values were standardized as described [44].

### Statistical analysis

All analyses were performed using SAS-Callable SUDAAN 10 (Research Triangle Institute, Research Triangle Park, NC, USA) and SAS 9.2 (SAS Institute, Cary, NC, USA) to account for the NHANES III complex sampling design. Nationally representative estimates were calculated using sample weights for the NHANES III DNA bank as previously described [27]. Appropriate standard errors and confidence intervals were calculated using the Taylor series linearization approach [45,46] to correct for correlations within sampled clusters, including the possible genetic relatedness of persons sampled from the same household.

In descriptive analyses, the prevalence of albuminuria and the geometric mean of ACR were calculated by relevant demographic and clinical characteristics of the participants. Allele frequencies and tests for deviations from Hardy-Weinberg proportions were calculated as previously described [27]. The associations between the genetic variants and each outcome were assessed separately in each race/ethnic group in crude and adjusted logistic regression models. Associations were also examined in combined analyses of all participants after controlling for race/ethnicity in the logistic regression models. Urinary ACR was positively skewed and, thus, logarithm-transformed to approximate a normal distribution. The logarithm-transformed values were used in all analyses of urinary ACR as a continuous variable. Covariates were determined by significant association (P < 0.05) of the variable (i.e., age, sex, education, smoking, alcohol use, and waist-to-hip ratio) with the outcome in non-genetic models in at least one of the race/ethnic groups. Hypertension and diabetes were not considered, as these conditions are often part of the causal pathways that result in chronic kidney disease. Variables included in all multivariable models are listed in each table.

Genetic variants were tested assuming two modes of inheritance: additive (with a change in the odds ratio or beta-coefficient per copy of the minor allele) or codominant (all three genotypes assessed individually). The minor allele of each variant was defined by the prevalence in the total population using NHANES III genetic data [27,39]. Haplotype trend regression [47] was used to test for associations involving multiple variants within a single gene. The HAPLOTYPE procedure available in SAS/Genetics 9.2 was used to calculate the probability that an individual has a specific haplotype given their unphased genotype and the estimated haplotype frequencies. Haplotype terms that reflected the number and probabilities of the haplotypes were defined based on the method proposed by Zaykin, et al. [47]. Since our study included a large number of polymorphisms in *CRP*, the incremental search algorithm available in the HTSNP procedure in SAS/Genetics 9.2 was used to identify a set of markers for this gene that tagged non-rare (frequency >5%) haplotypes with a proportion of diversity explained (PDE) greater than 0.99. The haplotype analysis for *CRP *included only those markers identified with this procedure: rs2027471, rs3091244, and rs12744244. Rare haplotypes (frequency < 5%) within each gene were coded in aggregate as "other", except in cases where only one haplotype was rare. The major isoforms of *APOE *-ε2, ε3, and ε4-were determined from the rs429358 and rs7412 variants as previously described [39] and were tested assuming additive and codominant modes of inheritance. For all genes, the effect of each individual haplotype, as well as the overall association between all haplotypes and each outcome, were tested using regression models. Regression models with *CRP *variant rs35500644 often produced errors in SUDAAN due to its very low minor allele frequency. This variant was, consequently, excluded from presentation and inclusion in any haplotypes.

Prevalence odds ratios and beta-coefficients with 95% confidence intervals were estimated from both univariate and multivariable regression models. All associations (descriptive and genetic) were tested at the threshold of 0.05 using the Satterthwaite-adjusted F-statistic, a stable and preferred test statistic for the analysis of complex survey data [48]. For all genetic association analyses, P-values from the Satterthwaite-adjusted F-statistics were adjusted to control for the false discovery rate (FDR) [49,50] in each race/ethnic group.

## Results

Basic demographic characteristics and urinary albumin excretion profile of the included NHANES III participants (N = 5321) are listed in Table 1. The geometric mean of ACR differed among most measured demographic and clinical groups, including those defined by sex, age, and waist:hip ratio (P value ≤ 0.0007 for all). However, this measurement did not differ by race/ethnic group or by smoking status. The estimated total population prevalence of albuminuria in adults ≥ 20 years was 10.98 ± 0.80%, with approximately 9.8% of men and 12.2% of women having the condition as defined by sex-specific cutoff values. The prevalence of albuminuria was considerably higher in older participants, in persons with diabetes, and in persons with hypertension (all P <0.0001). The prevalence of albuminuria also differed by race/ethnic group, educational attainment, alcohol use, and waist:hip ratio. In participants with elevated levels of serum CRP, which is a common indicator of inflammation, the geometric mean of ACR and the prevalence of albuminuria were increased (P < 0.0001). Participants with moderately to severely reduced kidney function, defined as estimated glomerular filtration rate (eGFR) < 60 ml/min/1.73 m^2^, also displayed a higher prevalence of albuminuria and elevated geometric mean of ACR (P < 0.0001).

**Table 1:** Characteristics of included study participants (≥ 20 years old), NHANES III DNA bank, 1991-1994

			ACR		**Albuminuria**^**b**^	
			
Characteristic	Unweighted sample size (N)	Weighted frequency (%^a^)	Geometric mean (mg/g) ± SE	P-value	**Prevalence (%**^**a**^**) ± SD**	P-value
*Gender*						

Female	2884	49.38	6.40 ± 0.33	< 0.0001	12.15 ± 1.12	0.1548

Male	2437	50.62	4.28 ± 0.28		9.84 ± 1.10	

*Age (years)*						

20-50	3018	62.75	3.93 ± 0.20	< 0.0001	5.04 ± 0.62	< 0.0001

≥ 50	2303	37.25	8.48 ± 0.45		21.10 ± 1.41	

*Race/ethnicity*						

Non-Hispanic white	2249	82.35	5.32 ± 0.30	0.1486	10.44 ± 0.89	0.0021

Non-Hispanic black	1502	11.96	4.95 ± 0.28		14.99 ± 1.11	

Mexican-American	1570	5.69	4.50 ± 0.34		10.40 ± 0.99	

*Education*						

< high school	2016	21.57	6.73 ± 0.42	0.0001	16.55 ± 1.36	< 0.0001

Completed high school	1706	34.54	5.58 ± 0.32		12.67 ± 1.00	

> high school	1570	43.89	4.40 ± 0.30		7.02 ± 0.73	

*Smoking status*						

Non-smoker	2748	47.10	4.88 ± 0.36	0.0595	10.28 ± 0.99	0.1464

Former smoker	1294	26.54	5.86 ± 0.31		12.73 ± 1.09	

Current smoker	1279	26.36	5.26 ± 0.30		10.47 ± 1.20	

*Alcohol use*						

None	2838	46.09	6.45 ± 0.29	< 0.0001	14.42 ± 1.20	0.0001

1-3 drinks/week	1156	27.63	4.62 ± 0.38		7.76 ± 0.97	

≥ 4 drinks/week	1145	26.28	4.07 ± 0.29		7.88 ± 1.15	

*Waist:hip ratio*^c^						

Low	1565	35.89	4.57 ± 0.25	0.0007	6.29 ± 0.82	0.0001

High	3539	64.11	5.56 ± 0.28		12.94 ± 1.11	

*Diabetes*^d^						

No	4921	94.85	4.88 ± 0.23	< 0.0001	9.21 ± 0.64	< 0.0001

Yes	399	5.15	18.96 ± 3.16		44.03 ± 4.98	

*Serum CRP level (mg/dL)*						

< 1	4747	92.35	4.98 ± 0.25	< 0.0001	9.96 ± 0.63	< 0.0001

≥ 1	549	7.65	9.47 ± 0.99		23.68 ± 3.89	

*Hypertension*^e^						

No	3653	75.32	4.21 ± 0.21	< 0.0001	6.09 ± 0.59	< 0.0001

Yes	1659	24.68	10.26 ± 0.57		26.17 ± 1.95	

*eGFR (ml/min/1.73 m^2^)*						

≥ 60	4882	93.84	4.85 ± 0.24	< 0.0001	9.24 ± 0.74	< 0.0001

< 60	406	6.16	16.16 ± 1.74		37.81 ± 2.85	

ACR, albumin-to-creatinine ratio; CRP, C-reactive protein; eGFR, estimated glomerular filtration rate; NHANES III, Third National Health and Nutrition Examination Survey; SD, standard deviation; SE, standard error.

a) Weighted percentage based on NHANES survey weights. b) Defined as ACR ≥ 17 mg/g in men and ≥ 25 mg/g in women. c) Defined by sex-specific cut-offs: males = 0.90; females = 0.85. d) Defined as self-reported diabetes (answering "yes" to "ever been told you have sugar/diabetes"), but excluding women with a history of only gestational diabetes. e) Defined as systolic blood pressure (bp) ≥ 140 mmHg, diastolic bp ≥ 90 mmHg, or currently taking prescribed medicine for hypertension.

Allele frequencies of each of the included candidate gene polymorphisms are presented in Table 2. For 59 of the 60 variants (98.3%), the allele frequency varied significantly across race/ethnic groups (P < 0.05). There are several variants with substantial relative or absolute differences in allele frequency between populations (e.g., *CRP *variants rs3093058 and rs3093066).

**Table 2:** Weighted allele frequencies of polymorphisms examined in study participants, NHANES III DNA bank (1991-1994)

				Non-Hispanic whites (N = 2249)		Non-Hispanic blacks (N = 1502)		Mexican Americans (N = 1570)		
				
Gene	Variant	**Type/location**^**a**^	Alleles	**Frequency**^**b **^**(%)**	HWP P-value	Frequency (%)	HWP P-value	Frequency (%)	HWP P-value	**P-value**^**c**^
*ADRB2*	rs1042713	Arg16Gly	A/G	38.7	0.4558	49.8	0.8106	40.7	0.3089	< 0.0001

	rs1042714	Gln27Glu	G/C	41.6	0.8470	17.7	0.5914	21.7	0.8367	< 0.0001

*APOE*	rs429358	Cys130Arg	C/T	15.1	0.4694	22.1	0.4997	10.9	0.0177	0.0002

	rs7412	Arg176Cys	T/C	8.2	0.7409	10.2	0.1013	3.5	0.4721	0.0004

*CAT*	rs769214	-843	G/A	33.9	0.8337	41.0	0.3725	50.2	0.4067	< 0.0001

*CCL5*	rs2280788	-95	G/C	3.0	0.0233	0.7	0.8085	1.2	0.6867	< 0.0001

*CCR2*	rs1799864	Val64Ile	A/G	9.5	0.3895	14.7	0.0828	22.0	0.8849	< 0.0001

*CRP*	rs1205	3'UTR	A/G	34.3	0.0620	21.1	0.0210	35.2	0.3485	< 0.0001

	rs1417938	intronic	T/A	29.9	0.4841	11.8	0.9060	34.1	0.2799	< 0.0001

	rs1800947	Leu184Leu	C/G	2.9	0.5732	0.8	0.7876	2.1	0.4574	0.0004

	rs2808630	downstream of gene	G/A	28.5	0.8126	16.8	0.2494	21.8	0.4448	< 0.0001

	rs3091244	-389	A/C	6.4	0.6077	26.3	0.7876	4.8	0.3901	< 0.0001
			
			T/C	30.6		27.8		35.7		

	rs3093058	promoter	T/A	0.3	0.9146	15.9	0.8973	1.7	0.2351	< 0.0001

	rs3093066	3' UTR	A/C	0.7	0.7812	22.3	0.9172	1.5	0.5234	< 0.0001

	rs35500644	Thr35Thr	C/A	--^d^		2.0	0.5503	--		0.0003

	rs11265260	upstream of gene	G/A	6.1	0.6833	7.1	0.6120	3.9	0.8988	0.0057

	rs12093699	downstream of gene	A/G	30.5	0.4361	31.7	0.4314	34.8	0.5947	0.0286

	rs12744244	downstream of gene	A/C	20.5	0.5659	4.3	0.2859	13.2	0.3447	< 0.0001

	rs2027471	upstream of gene	A/T	34.5	0.2198	22.6	0.0701	36.9	0.3717	< 0.0001

	rs2592887	downstream of gene	A/G	40.4	0.5556	49.0	0.9909	40.6	0.6631	< 0.0001

	rs2794520	downstream of gene	A/G	33.6	0.2538	22.8	0.0354	35.5	0.7546	< 0.0001

	rs3093075	downstream of gene	A/C	6.6	0.4684	26.2	0.9300	4.7	0.9636	< 0.0001

*F2*	rs1799963	upstream of gene	A/G	1.2	0.5214	0.3	0.8969	1.1	0.6927	0.0042

*F5*	rs6025	Arg534Gln	A/G	2.6	0.6247	0.6	0.8195	0.9	0.0101	< 0.0001

*FCGR2A*	rs1801274	His166Arg; His167Arg	G/A	51.1	0.0235	53.2	0.1480	52.9	0.9579	0.1412

*FGB*	rs1800790	-462	A/G	19.2	0.9227	5.5	0.0015	15.0	0.6910	< 0.0001

*GCKR*	rs1260326	Pro446Leu	T/C	43.3	0.3779	15.4	0.0016	34.2	0.3443	< 0.0001

*IL1B*	rs1143623	-2022	C/G	27.8	0.9893	11.0	0.6368	43.9	0.5735	< 0.0001

*IL10*	rs1800871	-853; -819	T/C	24.2	0.5168	39.3	0.9181	37.8	0.0123	< 0.0001

	rs1800872	-626; -592	A/C	24.4	0.4456	38.9	0.6244	37.7	0.0145	< 0.0001

	rs1800896	-1116; -1082	G/A	46.9	0.5030	35.6	0.3431	30.5	0.5009	< 0.0001

*IL4*	rs2243248	-1098	G/T	7.5	0.4678	15.6	0.6580	12.3	0.0577	< 0.0001

	rs2243250	-588; -524; -590	T/C	16.1	0.6836	64.7	0.1552	43.2	0.2281	< 0.0001

	rs2243270	intronic	G/A	16.5	0.5225	64.1	0.0082	42.7	0.2506	< 0.0001

*IL4R*	rs1801275	Gln576Arg	G/A	21.3	0.7231	67.7	< 0.0001	28.6	0.5716	< 0.0001

	rs1805015	Ser503Pro	C/T	16.3	0.6704	36.4	0.9842	15.5	0.2848	< 0.0001

*ITGB3*	rs5918	Leu59Pro	C/T	16.6	0.8121	9.8	0.9417	9.8	0.0677	< 0.0001

*MBL2*	rs11003125	-618; -550	G/C	35.8	0.8188	13.4	0.3698	51.3	0.4876	< 0.0001

	rs1800450	Gly54Asp	A/G	14.4	0.0333	3.5	0.0388	14.4	0.4665	< 0.0001

	rs1800451	Gly57Glu	A/G	2.1	0.1373	23.3	0.0131	2.8	0.2854	< 0.0001

	rs5030737	Arg52Cys	T/C	6.8	0.5280	1.0	0.7461	2.5	0.0514	< 0.0001

	rs7096206	-289; -221	C/G	22.5	0.2505	14.9	0.6554	11.0	0.0133	< 0.0001

*NOS2A*	rs1800482	promoter	C/G	--		6.7	0.4602	0.3	< 0.0001	< 0.0001

	rs9282799	-2892; -1173	T/C	0.0	0.9579	4.5	0.5776	0.2	0.9494	< 0.0001

*NOS3*	rs1799983	Asp298Glu	T/G	32.6	0.3242	13.2	0.1706	19.4	0.1261	< 0.0001

	rs2070744	-786	C/T	39.2	0.5881	15.6	0.0190	24.2	0.0251	< 0.0001

*PON1*	rs662	Gln192Arg	G/A	32.2	0.5369	67.3	0.9505	46.6	0.1891	< 0.0001

	rs854560	Leu55Met	A/T	35.2	0.3791	18.0	0.4022	22.7	0.7799	< 0.0001

*PPARG*	rs1801282	Pro12Ala	G/C	13.5	0.9051	2.7	0.9100	12.3	0.5137	< 0.0001

*SERPINE1*	rs1799762	ins/del in promoter	4G/5G	53.0	0.6712	26.7	0.2376	33.5	0.4660	< 0.0001

*TGFB1/B9D2*	rs1800468	-800	A/G	7.8	0.8588	2.9	0.8929	5.0	0.6573	< 0.0001

	rs1800469	-509	T/C	31.6	0.7314	24.6	0.8500	44.2	0.2589	< 0.0001

	rs1800470	Pro10Leu	C/T	38.7	0.2483	45.8	0.5523	49.7	0.0675	< 0.0001

*TLR4*	rs4986790	Asp299Gly	G/A	6.6	0.2370	7.2	0.5877	2.6	0.2387	< 0.0001

*TNF*	rs1800629	-487; -308	A/G	17.1	0.4636	12.6	0.6949	6.9	0.8989	< 0.0001

	rs1800750	-555	A/G	1.5	< 0.0001	2.4	0.1562	2.7	0.2652	0.0326

	rs361525	-417; -238	A/G	5.9	0.0156	3.8	0.8423	5.9	0.2594	0.0040

*VDR*	rs2239185	intronic	C/T	47.9	0.0008	43.5	0.6865	58.6	0.7644	< 0.0001

	rs731236	Ile352Ile; TaqI	C/T	38.2	0.2444	28.7	0.5501	23.8	0.2615	< 0.0001

Unknown	rs890945	unknown	A/T	20.8	0.5772	29.6	0.2610	29.9	0.8995	< 0.0001

HWP, Hardy-Weinberg proportions; NHANES III, Third National Health and Nutrition Examination Survey.

a) Alternate designations are given, if available. b) Weighted frequencies are presented for the first listed allele. c) Chi-square test of significance of the difference in allele frequency across race/ethnic groups. d) Cannot report due to disclosure concerns (small cell sizes).

Each polymorphism was tested individually for association with log(ACR) (as a continuous variable) and with albuminuria (as dichotomous variables) in univariate and multivariable regression models stratified by race/ethnic group. Two multivariable models were assessed: adjustment for age and (where appropriate) sex, and full adjustment for a number of demographic and clinical variables. Variants with results of P < 0.05 in additive genetic models prior to adjustment for the false-discovery rate (FDR) are presented in Tables 3, 4 and 5 (crude and fully-adjusted models only). Complete results for all individual polymorphisms under additive and codominant modes of inheritance are available in Additional File 1, Tables S1-S6.

**Table 3:** Significant associations of candidate gene polymorphisms and albumin-to-creatinine ratio^a^, additive genetic model

	Crude Model			**Fully Adjusted**^**b **^**Model**		
	
Variant	β coefficient (95% CI)	P-value	FDR-adjusted P-value	β coefficient (95% CI)	P-value	FDR-adjusted P-value
***Non-Hispanic whites***						

rs1801274 (*FCGR2A*)	0.07 (-0.01-0.14)	0.0884	0.7950	0.09 (0.01-0.17)	**0.0308**	0.5610

rs1800790 (*FGB*)	-0.07 (-0.15-0.01)	0.0854	0.7950	-0.08 (-0.17--0.00)	**0.0417**	0.5610

rs11003125 (*MBL2*)	0.10 (0.03-0.17)	**0.0058**	0.1300	0.09 (0.02-0.15)	**0.0131**	0.3930

rs9282799 (*NOS2A*)	1.31 (0.58-2.04)	**0.0011**	0.0660	0.81 (0.00-1.62)	**0.0490**	0.5610

rs1800750 (*TNF*)	0.41 (0.13-0.70)	**0.0065**	0.1300	0.35 (0.09-0.60)	**0.0092**	0.3930

						

***Non-Hispanic blacks***						

rs1205 (*CRP*)	-0.11 (-0.25-0.04)	0.1441	0.7663	-0.15 (-0.29--0.00)	**0.0441**	0.5471

rs1799963 (*F2*)	-0.88 (-2.20-0.44)	0.1810	0.7663	-0.96 (-1.88--0.04)	**0.0411**	0.5471

rs1800468 (*TGFB1/B9D2*)	-0.34 (-0.65--0.03)	**0.0328**	0.7663	-0.33 (-0.70-0.04)	0.0750	0.5471

						

***Mexican Americans***						

rs1800482 (*NOS2A*)	-0.06 (-0.69-0.57)	0.8458	0.9877	-0.32 (-0.58--0.06)	**0.0176**	0.3520

rs9282799 (*NOS2A*)	0.67 (0.29-1.05)	**0.0014**	0.0840	0.58 (-0.44-1.59)	0.2513	0.9466

rs1799983 (*NOS3*)	-0.12 (-0.30-0.06)	0.1699	0.8693	-0.18 (-0.32--0.04)	**0.0145**	0.3520

rs1799762 (*SERPINE1*)	0.10 (0.01-0.19)	**0.0295**	0.5900	0.03 (-0.07-0.12)	0.5637	0.9466

CI, confidence interval; FDR, false-discovery rate.

a) Defined as log-transformed albumin-to-creatinine ratio (ACR) and analyzed as a continuous variable. b) Analyses adjusted for age, sex, alcohol consumption, educational attainment, and waist:hip ratio.

**Table 4:** Significant associations of candidate gene polymorphisms and sex-specific albuminuria^a^, additive genetic model

	Crude Model			**Fully Adjusted**^**b **^**Model**		
	
Variant	OR (95% CI)	P-value	FDR-adjusted P-value	OR (95% CI)	P-value	FDR-adjusted P-value
***Non-Hispanic whites***						

rs1800750 (*TNF*)	2.38 (1.56-3.63)	**0.0003**	**0.0180**	2.74 (1.34-5.61)	**0.0079**	0.2370

rs361525 (*TNF*)	1.59 (1.07-2.36)	**0.0235**	0.7050	1.99 (1.23-3.25)	**0.0075**	0.2370

rs731236 (*VDR*)	1.27 (1.01-1.58)	**0.0404**	0.7904	1.16 (0.95-1.42)	0.1278	0.9818

						

***Non-Hispanic blacks***						

rs2808630 (*CRP*)	1.39 (1.12-1.72)	**0.0041**	0.2460	1.30 (1.04-1.64)	**0.0232**	0.4515

rs3093058 (*CRP*)	0.67 (0.49-0.91)	**0.0134**	0.4020	0.66 (0.48-0.89)	**0.0091**	0.4515

rs7096206 (*MBL2*)	0.70 (0.50-0.99)	**0.0423**	0.5340	0.66 (0.44-1.01)	0.0530	0.5280

rs1800468 (*TGFB1/B9D2*)	0.48 (0.24-0.96)	**0.0384**	0.5340	0.44 (0.18-1.05)	0.0622	0.5280

rs1800629 (*TNF*)	0.69 (0.47-0.99)	**0.0468**	0.5340	0.79 (0.54-1.16)	0.2172	0.8145

rs731236 (*VDR*)	1.25 (0.98-1.59)	0.0736	0.6309	1.33 (1.03-1.72)	**0.0301**	0.4515

rs890945 (Chr 5q33.3)	0.78 (0.61-1.00)	0.0534	0.5340	0.71 (0.53-0.95)	**0.0236**	0.4515

						

***Mexican Americans***						

rs11265260 (*CRP*)	0.39 (0.21-0.72)	**0.0041**	0.1121	0.51 (0.29-0.92)	**0.0274**	0.3202

rs3093075 (*CRP*)	0.40 (0.22-0.75)	**0.0057**	0.1121	0.45 (0.26-0.80)	**0.0088**	0.2069

rs1143623 (*IL1B*)	1.24 (1.09-1.40)	**0.0022**	0.1121	1.46 (1.28-1.67)	**< 0.0001**	**< 0.0001**

rs1800871 (*IL10*)	1.29 (1.03-1.62)	**0.0267**	0.3151	1.22 (0.91-1.62)	0.1753	0.5084

rs1800872 (*IL10*)	1.30 (1.04-1.64)	**0.0242**	0.3151	1.22 (0.91-1.63)	0.1673	0.5084

rs2070744 (*NOS3*)	0.76 (0.54-1.08)	0.1157	0.5012	0.67 (0.47-0.95)	**0.0276**	0.3202

rs1800629 (*TNF*)	0.63 (0.38-1.04)	0.0697	0.4745	0.56 (0.36-0.86)	**0.0107**	0.2069

CI, confidence interval; FDR, false-discovery rate; OR, odds ratio.

a) Defined as urinary albumin-to-creatinine ratio (ACR) above sex-specific thresholds (≥ 17 mg/g in men and ≥ 25 mg/g in women). b) Analyses adjusted for age, alcohol consumption, educational attainment, and waist:hip ratio.

**Table 5:** Significant associations of candidate gene polymorphisms and single-threshold albuminuria^a^, additive genetic model

	Crude Model			**Fully Adjusted**^**b **^Model		
	
Variant	OR (95% CI)	P-value	FDR-adjusted P-value	OR (95% CI)	P-value	FDR-adjusted P-value
***Non-Hispanic whites***						

rs1260326 (*GCKR*)	1.19 (0.92-1.53)	0.1774	0.8886	1.29 (1.02-1.64)	**0.0361**	0.6200

rs1800750 (*TNF*)	3.20 (2.06-4.98)	**< 0.0001**	**< 0.0001**	3.60 (1.65-7.88)	**0.0025**	0.1500

rs361525 (*TNF*)	1.71 (1.18-2.47)	**0.0060**	0.1800	1.94 (1.17-3.22)	**0.0128**	0.3840

rs2239185 (*VDR*)	0.81 (0.67-0.97)	**0.0246**	0.4020	0.84 (0.70-1.02)	0.0817	0.6200

rs731236 (*VDR*)	1.31 (1.03-1.65)	**0.0268**	0.4020	1.26 (0.99-1.61)	0.0612	0.6200

						

***Non-Hispanic blacks***						

rs1205 (*CRP*)	0.75 (0.60-0.95)	**0.0195**	0.5655	0.74 (0.56-0.98)	**0.0394**	0.7760

rs3093058 (*CRP*)	0.74 (0.54-1.01)	0.0584	0.5942	0.72 (0.52-0.99)	**0.0466**	0.7760

rs1800468 (*TGFB1/B9D2*)	0.22 (0.06-0.76)	**0.0193**	0.5655	0.12 (0.01-0.99)	**0.0493**	0.7760

						

***Mexican Americans***						

rs1800947 (*CRP*)	0.37 (0.08-1.77)	0.2022	0.6802	0.08 (0.03-0.20)	**< 0.0001**	**< 0.0001**

rs1143623 (*IL1B*)	1.30 (1.06-1.60)	**0.0149**	0.4321	1.55 (1.28-1.87)	**0.0001**	**0.0029**

rs1800871 (*IL10*)	1.24 (1.02-1.51)	**0.0361**	0.5234	1.14 (0.84-1.55)	0.3759	0.9181

rs1800872 (*IL10*)	1.25 (1.03-1.52)	**0.0290**	0.5234	1.15 (0.84-1.56)	0.3630	0.9181

rs1799983 (*NOS3*)	0.65 (0.39-1.10)	0.1046	0.6067	0.60 (0.37-0.98)	**0.0423**	0.4089

rs2070744 (*NOS3*)	0.66 (0.51-0.85)	**0.0028**	0.1624	0.62 (0.48-0.80)	**0.0008**	**0.0155**

rs1800629 (*TNF*)	0.75 (0.45-1.27)	0.2729	0.6914	0.65 (0.43-0.98)	**0.0414**	0.4089

CI, confidence interval; FDR, false-discovery rate; OR, odds ratio.

a) Defined as urinary albumin-to-creatinine ratio (ACR) above ≥ 30 mg/g regardless of gender. b) Analyses adjusted for age, sex, alcohol consumption, educational attainment, and waist:hip ratio.

In additive models, no more than five variants were associated with log(ACR) in any race/ethnic group in crude or fully-adjusted analyses (uncorrected P values < 0.05; Table 3). The results of models adjusting only for age and sex were similar to the fully-adjusted models for each variant in each race/ethnicity (Additional File 1, Table S1). Only one association with log(ACR) was statistically significant in any of the race/ethnic groups after adjustment for multiple testing: *TNF *variant rs1800750 in non-Hispanic whites in age-sex adjusted models (Additional File 1, Table S1). Of note, rs1800750 failed the test for Hardy-Weinberg proportions in this population (P < 0.0001, Table 2). Association results for log(ACR) were similar when tested in codominant models, and no FDR-adjusted P value reached statistical significance (Additional File 1, Table S2).

When albuminuria was defined as a dichotomous variable using sex-specific cutoffs, variants in *TNF *and *VDR *were associated with increased odds of albuminuria in non-Hispanic whites under an additive genetic model (Table 4 and Additional File 1, Table S3). After adjustment for multiple testing, only *TNF *variant rs1800750 was statistically significant in crude (FDR-P = 0.0180) and age-adjusted (FDR-P = 0.0059) models. Among non-Hispanic blacks, variants in *CRP*, *MBL2*, *TGFB1/B9D2*, *TNF*, *VDR *and an unknown gene (rs890945) had uncorrected P values <0.05 in crude or multivariable models. However, none of these findings were statistically significant after adjustment for multiple testing. In Mexican Americans, variants in *CRP*, *IL1B*, *IL10*, *NOS3*, *PON1*, and *TNF *were marginally associated (uncorrected P value < 0.05) with the odds of sex-specific albuminuria in one or more regression models. Of these, only *IL1B *variant rs1143623 remained significantly associated after correction for multiple testing (age-adjusted: OR = 1.36, FDR-P = 0.0058; fully-adjusted OR = 1.46, FDR-P < 0.0001) (Table 4 and Additional File 1, Table S3). Several of the variants found marginally associated with albuminuria under an additive model were also marginally associated in codominant models, although none reach statistical significance after FDR adjustment (Additional File 1, Table S4).

Compared to the results with sex-specific albuminuria, findings were not remarkably different when using the single-threshold definition of albuminuria (Table 5 and Additional File 1, Table S5). In the non-Hispanic white population, only *TNF *rs1800750 remained significantly associated with albuminuria in crude and age-sex adjusted additive genetic models. In non-Hispanic blacks, fewer polymorphisms were marginally associated with single threshold albuminuria than with sex-specific albuminuria; and none remained significant after FDR adjustment. In contrast, among Mexican Americans many of the same variants as with sex-specific albuminuria had uncorrected P-values < 0.05 for urinary ACR ≥ 30 mg/g. Three variants remained significantly associated in fully-adjusted additive models after correcting for multiple testing: rs1800947 in *CRP *(FDR-P value < 0.0001), rs1143623 in *IL1B *(FDR- P value = 0.0029), and rs2070744 in *NOS3 *(FDR-P = 0.0155). In age-sex adjusted models, the *IL1B *and *NOS3 *variants were also significantly associated with this outcome (Additional File 1, Table S5). In codominant models, the only variant significantly associated with the odds of single-threshold albuminuria was *CRP *rs1800947 among Mexican Americans (fully-adjusted model only, FDR-P < 0.0001; Table 5 and Additional File 1, Table S6).

To increase the sample size for these analyses, we also tested all participants as one group and adjusted for race/ethnicity in all regression models. Findings for each outcome in both additive and codominant genetic models were largely consistent with the race-stratified analyses for non-Hispanic whites (data not shown). The only variant to remain significant after FDR adjustment was *TNF *rs1800750, which was associated with increased prevalence of albuminuria (single-threshold and sex-specific) in at least one multivariable additive genetic model. No variants remained significant in codominant models for any outcome after correction for multiple testing (data not shown).

Haplotype analyses were conducted for the thirteen genes containing at least two genotyped polymorphisms. The C-G-C-G-G haplotype of *MBL2 *in non-Hispanic whites (crude model) and the A-C-C haplotype of *CRP *in non-Hispanic blacks (fully-adjusted model) were associated with decreased ACR after FDR adjustment (Additional File 1, Table S7). Among Mexican Americans, haplotypes within *ADRB2 *(A-G), *IL4R *(C-A), and *VDR *(T-T) were associated with log(ACR) in crude genetic models (FDR-P values < 0.05). For these haplotypes, age-sex adjusted models were only marginally associated with log(ACR) (data not shown). The C-T haplotype of *NOS3 *was statistically associated with decreased odds of categorically-defined albuminuria, in fully-adjusted models among Mexican Americans (FDR-P = 0.0220 for sex-specific albuminuria and < 0.0001 for ACR ≥ 30 mg/g). Across all populations, the only significant association in age-sex (where appropriate) adjusted models is the *NOS3 *C-T haplotype in relation to single-threshold albuminuria in Mexican Americans (OR = 0.38; uncorrected P = 0.0006; FDR-P = 0.0144) (data not shown). No other individual haplotypes or global tests for haplotype effect indicated a significant association with log(ACR) or albuminuria after adjustment for multiple testing (Additional File 1, Table S7). *APOE *isoforms were assessed individually (additive model) and in combination (codominant model). Of note, the T-T isoform (also known as ε2) was marginally associated with increased odds of sex-specific albuminuria among non-Hispanic blacks after full adjustment for covariates (OR: 1.51, 95% CI: 1.03, 2.21; uncorrected P = 0.0366), but not in crude or age-adjusted genetic models (data not shown). No other isoforms of *APOE *were associated with any outcome in any race/ethnic group, nor were any global tests of *APOE *isoforms significant in additive or codominant models.

## Discussion

Inflammation is a suspected cause of kidney damage; and we were interested in testing associations of genes involved in the inflammatory response with urinary albumin excretion. We describe here the results of this work carried out with adults who participated in NHANES III, a population-based sample survey that is nationally representative of the U.S. population. We restricted analyses to albuminuria outcomes instead of chronic kidney disease, since the genes that influence renal function may differ from those contributing to renal damage and proteinuria [51-53]. Many of the included genes are involved in other cellular or physiologic pathways, such as lipid metabolism (e.g., *APOE*, *PPARG*), hemostasis (e.g., *F2*, *F5*, *FGB*), and oxidative stress/nitric oxide production (e.g., *PON1*, *CAT*, *NOS2A*, *NOS3*).

It is unknown whether polymorphisms in inflammation genes affect the normal variation in urinary albumin excretion in humans, or whether such genetic variation plays a role only in the susceptibility to renal damage once some sort of ischemic or toxic insult is acquired. Our results only minimally support a role for such polymorphisms in the establishment of normal urinary albumin excretion, since all but one individual genotype and few haplotypes were significantly associated with continuous log(ACR) after adjustment for multiple testing. We do show that increasing minor allele copy number of polymorphisms in *TNF *in non-Hispanic whites (crude and age-adjusted models) and *IL1B *in Mexican Americans (both multivariable models) were associated with albuminuria as defined by sex-specific criteria. However, *TNF *rs1800750 failed the test for Hardy-Weinberg proportions in non-Hispanic whites, so this result should be interpreted cautiously. No polymorphisms were associated with sex-specific albuminuria in codominant models in any race/ethnic group. For comparison, we also analyzed data for albuminuria defined by a single threshold for both men and women. These analyses yielded results similar to those for sex-specific albuminuria, though among Mexican Americans, genetic variants in *CRP *and *NOS3 *were also significantly associated with ACR ≥ 30 mg/g under additive models. This *CRP *variant (rs1800947) was also the only polymorphism significantly associated with single-threshold albuminuria in codominant models after correcting for multiple testing (FDR-P < 0.0001). The significance of this variant only after full multivariable adjustment may be due to instability of the regression model because of the low allele frequency of rs1800947 in Mexican Americans. *CRP *haplotypes were not associated with single threshold albuminuria in Mexican Americans, though rare *CRP *haplotypes as a group were marginally associated (uncorrected P value < 0.05) with sex-specific ACR. The C-T haplotype of *NOS3 *was found to be significantly associated with both definitions of albuminuria in Mexican Americans, strongly implicating this gene in the susceptibility to kidney damage. We found no evidence of an effect of other haplotypes on the odds of albuminuria after adjusting for multiple testing.

We calculated the power of our study to detect associations of individual polymorphisms with log(ACR) and sex-specific albuminuria within each race/ethnic group, using a two-sided α set at 0.05 and assuming a design effect of 1.2. For log(ACR), we calculated power to detect effect sizes consistent with an R^2 ^of 0.5%. In non-Hispanic whites, we have 86% power to detect changes in the log(ACR) beta coefficient of 0.41 and 0.25 at minor allele frequencies (MAFs) of 10% and 40%, respectively. In non-Hispanic blacks, our power is 70% to detect changes in the beta of 0.33 and 0.20 at the same MAFs. For Mexican-Americans, changes in the beta coefficient of 0.45 (at 10% MAF) and 0.28 (at 40% MAF) could be detected with 72% power. For sex-specific albuminuria, we calculated the study power for an odds ratio of 1.3, which is indicative of effects found both in candidate gene studies [54] and in genome-wide association studies [55]. At MAFs of 10% and 40%, the power to detect an OR = 1.3 in non-Hispanic whites is 32% and 64%, respectively. In non-Hispanic blacks, power is 18% and 36%, respectively, at these MAFs. For the Mexican American population, study power to detect an OR = 1.3 is 24% and 49% at MAFs of 10% and 40%, respectively.

Few published studies have assessed the association of inflammation-related genes with albuminuria. No association with albuminuria was found for the *TGFB1 *Pro10Leu (rs1800470) variant in non-diabetic Chinese [56]. Neither -509C > T (rs1800469) nor rs1800470 was found associated with urine albumin levels or albuminuria in hypertensive Italian men [57], though the TC and CC genotypes of the Pro10Leu variant was found associated with albuminuria and urine albumin excretion in a separate study of Italian hypertensive patients [58]. *NOS3 *-786T > C (rs2070744), but not Asp298Glu (rs1799983), was positively associated with increased ACR and higher risk of albuminuria in diabetic and nondiabetic European American family members [59]. Neither variant was associated with urine albumin excretion or albuminuria in a smaller study of hypertensive white men [60]; nor was the Asp298Glu variant associated with urinary albumin excretion in healthy Venezuelans [61]. Neither *NOS3 *variant was associated with ACR in a family-based study of Mexican Americans [62], a finding not completely supported by our study. The AA genotype of *PON1 *variant rs662 (Gln192Arg) was recently found associated with albuminuria in a large study of Japanese, but only among women [63]. Genetic variation in the *CCL5 *gene has been associated with ACR in a non-diabetic Japanese population, but the -95C > G variant (rs2280788) included in our analyses was not studied [64]. The Pro12Ala variant (rs1801282) of *PPARG *has been associated with urinary albumin excretion in a type 2 diabetic population [65]. The well studied *APOE *variants rs429358 and rs7412, from which the *APOE *ε2, ε3, and ε4 alleles (isoforms) are composed, were weakly associated with glomerular filtration rate (a measure of kidney function) in non-Hispanic whites and non-Hispanic blacks in NHANES III, but kidney damage was not assessed separately [39]. In one study that assessed progression of chronic kidney disease, *APOE *genotypes were not significantly associated with macroalbuminuria (ACR ≥ 300 μg/mg) [66]. Studies of *MBL2 *variants have been limited primarily to type 1 diabetic populations, in which there are conflicting data on the role of *MBL2 *polymorphisms in diabetic nephropathy [67,68]. To our knowledge, there have been no published genetic association studies of urinary albumin excretion or albuminuria that included variants in many of the genes in which we found polymorphisms or haplotypes significantly associated with any outcome in at least one race/ethnic group (e.g., *CRP, IL1B, TNF*, or *VDR*).

Studies such as ours are complicated by the fact that the assessment of albuminuria in any population is not straightforward. Though the gold standard for the quantitative evaluation of proteinuria is a 24-hr urine collection, this method is inconvenient and is prone to errors [28]. For the screening of adults for proteinuria, various organizations recommend measurement of an untimed (i.e., spot) urine sample using a ratio of albumin to creatinine [69]. This use of a ratio corrects for variability due to hydration, diuretics, osmotic diuresis, and concentrating defects [28,70] and has been shown to correlate well with 24-hr urine albumin excretion measurements [28,71,72]. Most often, albuminuria is defined as urinary ACR ≥ 30 mg/g for both men and women. Sex-specific criteria have been advocated in order to account for greater creatinine excretion in men than in women [30,73-75], though there are no explicit recommendations for use of such criteria [28]. Importantly, given the strong predictive value of even low-grade albuminuria for cardiovascular disease, it has been suggested that albuminuria be interpreted as a continuum rather than as threshold cut-off values, as dichotomizing albuminuria results in the loss of important clinical information [5,76]. Therefore, we analyzed urinary ACR as both a continuous variable and as dichotomous variables. We did not adjust for hypertension or diabetes in our analyses as these conditions are major risk factors for chronic kidney disease. The genetic associations examined here may have been distorted if the included polymorphisms act, at least partially, via these causal pathways to affect urine albumin excretion.

Statistical analyses were performed for individual polymorphisms under two modes of inheritance. Additive models detect both additive and dominant genetic loci effectively, but perform poorly for recessive alleles [77,78]. Codominant models have the best performance when the true inheritance pattern is unknown [77,78]. However, codominant models seemed to be less robust in situations of low MAF, such that our estimates may be less stable and our power to identify a recessive effect poor. Furthermore, odds ratios could not be estimated for some codominant models when the MAF was very low.

Our study has several limitations, some of which are specific to analyses in NHANES. First, we included one spot urine sample for the measurement of urinary ACR, as repeat tests were performed only in a small subset of NHANES III participants [1,79]. Therefore, we could not differentiate between participants with persistent albuminuria and those with transiently elevated urinary ACR. The use of the single albuminuria measurement could misclassify cases and controls and bias our results since agreement between initial and repeat urine tests was not perfect [1,28,79]. Secondly, because NHANES III is a nationally representative study, there were few participants with severe kidney damage. In fact, only a very small proportion (N = 128; 1.03 ± 0.18%) of persons in our study population displayed clinical proteinuria (ACR ≥ 250 mg/g in men or ≥ 355 mg/g in women). This may have hampered our ability to detect genetic associations with log(ACR) or albuminuria. Also, we did not perform separate analyses for diabetics or hypertensives as the resulting sample size within each race/ethnic group would have been limiting. However, there is some evidence that similar genes may contribute to ACR in both nondiabetic and diabetic individuals [80]. Furthermore, we were unable to assess population stratification in our analyses, as data from ancestry-informative markers are not yet available for NHANES III. The large sample size of NHANES III did allow for separate analyses within each of the three main race/ethnic groups, self-reported designations that have been shown to correspond with ancestry as derived from ancestry-informative markers for these populations [81-85]. These race/ethnic group-stratified analyses enabled us to account at least partially for differences in albuminuria profile, allele frequencies, and linkage disequilibrium patterns between race/ethnicities. We cannot, however, discount that there may be residual population substructure within the race/ethnic groups that may have affected our results. Therefore, caution should be exercised in interpreting our findings, especially in non-Hispanic blacks and Mexican Americans, since these populations have experienced recent genetic admixture. In addition, the lack of "replication" of marginally associated (uncorrected P-value < 0.05) or significantly associated variants across race/ethnic groups may be due to differences in the underlying linkage disequilibrium patterns of the populations, due to untested interactions, or due to lack of power. Indeed, for categorical outcomes, our power to detect a typical effect size (OR = 1.3) in each race/ethnic group was modest. The combined analyses that included all participants and adjusted for race/ethnicity did not affect our overall findings, as the statistical weighting of the race/ethnic groups produced results not materially different than those found in non-Hispanic whites alone.

A couple of limitations are not specific to NHANES. Defining albuminuria with sex-specific criteria does not alleviate all concerns regarding the measurement of urine albumin excretion. It has been proposed that additional urinary ACR thresholds that account for differences by age [86,87] and race/ethnicity [30,74] may be needed to avoid misclassification of certain demographic groups, though no specific guidelines have been developed. In addition, the use of FDR-adjusted P values to determine statistical significance may be overly conservative, as the assumption of independent tests is not met given that some polymorphisms are correlated owing to linkage disequilibrium.

The NHANES data are a unique resource for epidemiologic analyses in a large, population-based and nationally representative sample of the U.S. population. Our findings are generalizeable to the larger U.S. population and point to a few genes that may play a role in the susceptibility to albuminuria. Ideally, we could have included other variants known to be related to the progression and severity of renal disease, such as those within inflammatory cytokine *IL-6 *and polymorphisms identified in a recent genome-wide association study as significantly associated with urine albumin excretion [36]. Such variants, however, have not yet been genotyped in NHANES III. NHANES should be used for additional research into the associations between genetic variants and renal disease and to evaluate the importance of gene-environment interactions.

## Conclusions

We report the association of a small set of polymorphisms and haplotypes in genes involved in inflammation with albuminuria-related outcomes. This study is the first to examine genetic associations with such outcomes using a population-based and nationally representative sample of the U.S. population. Additional studies are needed to further assess genetic variation in these, and untested, inflammation genes and the susceptibility to kidney damage. Furthermore, NHANES can be used to facilitate the population-level assessment of new and validated polymorphisms for kidney disease susceptibility.

## Abbreviations

ACR: albumin-to-creatinine ratio; CDC: Centers for Disease Control and Prevention; CRP: C-reactive protein; CVD: cardiovascular disease; eGFR: estimated glomerular filtration rate; FDR: false discovery rate; *IL1B*: interleukin 1 beta; IL-6: interleukin 6; MAF: minor allele frequency; NCHS: National Center for Health Statistics; NHANES III: Third National Health and Nutrition Examination Survey; *NOS3*: nitric oxide synthase 3 (endothelial cell); TNF-α: tumor necrosis factor alpha.

## Competing interests

The authors declare that they have no competing interests.

## Authors' contributions

RMN led the development of the analytic plan, directed the statistical analyses, interpreted the findings, and wrote the manuscript. AY gave advice on the analytic plan, helped to develop the statistical methods, and performed the data analyses. GI, DTS, and RM helped to develop the analytic plan and consulted on the direction of the project. RM also assisted in development of the original research idea. MC provided analytic guidance and aided in development of the statistical methods. NFD (who oversees the CDC/NCI NHANES III Genomics Working Group) provided scientific leadership. All authors critically reviewed and approved the manuscript.

## Disclaimer

The findings and conclusions in this report are those of the authors and do not necessarily represent the official position of the Centers for Disease Control and Prevention.

## Pre-publication history

The pre-publication history for this paper can be accessed here:

http://www.biomedcentral.com/1471-2350/11/155/prepub

## Supplementary Material

Additional file 1 **Tables S1-S7**. pdf file containing all supplementary tables. Click here for file
