# Supplementary material for: Inflammation gene variants and susceptibility to albuminuria in the U.S. population: analysis in the Third National Health and Nutrition Examination Survey (NHANES III), 1991-1994
Source: BMC Med Genet. 2010 Nov 5;11:155. doi: 10.1186/1471-2350-11-155 (PMC2991302; doi:10.1186/1471-2350-11-155)
Supplement: Additional file 1 — Tables S1-S7. pdf file containing all supplementary tables. [file 1471-2350-11-155-S1.PDF]

Table S1. Complete results of associations of candidate gene polymorphisms and albumin-to-creatinine ratio[a], additive genetic model

Variant	Crude Model			Age-Sex Adjusted Model			Fully Adjusted[b] Model		
	β coefficient (95% CI)	P-value	FDR-adjusted P-value	β coefficient (95% CI)	P-value	FDR-adjusted P-value	β coefficient (95% CI)	P-value	FDR-adjusted P-value
Non-Hispanic whites									
rs1042713 (*ADRB2*)	0.01 (-0.07-0.10)	0.7075	0.9228	0.03 (-0.04-0.09)	0.4016	0.7981	0.03 (-0.05-0.10)	0.4261	0.8522
rs1042714 (*ADRB2*)	-0.01 (-0.08-0.05)	0.6628	0.9092	-0.02 (-0.08-0.04)	0.4306	0.7981	-0.03 (-0.10-0.04)	0.4169	0.8522
rs429358 (*APOE*)	-0.00 (-0.14-0.14)	0.9602	0.9765	0.02 (-0.11-0.14)	0.7801	0.9883	0.01 (-0.11-0.13)	0.8638	0.9972
rs7412 (*APOE*)	0.11 (-0.03-0.25)	0.1149	0.7950	0.09 (-0.03-0.21)	0.1342	0.6091	0.07 (-0.06-0.21)	0.2804	0.8522
rs769214 (*CAT*)	-0.05 (-0.17-0.08)	0.4295	0.9092	-0.05 (-0.16-0.05)	0.2958	0.7588	-0.05 (-0.17-0.07)	0.3653	0.8522
rs2280788 (*CCL5*)	0.04 (-0.16-0.24)	0.6792	0.9092	0.09 (-0.12-0.30)	0.3798	0.7981	0.07 (-0.16-0.31)	0.5219	0.8522
rs1799864 (*CCR2*)	0.01 (-0.11-0.13)	0.9019	0.9494	0.00 (-0.13-0.13)	0.9742	0.9910	0.02 (-0.14-0.18)	0.7744	0.9680
rs1205 (*CRP*)	-0.01 (-0.10-0.07)	0.7906	0.9301	-0.00 (-0.08-0.07)	0.9223	0.9910	0.00 (-0.08-0.08)	0.9957	0.9972
rs1417938 (*CRP*)	0.07 (-0.02-0.17)	0.1325	0.7950	0.07 (-0.01-0.15)	0.0980	0.6091	0.04 (-0.03-0.12)	0.2605	0.8522
rs1800947 (*CRP*)	0.08 (-0.29-0.44)	0.6667	0.9092	0.05 (-0.28-0.38)	0.7704	0.9883	0.11 (-0.23-0.44)	0.5184	0.8522
rs2808630 (*CRP*)	-0.06 (-0.16-0.04)	0.2316	0.8380	-0.05 (-0.14-0.03)	0.1915	0.7261	-0.04 (-0.13-0.05)	0.3451	0.8522
rs3091244 (*CRP*)[c]	0.06 (-0.11-0.23)<br>0.07 (-0.03-0.17)	0.3116	0.8380	0.04 (-0.11-0.20)<br>0.06 (-0.02-0.15)	0.2536	0.7485	0.05 (-0.12-0.22)<br>0.05 (-0.04-0.13)	0.4995	0.8522
rs3093058 (*CRP*)	0.45 (-0.42-1.31)	0.2977	0.8380	0.53 (-0.22-1.28)	0.1583	0.6671	0.46 (-0.35-1.27)	0.2500	0.8522
rs3093066 (*CRP*)	-0.32 (-0.93-0.29)	0.2857	0.8380	-0.26 (-0.88-0.37)	0.4017	0.7981	-0.18 (-0.78-0.43)	0.5539	0.8522
rs11265260 (*CRP*)	0.05 (-0.09-0.20)	0.4684	0.9092	0.01 (-0.14-0.15)	0.8971	0.9910	0.00 (-0.14-0.15)	0.9682	0.9972
rs12093699 (*CRP*)	0.08 (-0.05-0.21)	0.2137	0.8380	0.07 (-0.04-0.19)	0.1969	0.7261	0.06 (-0.05-0.17)	0.2553	0.8522
rs12744244 (*CRP*)	0.09 (-0.05-0.23)	0.1929	0.8267	0.07 (-0.05-0.19)	0.2224	0.7485	0.06 (-0.05-0.18)	0.2697	0.8522
rs2027471 (*CRP*)	-0.02 (-0.11-0.06)	0.5957	0.9092	-0.01 (-0.09-0.06)	0.7122	0.9883	-0.01 (-0.08-0.07)	0.8746	0.9972
rs2592887 (*CRP*)	-0.02 (-0.11-0.07)	0.6617	0.9092	-0.02 (-0.11-0.07)	0.6481	0.9883	-0.01 (-0.11-0.08)	0.7432	0.9680
rs2794520 (*CRP*)	-0.01 (-0.10-0.09)	0.8829	0.9460	0.00 (-0.08-0.08)	0.9514	0.9910	0.01 (-0.08-0.09)	0.8983	0.9972
rs3093075 (*CRP*)	0.04 (-0.12-0.19)	0.6153	0.9092	0.00 (-0.15-0.15)	0.9630	0.9910	0.01 (-0.15-0.18)	0.8977	0.9972
rs1799963 (*F2*)	0.29 (-0.14-0.72)	0.1777	0.8267	0.17 (-0.28-0.61)	0.4427	0.7981	0.22 (-0.25-0.69)	0.3499	0.8522
rs6025 (*F5*)	0.08 (-0.09-0.26)	0.3331	0.8380	0.02 (-0.17-0.22)	0.8208	0.9883	0.01 (-0.20-0.21)	0.9506	0.9972
rs1801274 (*FCGR2A*)	0.07 (-0.01-0.14)	0.0884	0.7950	0.08 (0.01-0.15)	**0.0337**	0.5517	0.09 (0.01-0.17)	**0.0308**	0.5610
rs1800790 (*FGB*)	-0.07 (-0.15-0.01)	0.0854	0.7950	-0.06 (-0.13-0.01)	0.0744	0.6091	-0.08 (-0.17--0.00)	**0.0417**	0.5610
rs1260326 (*GCKR*)	0.02 (-0.08-0.12)	0.6563	0.9092	0.05 (-0.04-0.13)	0.2691	0.7485	0.05 (-0.03-0.13)	0.2160	0.8522
rs1143623 (*IL1B*)	-0.02 (-0.14-0.10)	0.7659	0.9242	-0.01 (-0.12-0.11)	0.9132	0.9910	-0.00 (-0.10-0.10)	0.9631	0.9972
rs1800871 (*IL10*)	0.01 (-0.08-0.11)	0.7623	0.9242	0.02 (-0.07-0.11)	0.6729	0.9883	0.03 (-0.06-0.12)	0.4534	0.8522
rs1800872 (*IL10*)	0.00 (-0.09-0.10)	0.9230	0.9548	0.01 (-0.09-0.11)	0.7934	0.9883	0.03 (-0.06-0.12)	0.5089	0.8522
rs1800896 (*IL10*)	-0.02 (-0.08-0.05)	0.5991	0.9092	-0.02 (-0.09-0.04)	0.4622	0.8021	-0.02 (-0.09-0.04)	0.4971	0.8522
rs2243248 (*IL4*)	0.03 (-0.13-0.18)	0.7330	0.9242	0.11 (-0.02-0.24)	0.0840	0.6091	0.10 (-0.06-0.27)	0.1989	0.8522
rs2243250 (*IL4*)	-0.07 (-0.15-0.02)	0.1204	0.7950	-0.08 (-0.17-0.01)	0.0913	0.6091	-0.08 (-0.17-0.01)	0.0717	0.6146
rs2243270 (*IL4*)	-0.07 (-0.17-0.03)	0.1747	0.8267	-0.08 (-0.18-0.02)	0.1168	0.6091	-0.08 (-0.18-0.02)	0.1285	0.7710
rs1801275 (*IL4R*)	-0.03 (-0.14-0.08)	0.5618	0.9092	-0.01 (-0.12-0.10)	0.8494	0.9910	0.00 (-0.12-0.12)	0.9617	0.9972
rs1805015 (*IL4R*)	-0.01 (-0.12-0.10)	0.8740	0.9460	-0.00 (-0.11-0.11)	1.0000	1.0000	0.01 (-0.10-0.12)	0.8333	0.9972
rs5918 (*ITGB3*)	0.01 (-0.08-0.09)	0.8252	0.9460	0.01 (-0.08-0.11)	0.7491	0.9883	0.01 (-0.07-0.10)	0.7420	0.9680
rs11003125 (*MBL2*)	0.10 (0.03-0.17)	**0.0058**	0.1300	0.09 (0.02-0.15)	**0.0091**	0.2685	0.09 (0.02-0.15)	**0.0131**	0.3930
rs1800450 (*MBL2*)	0.06 (-0.07-0.19)	0.3522	0.8453	0.03 (-0.09-0.16)	0.5683	0.9314	0.03 (-0.10-0.15)	0.6423	0.9176
rs1800451 (*MBL2*)	-0.02 (-0.27-0.23)	0.8679	0.9460	-0.05 (-0.30-0.20)	0.6671	0.9883	-0.04 (-0.29-0.21)	0.7416	0.9680
rs5030737 (*MBL2*)	0.21 (-0.04-0.46)	0.0964	0.7950	0.21 (-0.02-0.45)	0.0737	0.6091	0.22 (-0.01-0.44)	0.0561	0.5610
rs7096206 (*MBL2*)	-0.02 (-0.12-0.08)	0.6772	0.9092	-0.03 (-0.11-0.06)	0.5460	0.9204	-0.02 (-0.10-0.07)	0.6789	0.9473
rs1800482 (*NOS2A*)	0.43 (-0.97-1.84)	0.5304	0.9092	0.15 (-1.15-1.46)	0.8103	0.9883	0.18 (-1.04-1.39)	0.7686	0.9680
rs9282799 (*NOS2A*)	1.31 (0.58-2.04)	**0.0011**	0.0660	0.89 (0.06-1.72)	**0.0374**	0.5517	0.81 (0.00-1.62)	**0.0490**	0.5610
rs1799983 (*NOS3*)	0.02 (-0.10-0.13)	0.7702	0.9242	0.03 (-0.06-0.13)	0.4464	0.7981	0.04 (-0.05-0.12)	0.3711	0.8522
rs2070744 (*NOS3*)	-0.03 (-0.13-0.06)	0.4977	0.9092	-0.02 (-0.12-0.08)	0.6373	0.9883	-0.03 (-0.13-0.08)	0.5924	0.8735
rs662 (*PON1*)	-0.06 (-0.16-0.04)	0.2409	0.8380	-0.04 (-0.13-0.06)	0.4212	0.7981	-0.02 (-0.11-0.06)	0.5969	0.8735
rs854560 (*PON1*)	0.04 (-0.05-0.13)	0.3352	0.8380	0.04 (-0.03-0.11)	0.2698	0.7485	0.03 (-0.04-0.10)	0.3812	0.8522
rs1801282 (*PPARG*)	0.08 (-0.08-0.24)	0.3098	0.8380	0.09 (-0.03-0.22)	0.1327	0.6091	0.12 (-0.02-0.26)	0.1006	0.6707
rs1799762 (*SERPINE1*)	0.03 (-0.07-0.13)	0.5232	0.9092	0.04 (-0.06-0.14)	0.4053	0.7981	0.04 (-0.06-0.15)	0.3962	0.8522
rs1800468 (*TGFB1/B9D2*)	0.06 (-0.11-0.23)	0.4796	0.9092	0.08 (-0.07-0.24)	0.2791	0.7485	0.08 (-0.07-0.23)	0.2956	0.8522
rs1800469 (*TGFB1/B9D2*)	0.00 (-0.12-0.12)	0.9881	0.9881	-0.00 (-0.11-0.10)	0.9624	0.9910	0.00 (-0.12-0.12)	0.9972	0.9972
rs1800470 (*TGFB1*)	0.03 (-0.10-0.15)	0.6350	0.9092	0.02 (-0.09-0.14)	0.7162	0.9883	0.04 (-0.08-0.15)	0.5443	0.8522
rs4986790 (*TLR4*)	0.07 (-0.12-0.26)	0.4662	0.9092	0.07 (-0.10-0.24)	0.4220	0.7981	0.06 (-0.11-0.24)	0.4642	0.8522
rs1800629 (*TNF*)	-0.07 (-0.20-0.06)	0.2843	0.8380	-0.07 (-0.19-0.05)	0.2483	0.7485	-0.07 (-0.20-0.06)	0.2791	0.8522
rs1800750 (*TNF*)	0.41 (0.13-0.70)	**0.0065**	0.1300	0.46 (0.21-0.71)	**0.0008**	**0.0472**	0.35 (0.09-0.60)	**0.0092**	0.3930
rs361525 (*TNF*)	0.13 (-0.06-0.31)	0.1839	0.8267	0.14 (-0.04-0.32)	0.1281	0.6091	0.14 (-0.03-0.31)	0.0936	0.6707
rs2239185 (*VDR*)	-0.01 (-0.08-0.07)	0.8539	0.9460	0.01 (-0.08-0.09)	0.8717	0.9910	0.02 (-0.05-0.10)	0.5087	0.8522
rs731236 (*VDR*)	0.03 (-0.06-0.12)	0.5374	0.9092	0.01 (-0.07-0.10)	0.7838	0.9883	-0.01 (-0.09-0.08)	0.8886	0.9972
rs890945 (Chr 5q33.3)	0.02 (-0.09-0.14)	0.6819	0.9092	0.05 (-0.06-0.15)	0.3590	0.7981	0.03 (-0.07-0.13)	0.5472	0.8522

Variant	Crude Model			Age-Sex Adjusted Model			Fully Adjusted[b] Model		
	β coefficient (95% CI)	P-value	FDR-adjusted P-value	β coefficient (95% CI)	P-value	FDR-adjusted P-value	β coefficient (95% CI)	P-value	FDR-adjusted P-value
Non-Hispanic blacks									
rs1042713 (*ADRB2*)	0.00 (-0.15-0.15)	0.9960	0.9960	0.02 (-0.12-0.17)	0.7208	0.9795	0.03 (-0.11-0.16)	0.6889	0.9024
rs1042714 (*ADRB2*)	-0.02 (-0.24-0.19)	0.8302	0.9960	-0.06 (-0.27-0.15)	0.5631	0.9795	-0.07 (-0.26-0.12)	0.4448	0.8848
rs429358 (*APOE*)	0.02 (-0.15-0.19)	0.7923	0.9960	0.07 (-0.07-0.20)	0.3290	0.8440	0.05 (-0.07-0.17)	0.4152	0.8848
rs7412 (*APOE*)	0.12 (-0.08-0.32)	0.2299	0.7663	0.08 (-0.06-0.22)	0.2589	0.8108	0.10 (-0.06-0.26)	0.2016	0.6657
rs769214 (*CAT*)	-0.00 (-0.12-0.11)	0.9471	0.9960	-0.01 (-0.12-0.10)	0.8292	0.9795	-0.01 (-0.12-0.09)	0.7809	0.9024
rs2280788 (*CCL5*)	-0.31 (-0.82-0.21)	0.2290	0.7663	-0.09 (-0.60-0.43)	0.7266	0.9795	-0.06 (-0.62-0.51)	0.8339	0.9024
rs1799864 (*CCR2*)	-0.04 (-0.23-0.14)	0.6185	0.9960	-0.03 (-0.20-0.14)	0.7136	0.9795	-0.04 (-0.20-0.12)	0.6210	0.9024
rs1205 (*CRP*)	-0.11 (-0.25-0.04)	0.1441	0.7663	-0.11 (-0.24-0.02)	0.0815	0.6601	-0.15 (-0.29--0.00)	**0.0441**	0.5471
rs1417938 (*CRP*)	-0.03 (-0.28-0.22)	0.7864	0.9960	0.01 (-0.27-0.28)	0.9587	0.9866	0.07 (-0.19-0.33)	0.5870	0.9024
rs1800947 (*CRP*)	-0.44 (-1.52-0.64)	0.4109	0.8966	-0.60 (-1.50-0.30)	0.1830	0.7972	-0.55 (-1.44-0.34)	0.2124	0.6657
rs2808630 (*CRP*)	0.12 (-0.07-0.30)	0.2009	0.7663	0.07 (-0.08-0.22)	0.3228	0.8440	0.04 (-0.10-0.19)	0.5332	0.8848
rs3091244 (*CRP*)[c]	0.04 (-0.12-0.19)<br>-0.10 (-0.27-0.07)	0.3307	0.8966	0.05 (-0.06-0.16)<br>-0.06 (-0.23-0.10)	0.3933	0.9168	0.09 (-0.03-0.20)<br>-0.01 (-0.18-0.15)	0.4052	0.8848
rs3093058 (*CRP*)	-0.17 (-0.35-0.02)	0.0833	0.7663	-0.14 (-0.31-0.04)	0.1200	0.7080	-0.14 (-0.31-0.03)	0.1003	0.5471
rs3093066 (*CRP*)	0.10 (-0.07-0.27)	0.2249	0.7663	0.12 (-0.02-0.26)	0.0912	0.6601	0.13 (-0.02-0.27)	0.0776	0.5471
rs11265260 (*CRP*)	-0.03 (-0.28-0.23)	0.8328	0.9960	-0.02 (-0.27-0.22)	0.8574	0.9795	0.03 (-0.24-0.29)	0.8356	0.9024
rs12093699 (*CRP*)	0.09 (-0.06-0.23)	0.2238	0.7663	0.09 (-0.05-0.23)	0.2054	0.7972	0.08 (-0.06-0.22)	0.2441	0.6657
rs12744244 (*CRP*)	-0.01 (-0.39-0.38)	0.9762	0.9960	0.04 (-0.35-0.43)	0.8321	0.9795	0.07 (-0.32-0.46)	0.7226	0.9024
rs2027471 (*CRP*)	-0.10 (-0.24-0.04)	0.1634	0.7663	-0.10 (-0.23-0.02)	0.1007	0.6601	-0.14 (-0.28-0.00)	0.0535	0.5471
rs2592887 (*CRP*)	0.07 (-0.11-0.26)	0.4184	0.8966	0.06 (-0.09-0.21)	0.4235	0.9254	0.04 (-0.12-0.19)	0.6345	0.9024
rs2794520 (*CRP*)	-0.07 (-0.21-0.06)	0.2858	0.8574	-0.07 (-0.20-0.06)	0.2611	0.8108	-0.10 (-0.24-0.04)	0.1527	0.6371
rs3093075 (*CRP*)	0.07 (-0.09-0.23)	0.3956	0.8966	0.07 (-0.06-0.20)	0.2919	0.8440	0.09 (-0.04-0.23)	0.1805	0.6371
rs1799963 (*F2*)	-0.88 (-2.20-0.44)	0.1810	0.7663	-1.04 (-1.90--0.18)	**0.0205**	0.6601	-0.96 (-1.88--0.04)	**0.0411**	0.5471
rs6025 (*F5*)	0.07 (-0.43-0.56)	0.7817	0.9960	0.23 (-0.27-0.73)	0.3442	0.8462	0.18 (-0.43-0.80)	0.5440	0.8848
rs1801274 (*FCGR2A*)	0.09 (-0.08-0.25)	0.2781	0.8574	0.12 (-0.04-0.28)	0.1469	0.7879	0.11 (-0.05-0.27)	0.1669	0.6371
rs1800790 (*FGB*)	-0.02 (-0.28-0.24)	0.8948	0.9960	0.01 (-0.25-0.27)	0.9377	0.9866	0.10 (-0.21-0.41)	0.5130	0.8848
rs1260326 (*GCKR*)	0.02 (-0.15-0.20)	0.7743	0.9960	-0.02 (-0.19-0.14)	0.7647	0.9795	-0.02 (-0.21-0.16)	0.7834	0.9024
rs1143623 (*IL1B*)	-0.04 (-0.28-0.21)	0.7574	0.9960	0.00 (-0.21-0.21)	1.0000	1.0000	0.05 (-0.19-0.28)	0.6924	0.9024
rs1800871 (*IL10*)	-0.04 (-0.17-0.09)	0.5168	0.9396	-0.04 (-0.16-0.08)	0.5192	0.9795	-0.05 (-0.17-0.08)	0.4474	0.8848
rs1800872 (*IL10*)	-0.03 (-0.16-0.09)	0.5800	0.9667	-0.03 (-0.15-0.09)	0.5725	0.9795	-0.04 (-0.16-0.08)	0.5162	0.8848
rs1800896 (*IL10*)	-0.02 (-0.14-0.10)	0.7622	0.9960	-0.03 (-0.15-0.09)	0.5730	0.9795	-0.03 (-0.16-0.11)	0.6581	0.9024
rs2243248 (*IL4*)	-0.01 (-0.19-0.18)	0.9534	0.9960	0.01 (-0.12-0.14)	0.8633	0.9795	0.01 (-0.13-0.15)	0.9024	0.9024
rs2243250 (*IL4*)	-0.01 (-0.21-0.19)	0.8847	0.9960	0.00 (-0.18-0.19)	0.9675	0.9866	0.02 (-0.15-0.19)	0.8261	0.9024
rs2243270 (*IL4*)	-0.07 (-0.29-0.16)	0.5542	0.9501	-0.02 (-0.24-0.19)	0.8097	0.9795	-0.02 (-0.24-0.20)	0.8507	0.9024
rs1801275 (*IL4R*)	-0.03 (-0.11-0.06)	0.5027	0.9396	-0.05 (-0.14-0.05)	0.3127	0.8440	-0.08 (-0.21-0.06)	0.2434	0.6657
rs1805015 (*IL4R*)	-0.04 (-0.15-0.07)	0.4859	0.9396	-0.01 (-0.13-0.11)	0.8564	0.9795	-0.04 (-0.16-0.08)	0.4490	0.8848
rs5918 (*ITGB3*)	0.14 (-0.05-0.33)	0.1410	0.7663	0.07 (-0.11-0.26)	0.4040	0.9168	0.15 (-0.05-0.34)	0.1297	0.6018
rs11003125 (*MBL2*)	-0.14 (-0.37-0.09)	0.2114	0.7663	-0.12 (-0.32-0.09)	0.2540	0.8108	-0.12 (-0.37-0.12)	0.3079	0.8032
rs1800450 (*MBL2*)	0.28 (-0.16-0.72)	0.2064	0.7663	0.30 (-0.05-0.65)	0.0927	0.6601	0.30 (-0.10-0.70)	0.1304	0.6018
rs1800451 (*MBL2*)	-0.00 (-0.17-0.16)	0.9636	0.9960	-0.01 (-0.16-0.15)	0.9456	0.9866	-0.02 (-0.18-0.14)	0.7857	0.9024
rs5030737 (*MBL2*)	-0.24 (-0.74-0.27)	0.3429	0.8966	-0.12 (-0.74-0.50)	0.6905	0.9795	-0.25 (-1.03-0.52)	0.5072	0.8848
rs7096206 (*MBL2*)	-0.10 (-0.26-0.06)	0.2137	0.7663	-0.11 (-0.23-0.01)	0.0789	0.6601	-0.12 (-0.24-0.00)	0.0578	0.5471
rs1800482 (*NOS2A*)	0.16 (-0.01-0.33)	0.0648	0.7663	0.14 (-0.02-0.30)	0.0846	0.6601	0.16 (-0.00-0.33)	0.0541	0.5471
rs9282799 (*NOS2A*)	-0.12 (-0.46-0.22)	0.4829	0.9396	0.03 (-0.32-0.38)	0.8405	0.9795	0.04 (-0.31-0.38)	0.8333	0.9024
rs1799983 (*NOS3*)	-0.00 (-0.20-0.20)	0.9823	0.9960	-0.02 (-0.19-0.14)	0.7704	0.9795	-0.05 (-0.21-0.11)	0.5443	0.8848
rs2070744 (*NOS3*)	0.02 (-0.20-0.25)	0.8286	0.9960	0.01 (-0.16-0.19)	0.8840	0.9841	-0.02 (-0.18-0.13)	0.7774	0.9024
rs662 (*PON1*)	-0.05 (-0.18-0.09)	0.4642	0.9396	-0.01 (-0.15-0.13)	0.8581	0.9795	-0.04 (-0.17-0.09)	0.5456	0.8848
rs854560 (*PON1*)	0.00 (-0.20-0.20)	0.9664	0.9960	-0.05 (-0.22-0.12)	0.5535	0.9795	-0.10 (-0.27-0.07)	0.2394	0.6657
rs1801282 (*PPARG*)	0.16 (-0.21-0.53)	0.3778	0.8966	0.23 (-0.11-0.58)	0.1776	0.7972	0.24 (-0.11-0.59)	0.1761	0.6371
rs1799762 (*SERPINE1*)	0.02 (-0.12-0.16)	0.7405	0.9960	0.02 (-0.12-0.15)	0.8023	0.9795	0.01 (-0.13-0.15)	0.9000	0.9024
rs1800468 (*TGFB1/B9D2*)	-0.34 (-0.65--0.03)	**0.0328**	0.7663	-0.31 (-0.64-0.02)	0.0666	0.6601	-0.33 (-0.70-0.04)	0.0750	0.5471
rs1800469 (*TGFB1/B9D2*)	0.11 (-0.04-0.26)	0.1479	0.7663	0.10 (-0.06-0.26)	0.2162	0.7972	0.14 (-0.02-0.30)	0.0904	0.5471
rs1800470 (*TGFB1*)	0.09 (-0.05-0.23)	0.2116	0.7663	0.09 (-0.05-0.23)	0.2096	0.7972	0.13 (-0.02-0.28)	0.0960	0.5471
rs4986790 (*TLR4*)	0.02 (-0.30-0.33)	0.9212	0.9960	0.03 (-0.25-0.31)	0.8080	0.9795	0.02 (-0.27-0.30)	0.8937	0.9024
rs1800629 (*TNF*)	-0.09 (-0.30-0.12)	0.4075	0.8966	-0.06 (-0.25-0.13)	0.5179	0.9795	-0.01 (-0.20-0.17)	0.8775	0.9024
rs1800750 (*TNF*)	-0.01 (-0.33-0.31)	0.9545	0.9960	0.07 (-0.25-0.38)	0.6754	0.9795	0.04 (-0.31-0.39)	0.8117	0.9024
rs361525 (*TNF*)	-0.10 (-0.36-0.15)	0.4081	0.8966	-0.06 (-0.34-0.22)	0.6460	0.9795	-0.09 (-0.37-0.19)	0.4991	0.8848
rs2239185 (*VDR*)	0.01 (-0.11-0.14)	0.8384	0.9960	0.00 (-0.12-0.12)	0.9699	0.9866	0.04 (-0.08-0.15)	0.5367	0.8848
rs731236 (*VDR*)	0.04 (-0.09-0.17)	0.5352	0.9445	0.03 (-0.09-0.14)	0.6286	0.9795	0.03 (-0.09-0.16)	0.5880	0.9024
rs890945 (Chr 5q33.3)	-0.12 (-0.26-0.01)	0.0775	0.7663	-0.12 (-0.24-0.00)	0.0570	0.6601	-0.12 (-0.25-0.01)	0.0733	0.5471

Variant	Crude Model			Age-Sex Adjusted Model			Fully Adjusted[b] Model		
	β coefficient (95% CI)	P-value	FDR-adjusted P-value	β coefficient (95% CI)	P-value	FDR-adjusted P-value	β coefficient (95% CI)	P-value	FDR-adjusted P-value
Mexican Americans									
rs1042713 (*ADRB2*)	0.00 (-0.17-0.18)	0.9877	0.9877	0.02 (-0.15-0.18)	0.8377	0.9794	-0.01 (-0.15-0.14)	0.9438	0.9466
rs1042714 (*ADRB2*)	-0.01 (-0.15-0.12)	0.8383	0.9877	-0.02 (-0.14-0.10)	0.6964	0.9794	0.00 (-0.12-0.13)	0.9395	0.9466
rs429358 (*APOE*)	0.08 (-0.09-0.24)	0.3568	0.8693	0.06 (-0.09-0.21)	0.4293	0.9794	0.03 (-0.12-0.18)	0.6849	0.9466
rs7412 (*APOE*)	0.06 (-0.13-0.25)	0.5341	0.9454	0.08 (-0.18-0.33)	0.5321	0.9794	-0.01 (-0.25-0.24)	0.9456	0.9466
rs769214 (*CAT*)	0.02 (-0.08-0.11)	0.7372	0.9877	0.02 (-0.06-0.10)	0.6604	0.9794	0.01 (-0.06-0.09)	0.7183	0.9466
rs2280788 (*CCL5*)	0.09 (-0.64-0.83)	0.7940	0.9877	0.05 (-0.67-0.77)	0.8931	0.9794	0.05 (-0.71-0.81)	0.8913	0.9466
rs1799864 (*CCR2*)	-0.06 (-0.25-0.12)	0.4877	0.9454	-0.04 (-0.22-0.15)	0.6874	0.9794	-0.04 (-0.23-0.16)	0.7176	0.9466
rs1205 (*CRP*)	0.00 (-0.11-0.12)	0.9539	0.9877	-0.01 (-0.12-0.09)	0.7885	0.9794	-0.02 (-0.13-0.09)	0.6837	0.9466
rs1417938 (*CRP*)	0.00 (-0.14-0.15)	0.9586	0.9877	-0.01 (-0.14-0.12)	0.8948	0.9794	0.02 (-0.10-0.14)	0.7469	0.9466
rs1800947 (*CRP*)	-0.03 (-0.65-0.59)	0.9155	0.9877	-0.25 (-0.92-0.43)	0.4570	0.9794	-0.35 (-0.77-0.08)	0.1040	0.7038
rs2808630 (*CRP*)	-0.07 (-0.20-0.07)	0.3039	0.8693	-0.04 (-0.17-0.08)	0.4902	0.9794	-0.03 (-0.15-0.09)	0.6347	0.9466
rs3091244 (*CRP*)[c]	-0.01 (-0.27-0.25)<br>0.01 (-0.15-0.16)	0.9719	0.9877	0.05 (-0.19-0.29)<br>-0.00 (-0.15-0.14)	0.8767	0.9794	0.06 (-0.16-0.28)<br>0.02 (-0.11-0.16)	0.7853	0.9466
rs3093058 (*CRP*)	0.08 (-0.41-0.57)	0.7484	0.9877	0.05 (-0.57-0.67)	0.8672	0.9794	0.06 (-0.60-0.72)	0.8539	0.9466
rs3093066 (*CRP*)	-0.31 (-0.72-0.10)	0.1298	0.8653	-0.34 (-0.77-0.09)	0.1143	0.9794	-0.42 (-0.88-0.05)	0.0748	0.7038
rs11265260 (*CRP*)	-0.14 (-0.42-0.14)	0.3209	0.8693	-0.05 (-0.29-0.20)	0.7033	0.9794	-0.02 (-0.26-0.22)	0.8530	0.9466
rs12093699 (*CRP*)	0.02 (-0.11-0.14)	0.7898	0.9877	0.01 (-0.10-0.11)	0.8771	0.9794	0.01 (-0.10-0.12)	0.8355	0.9466
rs12744244 (*CRP*)	0.01 (-0.26-0.27)	0.9614	0.9877	0.00 (-0.24-0.25)	0.9746	0.9931	-0.01 (-0.24-0.21)	0.8990	0.9466
rs2027471 (*CRP*)	-0.02 (-0.13-0.10)	0.7861	0.9877	-0.03 (-0.15-0.08)	0.5546	0.9794	-0.05 (-0.16-0.07)	0.4036	0.9466
rs2592887 (*CRP*)	0.02 (-0.11-0.14)	0.7957	0.9877	-0.00 (-0.12-0.12)	0.9820	0.9931	-0.00 (-0.13-0.12)	0.9466	0.9466
rs2794520 (*CRP*)	-0.01 (-0.12-0.11)	0.9216	0.9877	-0.02 (-0.13-0.09)	0.7030	0.9794	-0.02 (-0.13-0.09)	0.6710	0.9466
rs3093075 (*CRP*)	-0.09 (-0.32-0.13)	0.3983	0.8913	-0.04 (-0.26-0.19)	0.7414	0.9794	-0.04 (-0.26-0.18)	0.7172	0.9466
rs1799963 (*F2*)	0.03 (-0.77-0.83)	0.9361	0.9877	-0.05 (-0.90-0.81)	0.9130	0.9794	-0.04 (-1.01-0.94)	0.9380	0.9466
rs6025 (*F5*)	0.36 (-0.52-1.24)	0.4011	0.8913	0.34 (-0.42-1.11)	0.3658	0.9794	0.33 (-0.43-1.08)	0.3824	0.9466
rs1801274 (*FCGR2A*)	0.07 (-0.04-0.19)	0.2063	0.8693	0.06 (-0.05-0.16)	0.2875	0.9794	0.04 (-0.07-0.15)	0.4347	0.9466
rs1800790 (*FGB*)	-0.02 (-0.19-0.15)	0.8044	0.9877	0.03 (-0.13-0.19)	0.7091	0.9794	0.05 (-0.10-0.20)	0.5023	0.9466
rs1260326 (*GCKR*)	-0.01 (-0.19-0.18)	0.9526	0.9877	-0.01 (-0.18-0.16)	0.8927	0.9794	0.01 (-0.16-0.18)	0.8707	0.9466
rs1143623 (*IL1B*)	0.04 (-0.08-0.15)	0.5276	0.9454	0.09 (-0.02-0.20)	0.1207	0.9794	0.10 (-0.02-0.22)	0.1026	0.7038
rs1800871 (*IL10*)	0.05 (-0.07-0.17)	0.3560	0.8693	0.04 (-0.08-0.16)	0.4878	0.9794	0.03 (-0.10-0.15)	0.6490	0.9466
rs1800872 (*IL10*)	0.06 (-0.07-0.18)	0.3508	0.8693	0.04 (-0.08-0.17)	0.4847	0.9794	0.03 (-0.10-0.16)	0.6401	0.9466
rs1800896 (*IL10*)	-0.13 (-0.28-0.02)	0.0813	0.7637	-0.13 (-0.29-0.02)	0.0909	0.9794	-0.14 (-0.29-0.01)	0.0596	0.7038
rs2243248 (*IL4*)	-0.11 (-0.26-0.04)	0.1565	0.8693	-0.05 (-0.22-0.11)	0.5079	0.9794	-0.06 (-0.19-0.07)	0.3728	0.9466
rs2243250 (*IL4*)	-0.02 (-0.18-0.14)	0.8164	0.9877	-0.00 (-0.16-0.16)	0.9931	0.9931	-0.02 (-0.18-0.13)	0.7587	0.9466
rs2243270 (*IL4*)	-0.04 (-0.21-0.13)	0.6677	0.9877	-0.02 (-0.19-0.14)	0.7986	0.9794	-0.03 (-0.20-0.13)	0.6607	0.9466
rs1801275 (*IL4R*)	0.05 (-0.11-0.21)	0.5357	0.9454	0.08 (-0.10-0.27)	0.3577	0.9794	0.09 (-0.11-0.29)	0.3538	0.9466
rs1805015 (*IL4R*)	0.14 (-0.07-0.34)	0.1813	0.8693	0.15 (-0.06-0.36)	0.1457	0.9794	0.19 (-0.05-0.44)	0.1121	0.7038
rs5918 (*ITGB3*)	0.08 (-0.10-0.26)	0.3543	0.8693	0.10 (-0.09-0.28)	0.2902	0.9794	0.07 (-0.07-0.21)	0.3171	0.9466
rs11003125 (*MBL2*)	-0.09 (-0.23-0.05)	0.2048	0.8693	-0.06 (-0.20-0.07)	0.3598	0.9794	-0.04 (-0.18-0.09)	0.5046	0.9466
rs1800450 (*MBL2*)	0.07 (-0.09-0.24)	0.3622	0.8693	0.04 (-0.12-0.21)	0.5849	0.9794	0.08 (-0.09-0.24)	0.3559	0.9466
rs1800451 (*MBL2*)	-0.08 (-0.44-0.27)	0.6314	0.9877	-0.14 (-0.55-0.27)	0.4978	0.9794	-0.14 (-0.62-0.33)	0.5342	0.9466
rs5030737 (*MBL2*)	0.34 (-0.00-0.68)	0.0528	0.7140	0.36 (-0.04-0.76)	0.0749	0.9794	0.34 (-0.09-0.76)	0.1173	0.7038
rs7096206 (*MBL2*)	0.16 (-0.03-0.34)	0.0891	0.7637	0.12 (-0.09-0.34)	0.2551	0.9794	0.07 (-0.15-0.28)	0.5370	0.9466
rs1800482 (*NOS2A*)	-0.06 (-0.69-0.57)	0.8458	0.9877	0.04 (-0.63-0.70)	0.9100	0.9794	-0.32 (-0.58--0.06)	**0.0176**	0.3520
rs9282799 (*NOS2A*)	0.67 (0.29-1.05)	**0.0014**	0.0840	0.59 (-0.24-1.42)	0.1567	0.9794	0.58 (-0.44-1.59)	0.2513	0.9466
rs1799983 (*NOS3*)	-0.12 (-0.30-0.06)	0.1699	0.8693	-0.16 (-0.32--0.00)	**0.0460**	0.9794	-0.18 (-0.32--0.04)	**0.0145**	0.3520
rs2070744 (*NOS3*)	-0.06 (-0.24-0.13)	0.5185	0.9454	-0.09 (-0.27-0.10)	0.3492	0.9794	-0.10 (-0.28-0.07)	0.2417	0.9466
rs662 (*PON1*)	-0.09 (-0.24-0.06)	0.2461	0.8693	-0.07 (-0.20-0.06)	0.3053	0.9794	-0.05 (-0.18-0.07)	0.3835	0.9466
rs854560 (*PON1*)	-0.04 (-0.29-0.21)	0.7568	0.9877	-0.07 (-0.29-0.16)	0.5443	0.9794	-0.07 (-0.29-0.15)	0.5209	0.9466
rs1801282 (*PPARG*)	-0.12 (-0.35-0.11)	0.2953	0.8693	-0.11 (-0.33-0.12)	0.3358	0.9794	-0.10 (-0.32-0.13)	0.3710	0.9466
rs1799762 (*SERPINE1*)	0.10 (0.01-0.19)	**0.0295**	0.5900	0.06 (-0.04-0.15)	0.2116	0.9794	0.03 (-0.07-0.12)	0.5637	0.9466
rs1800468 (*TGFB1/B9D2*)	-0.12 (-0.43-0.19)	0.4388	0.9403	-0.18 (-0.46-0.10)	0.1938	0.9794	-0.16 (-0.44-0.12)	0.2563	0.9466
rs1800469 (*TGFB1/B9D2*)	0.00 (-0.14-0.14)	0.9566	0.9877	0.01 (-0.12-0.14)	0.8871	0.9794	0.04 (-0.08-0.17)	0.5033	0.9466
rs1800470 (*TGFB1*)	0.04 (-0.10-0.18)	0.5584	0.9573	0.04 (-0.10-0.19)	0.5347	0.9794	0.08 (-0.08-0.24)	0.3019	0.9466
rs4986790 (*TLR4*)	-0.24 (-0.56-0.07)	0.1199	0.8653	-0.27 (-0.61-0.06)	0.1042	0.9794	-0.30 (-0.69-0.10)	0.1315	0.7173
rs1800629 (*TNF*)	0.08 (-0.15-0.32)	0.4774	0.9454	0.00 (-0.22-0.23)	0.9819	0.9931	0.02 (-0.22-0.25)	0.8907	0.9466
rs1800750 (*TNF*)	0.27 (-0.19-0.74)	0.2328	0.8693	0.21 (-0.25-0.68)	0.3531	0.9794	0.28 (-0.11-0.66)	0.1522	0.7610
rs361525 (*TNF*)	0.04 (-0.29-0.37)	0.7862	0.9877	0.04 (-0.29-0.36)	0.8213	0.9794	0.05 (-0.25-0.35)	0.7486	0.9466
rs2239185 (*VDR*)	-0.11 (-0.23-0.00)	0.0595	0.7140	-0.10 (-0.22-0.02)	0.0930	0.9794	-0.11 (-0.24-0.02)	0.0945	0.7038
rs731236 (*VDR*)	0.00 (-0.15-0.16)	0.9627	0.9877	0.01 (-0.14-0.16)	0.8841	0.9794	0.01 (-0.16-0.18)	0.8907	0.9466
rs890945 (Chr 5q33.3)	-0.11 (-0.33-0.10)	0.2903	0.8693	-0.10 (-0.32-0.13)	0.3815	0.9794	-0.10 (-0.32-0.12)	0.3547	0.9466

CI, confidence interval; FDR, false-discovery rate.

a) Defined as log-transformed albumin-to-creatinine ratio (ACR) and analyzed as a continuous variable. b) Analyses adjusted for age, sex, alcohol consumption, educational attainment, and waist:hip ratio. c) For this tri-allelic variant, the first beta coefficient corresponds to the A allele, while the second corresponds to the T allele. (The C allele is the reference.) The unadjusted and FDR-adjusted P values are for the overall test of association.

Table S2. Complete results of associations of candidate gene polymorphisms and albumin-to-creatinine ratio[a], codominant genetic model

Variant	Genotype	Crude Model			Age-Sex Adjusted Model			Fully Adjusted[b] Model		
		β coefficient (95% CI)	P-value	FDR-adjusted P-value	β coefficient (95% CI)	P-value	FDR-adjusted P-value	β coefficient (95% CI)	P-value	FDR-adjusted P-value
Non-Hispanic whites										
rs1042713 (*ADRB2*)	AA vs. GG<br>AG vs. GG	0.02 (-0.13-0.17)<br>0.04 (-0.11-0.19)	0.8020	0.8864	0.04 (-0.08-0.17)<br>0.04 (-0.08-0.17)	0.6492	0.8254	0.04 (-0.10-0.19)<br>0.05 (-0.07-0.18)	0.5950	0.7835
rs1042714 (*ADRB2*)	GG vs. CC<br>GC vs. CC	-0.03 (-0.18-0.11)<br>0.01 (-0.15-0.17)	0.8465	0.9092	-0.04 (-0.18-0.09)<br>-0.02 (-0.16-0.11)	0.8087	0.8398	-0.06 (-0.22-0.09)<br>-0.01 (-0.16-0.14)	0.7526	0.8236
rs429358 (*APOE*)	CC vs. TT<br>CT vs. TT	0.20 (-0.06-0.46)<br>-0.04 (-0.21-0.12)	0.3797	0.6514	0.24 (-0.03-0.50)<br>-0.02 (-0.16-0.12)	0.3366	0.6537	0.22 (-0.03-0.47)<br>-0.03 (-0.17-0.11)	0.3411	0.6855
rs7412 (*APOE*)	TT vs. CC<br>TC vs. CC	0.24 (-0.34-0.83)<br>0.11 (-0.04-0.26)	0.2261	0.6514	0.25 (-0.20-0.70)<br>0.08 (-0.04-0.21)	0.2195	0.6230	0.22 (-0.27-0.71)<br>0.07 (-0.07-0.21)	0.4064	0.6933
rs769214 (*CAT*)	GG vs. AA<br>GA vs. AA	-0.06 (-0.35-0.23)<br>-0.09 (-0.25-0.07)	0.5750	0.7756	-0.06 (-0.32-0.20)<br>-0.10 (-0.22-0.02)	0.3778	0.6537	-0.06 (-0.35-0.22)<br>-0.10 (-0.22-0.03)	0.4265	0.7068
rs2280788 (*CCL5*)	GG vs. CC<br>GC vs. CC	0.01 (-1.09-1.12)<br>0.05 (-0.17-0.26)	0.9083	0.9306	-0.02 (-1.31-1.28)<br>0.11 (-0.11-0.32)	0.6460	0.8254	0.09 (-1.80-1.98)<br>0.08 (-0.14-0.30)	0.8102	0.8544
rs1799864 (*CCR2*)	AA vs. GG<br>AG vs. GG	-0.47 (-1.37-0.43)<br>0.09 (0.01-0.18)	0.2288	0.6514	-0.36 (-1.21-0.49)<br>0.07 (-0.03-0.16)	0.3568	0.6537	-0.45 (-1.54-0.63)<br>0.09 (-0.01-0.20)	0.3251	0.6855
rs1205 (*CRP*)	AA vs. GG<br>AG vs. GG	0.01 (-0.18-0.20)<br>-0.05 (-0.21-0.11)	0.7471	0.8558	0.05 (-0.12-0.21)<br>-0.06 (-0.21-0.09)	0.4670	0.6698	0.05 (-0.09-0.20)<br>-0.06 (-0.23-0.12)	0.5005	0.7639
rs1417938 (*CRP*)	TT vs. AA<br>TA vs. AA	0.25 (0.04-0.47)<br>-0.00 (-0.14-0.14)	0.0801	0.6351	0.24 (0.04-0.43)<br>-0.01 (-0.13-0.12)	0.0643	0.4880	0.17 (-0.01-0.36)<br>-0.02 (-0.14-0.11)	0.1944	0.6855
rs1800947 (*CRP*)	CC vs. GG<br>CG vs. GG									
rs2808630 (*CRP*)	GG vs. AA<br>GA vs. AA	-0.12 (-0.34-0.11)<br>-0.06 (-0.19-0.08)	0.4720	0.7399	-0.09 (-0.30-0.12)<br>-0.07 (-0.17-0.03)	0.3803	0.6537	-0.08 (-0.29-0.12)<br>-0.04 (-0.15-0.07)	0.6025	0.7835
rs3091244 (*CRP*)	AA vs. CC<br>AC vs. CC<br>AT vs. CC<br>CT vs. CC<br>TT vs. CC	0.54 (-0.55-1.62)<br>0.07 (-0.16-0.31)<br>-0.03 (-0.36-0.30)<br>0.01 (-0.17-0.19)<br>0.24 (0.03-0.44)	0.3128	0.6514	0.40 (-0.74-1.55)<br>0.04 (-0.20-0.28)<br>-0.01 (-0.32-0.31)<br>-0.01 (-0.16-0.15)<br>0.23 (0.04-0.41)	0.3593	0.6537	0.34 (-0.79-1.48)<br>0.06 (-0.20-0.31)<br>-0.02 (-0.35-0.31)<br>-0.00 (-0.16-0.16)<br>0.18 (-0.01-0.36)	0.6396	0.7835
rs3093058 (*CRP*)	TT vs. AA<br>TA vs. AA	0.45 (-0.42-1.31)	0.2977	0.6514	0.53 (-0.22-1.28)	0.1583	0.6230	0.46 (-0.35-1.27)	0.2500	0.6855
rs3093066 (*CRP*)	AA vs. CC<br>AC vs. CC	-0.32 (-0.93-0.29)	0.2857	0.6514	-0.26 (-0.88-0.37)	0.4017	0.6542	-0.18 (-0.78-0.43)	0.5539	0.7835
rs11265260 (*CRP*)	GG vs. AA<br>GA vs. AA	0.08 (-0.51-0.67)<br>0.05 (-0.10-0.20)	0.6505	0.8212	-0.04 (-0.78-0.70)<br>0.01 (-0.13-0.16)	0.9727	0.9727	-0.10 (-0.81-0.61)<br>0.01 (-0.14-0.16)	0.9491	0.9491
rs12093699 (*CRP*)	AA vs. GG<br>AG vs. GG	0.22 (-0.03-0.46)<br>0.04 (-0.15-0.24)	0.2881	0.6514	0.20 (-0.01-0.42)<br>0.03 (-0.14-0.19)	0.2479	0.6230	0.17 (-0.06-0.40)<br>0.03 (-0.12-0.19)	0.3810	0.6855
rs12744244 (*CRP*)	AA vs. CC<br>AC vs. CC	0.10 (-0.26-0.46)<br>0.12 (-0.05-0.29)	0.3141	0.6514	0.12 (-0.22-0.46)<br>0.08 (-0.07-0.24)	0.4700	0.6698	0.01 (-0.35-0.37)<br>0.10 (-0.03-0.23)	0.3312	0.6855
rs2027471 (*CRP*)	AA vs. TT<br>AT vs. TT	-0.00 (-0.20-0.19)<br>-0.07 (-0.24-0.10)	0.6281	0.8212	0.03 (-0.13-0.18)<br>-0.07 (-0.23-0.09)	0.4367	0.6698	0.04 (-0.10-0.18)<br>-0.06 (-0.24-0.12)	0.4838	0.7639
rs2592887 (*CRP*)	AA vs. GG<br>AG vs. GG	-0.03 (-0.20-0.14)<br>-0.04 (-0.20-0.12)	0.8100	0.8864	-0.03 (-0.19-0.14)<br>-0.05 (-0.22-0.12)	0.7134	0.8254	-0.01 (-0.17-0.15)<br>-0.06 (-0.25-0.12)	0.6088	0.7835
rs2794520 (*CRP*)	AA vs. GG<br>AG vs. GG	0.04 (-0.17-0.25)<br>-0.06 (-0.23-0.10)	0.5321	0.7667	0.08 (-0.10-0.26)<br>-0.08 (-0.22-0.07)	0.2425	0.6230	0.08 (-0.08-0.24)<br>-0.07 (-0.24-0.10)	0.3341	0.6855
rs3093075 (*CRP*)	AA vs. CC<br>AC vs. CC	0.51 (-0.57-1.60)<br>0.01 (-0.13-0.15)	0.5080	0.7667	0.39 (-0.75-1.54)<br>-0.02 (-0.16-0.12)	0.6522	0.8254	0.34 (-0.81-1.48)<br>-0.01 (-0.16-0.14)	0.7361	0.8236
rs1799963 (*F2*)	AG vs. GG	0.29 (-0.14-0.72)	0.1777	0.6514	0.17 (-0.28-0.61)	0.4427	0.6698	0.22 (-0.25-0.69)	0.3499	0.6855
rs6025 (*F5*)	AA vs. GG<br>AG vs. GG									
rs1801274 (*FCGR2A*)	AA vs. GG<br>AG vs. GG	0.13 (-0.02-0.29)<br>0.10 (-0.08-0.28)	0.3175	0.6514	0.16 (0.02-0.30)<br>0.15 (-0.01-0.30)	0.0827	0.4880	0.18 (0.02-0.34)<br>0.17 (-0.01-0.34)	0.0683	0.4324
rs1800790 (*FGB*)	AA vs. GG<br>AG vs. GG	0.07 (-0.19-0.33)<br>-0.13 (-0.25--0.02)	**0.0473**	0.5487	0.06 (-0.18-0.30)<br>-0.12 (-0.22--0.02)	0.0573	0.4880	0.02 (-0.22-0.26)<br>-0.14 (-0.27--0.01)	0.0620	0.4324
rs1260326 (*GCKR*)	TT vs. CC<br>TC vs. CC	0.02 (-0.18-0.21)<br>0.10 (-0.04-0.25)	0.2984	0.6514	0.07 (-0.10-0.24)<br>0.11 (-0.03-0.25)	0.2763	0.6300	0.08 (-0.09-0.25)<br>0.11 (-0.03-0.24)	0.2738	0.6855
rs1143623 (*IL1B*)	CC vs. GG<br>CG vs. GG	-0.13 (-0.44-0.18)<br>0.05 (-0.08-0.19)	0.3931	0.6514	-0.07 (-0.38-0.23)<br>0.04 (-0.10-0.18)	0.6802	0.8254	-0.09 (-0.36-0.19)<br>0.06 (-0.08-0.19)	0.4951	0.7639
rs1800871 (*IL10*)	TT vs. CC<br>TC vs. CC	-0.12 (-0.29-0.06)<br>0.08 (-0.04-0.21)	0.1282	0.6351	-0.11 (-0.28-0.06)<br>0.09 (-0.03-0.21)	0.1113	0.4880	-0.08 (-0.24-0.07)<br>0.10 (-0.02-0.23)	0.0804	0.4324
rs1800872 (*IL10*)	AA vs. CC<br>AC vs. CC	-0.14 (-0.31-0.03)<br>0.08 (-0.05-0.21)	0.1148	0.6351	-0.12 (-0.28-0.05)<br>0.08 (-0.04-0.21)	0.1099	0.4880	-0.09 (-0.24-0.06)<br>0.10 (-0.02-0.23)	0.0820	0.4324
rs1800896 (*IL10*)	GG vs. AA<br>GA vs. AA	-0.05 (-0.19-0.09)<br>0.10 (-0.03-0.22)	0.1267	0.6351	-0.06 (-0.20-0.08)<br>0.07 (-0.06-0.20)	0.1743	0.6230	-0.05 (-0.19-0.08)<br>0.07 (-0.07-0.21)	0.2136	0.6855
rs2243248 (*IL4*)	GG vs. TT<br>GT vs. TT	-0.28 (-1.39-0.84)<br>0.05 (-0.14-0.23)	0.7312	0.8558	-0.14 (-1.08-0.81)<br>0.14 (-0.02-0.29)	0.2416	0.6230	-0.17 (-1.01-0.67)<br>0.13 (-0.05-0.30)	0.2763	0.6855
rs2243250 (*IL4*)	TT vs. CC<br>TC vs. CC	-0.14 (-0.38-0.11)<br>-0.07 (-0.20-0.07)	0.3661	0.6514	-0.15 (-0.42-0.12)<br>-0.08 (-0.21-0.04)	0.2460	0.6230	-0.21 (-0.52-0.11)<br>-0.07 (-0.18-0.05)	0.2509	0.6855
rs2243270 (*IL4*)	GG vs. AA<br>GA vs. AA	-0.11 (-0.36-0.15)<br>-0.07 (-0.21-0.06)	0.3714	0.6514	-0.11 (-0.37-0.14)<br>-0.09 (-0.22-0.04)	0.2212	0.6230	-0.14 (-0.46-0.18)<br>-0.08 (-0.21-0.04)	0.2929	0.6855
rs1801275 (*IL4R*)	GG vs. AA<br>GA vs. AA	0.17 (-0.13-0.47)<br>-0.12 (-0.26-0.02)	0.1014	0.6351	0.24 (-0.08-0.57)<br>-0.11 (-0.23-0.01)	0.0533	0.4880	0.24 (-0.11-0.58)<br>-0.08 (-0.22-0.05)	0.1301	0.6166

Variant	Genotype	Crude Model			Age-Sex Adjusted Model			Fully Adjusted[b] Model		
		β coefficient (95% CI)	P-value	FDR-adjusted P-value	β coefficient (95% CI)	P-value	FDR-adjusted P-value	β coefficient (95% CI)	P-value	FDR-adjusted P-value
rs1805015 (*IL4R*)	CC vs. TT<br>CT vs. TT	0.30 (-0.10-0.70)<br>-0.08 (-0.21-0.05)	0.1314	0.6351	0.33 (-0.06-0.73)<br>-0.07 (-0.19-0.05)	0.0967	0.4880	0.33 (-0.10-0.76)<br>-0.05 (-0.16-0.06)	0.1382	0.6166
rs5918 (*ITGB3*)	CC vs. TT<br>CT vs. TT	0.04 (-0.33-0.41)<br>0.00 (-0.14-0.15)	0.9452	0.9452	0.11 (-0.22-0.45)<br>-0.01 (-0.14-0.13)	0.7617	0.8254	0.15 (-0.19-0.48)<br>-0.02 (-0.15-0.12)	0.6101	0.7835
rs11003125 (*MBL2*)	GG vs. CC<br>GC vs. CC	0.25 (0.08-0.41)<br>0.05 (-0.06-0.16)	**0.0234**	0.4524	0.21 (0.06-0.37)<br>0.03 (-0.05-0.12)	**0.0255**	0.4845	0.23 (0.07-0.38)<br>0.01 (-0.08-0.11)	**0.0166**	0.4324
rs1800450 (*MBL2*)	AA vs. GG<br>AG vs. GG	0.13 (-0.20-0.46)<br>0.06 (-0.09-0.20)	0.5420	0.7667	0.10 (-0.26-0.47)<br>0.03 (-0.10-0.15)	0.7578	0.8254	0.13 (-0.24-0.50)<br>0.01 (-0.13-0.15)	0.7452	0.8236
rs1800451 (*MBL2*)	AA vs. GG<br>AG vs. GG	1.67 (0.96-2.38)<br>-0.10 (-0.39-0.19)	0.0707	0.6351	1.50 (0.95-2.05)<br>-0.13 (-0.41-0.15)	0.0692	0.4880	1.53 (0.92-2.14)<br>-0.12 (-0.40-0.17)	0.0749	0.4324
rs5030737 (*MBL2*)	TT vs. CC<br>TC vs. CC	0.09 (-1.05-1.22)<br>0.24 (-0.02-0.49)	0.1599	0.6514	-0.07 (-1.08-0.95)<br>0.25 (0.01-0.49)	0.0911	0.4880	-0.02 (-1.06-1.03)<br>0.26 (0.03-0.49)	0.0820	0.4324
rs7096206 (*MBL2*)	CC vs. GG<br>CG vs. GG	0.21 (0.03-0.38)<br>-0.13 (-0.28-0.01)	**0.0231**	0.4524	0.18 (-0.00-0.36)<br>-0.13 (-0.23--0.03)	**0.0049**	0.1397	0.24 (0.01-0.46)<br>-0.13 (-0.22--0.04)	**0.0031**	0.1798
rs1800482 (*NOS2A*)	CC vs. GG<br>CG vs. GG	0.43 (-0.97-1.84)	0.5304	0.7667	0.15 (-1.15-1.46)	0.8103	0.8398	0.18 (-1.04-1.39)	0.7686	0.8255
rs9282799 (*NOS2A*)	TT vs. CC<br>TC vs. CC	1.31 (0.58-2.04)	**0.0011**	0.0638	0.89 (0.06-1.72)	**0.0374**	0.4880	0.81 (0.00-1.62)	**0.0490**	0.4324
rs1799983 (*NOS3*)	TT vs. GG<br>TG vs. GG	0.01 (-0.24-0.27)<br>0.03 (-0.10-0.17)	0.8756	0.9234	0.06 (-0.16-0.28)<br>0.05 (-0.09-0.18)	0.7377	0.8254	0.05 (-0.16-0.27)<br>0.06 (-0.07-0.19)	0.6484	0.7835
rs2070744 (*NOS3*)	CC vs. TT<br>CT vs. TT	-0.09 (-0.26-0.09)<br>0.01 (-0.19-0.20)	0.6513	0.8212	-0.07 (-0.25-0.12)<br>0.01 (-0.17-0.20)	0.7040	0.8254	-0.07 (-0.27-0.12)<br>0.01 (-0.17-0.19)	0.7092	0.8236
rs662 (*PON1*)	GG vs. AA<br>GA vs. AA	-0.06 (-0.26-0.14)<br>-0.11 (-0.27-0.04)	0.2630	0.6514	-0.03 (-0.20-0.15)<br>-0.08 (-0.24-0.08)	0.4548	0.6698	-0.00 (-0.17-0.16)<br>-0.06 (-0.20-0.08)	0.5752	0.7835
rs854560 (*PON1*)	AA vs. TT<br>AT vs. TT	0.03 (-0.19-0.26)<br>0.11 (0.02-0.20)	0.2462	0.6514	0.04 (-0.13-0.22)<br>0.08 (-0.02-0.17)	0.3713	0.6537	0.02 (-0.15-0.20)<br>0.08 (-0.03-0.19)	0.3900	0.6855
rs1801282 (*PPARG*)	GG vs. CC<br>GC vs. CC	0.17 (-0.35-0.70)<br>0.07 (-0.10-0.25)	0.5716	0.7756	0.30 (-0.09-0.69)<br>0.06 (-0.09-0.22)	0.2514	0.6230	0.36 (-0.07-0.80)<br>0.09 (-0.08-0.26)	0.1871	0.6855
rs1799762 (*SERPINE1*)	4G4G vs. 5G5G<br>4G5G vs. 5G5G	0.07 (-0.12-0.27)<br>0.12 (-0.03-0.26)	0.3425	0.6514	0.09 (-0.10-0.28)<br>0.10 (-0.03-0.23)	0.3899	0.6537	0.10 (-0.11-0.30)<br>0.12 (-0.01-0.26)	0.3297	0.6855
rs1800468 (*TGFB1/B9D2*)	AA vs. GG<br>AG vs. GG	0.56 (-0.07-1.20)<br>0.02 (-0.17-0.22)	0.3430	0.6514	0.53 (-0.18-1.23)<br>0.05 (-0.12-0.22)	0.3065	0.6471	0.48 (-0.25-1.21)<br>0.05 (-0.12-0.22)	0.3579	0.6855
rs1800469 (*TGFB1/B9D2*)	TT vs. CC<br>TC vs. CC	-0.07 (-0.32-0.19)<br>0.06 (-0.07-0.20)	0.3895	0.6514	-0.06 (-0.29-0.17)<br>0.05 (-0.08-0.18)	0.4988	0.6935	-0.05 (-0.31-0.20)<br>0.05 (-0.08-0.18)	0.5583	0.7835
rs1800470 (*TGFB1*)	CC vs. TT<br>CT vs. TT	0.02 (-0.24-0.28)<br>0.11 (-0.02-0.23)	0.2901	0.6514	0.00 (-0.25-0.25)<br>0.10 (-0.01-0.20)	0.3065	0.6471	0.04 (-0.22-0.29)<br>0.10 (-0.01-0.21)	0.3429	0.6855
rs4986790 (*TLR4*)	GG vs. AA<br>GA vs. AA	0.02 (-0.92-0.95)<br>0.08 (-0.14-0.31)	0.7525	0.8558	0.06 (-0.76-0.89)<br>0.08 (-0.12-0.27)	0.7247	0.8254	0.05 (-0.75-0.85)<br>0.07 (-0.12-0.27)	0.7514	0.8236
rs1800629 (*TNF*)	AA vs. GG<br>AG vs. GG	-0.03 (-0.38-0.31)<br>-0.10 (-0.27-0.07)	0.4381	0.7058	0.02 (-0.32-0.35)<br>-0.11 (-0.27-0.04)	0.2750	0.6300	-0.01 (-0.36-0.33)<br>-0.11 (-0.28-0.07)	0.3867	0.6855
rs1800750 (*TNF*)	AA vs. GG<br>AG vs. GG	1.34 (-0.22-2.90)<br>0.37 (0.01-0.73)	**0.0369**	0.5351	1.20 (0.39-2.01)<br>0.44 (0.13-0.75)	**0.0031**	0.1397	1.23 (0.64-1.83)<br>0.29 (-0.04-0.63)	**0.0224**	0.4324
rs361525 (*TNF*)	AA vs. GG<br>AG vs. GG	0.71 (-0.17-1.59)<br>0.04 (-0.19-0.27)	0.2184	0.6514	0.72 (-0.19-1.62)<br>0.06 (-0.14-0.27)	0.1888	0.6230	1.00 (0.12-1.88)<br>0.02 (-0.13-0.17)	**0.0356**	0.4324
rs2239185 (*VDR*)	CC vs. TT<br>CT vs. TT	-0.02 (-0.17-0.14)<br>0.03 (-0.11-0.17)	0.7413	0.8558	0.01 (-0.16-0.18)<br>0.04 (-0.10-0.18)	0.7675	0.8254	0.05 (-0.11-0.20)<br>0.06 (-0.09-0.22)	0.6222	0.7835
rs731236 (*VDR*)	CC vs. TT<br>CT vs. TT	0.07 (-0.13-0.27)<br>0.00 (-0.10-0.11)	0.6947	0.8558	0.03 (-0.15-0.22)<br>-0.01 (-0.12-0.10)	0.8365	0.8514	-0.00 (-0.19-0.19)<br>-0.02 (-0.13-0.09)	0.9276	0.9439
rs890945 (Chr 5q33.3)	AA vs. TT<br>AT vs. TT	0.06 (-0.34-0.45)<br>0.02 (-0.11-0.15)	0.9146	0.9306	0.11 (-0.21-0.43)<br>0.04 (-0.08-0.16)	0.6391	0.8254	0.03 (-0.32-0.38)<br>0.04 (-0.09-0.17)	0.8264	0.8559
Non-Hispanic blacks										
rs1042713 (*ADRB2*)	AA vs. GG<br>AG vs. GG	0.00 (-0.30-0.30)<br>-0.02 (-0.23-0.19)	0.9669	0.9944	0.05 (-0.23-0.33)<br>-0.01 (-0.19-0.18)	0.8283	0.8908	0.05 (-0.22-0.33)<br>0.05 (-0.14-0.25)	0.8460	0.8800
rs1042714 (*ADRB2*)	GG vs. CC<br>GC vs. CC	-0.35 (-0.78-0.08)<br>0.07 (-0.21-0.36)	0.3358	0.7950	-0.27 (-0.69-0.15)<br>-0.01 (-0.28-0.26)	0.5600	0.7956	-0.27 (-0.68-0.14)<br>-0.03 (-0.29-0.23)	0.5443	0.7517
rs429358 (*APOE*)	CC vs. TT<br>CT vs. TT	0.10 (-0.37-0.56)<br>0.00 (-0.19-0.19)	0.8973	0.9944	0.08 (-0.28-0.43)<br>0.09 (-0.09-0.26)	0.5513	0.7956	0.02 (-0.26-0.29)<br>0.08 (-0.07-0.24)	0.4753	0.7354
rs7412 (*APOE*)	TT vs. CC<br>TC vs. CC	-0.25 (-1.02-0.53)<br>0.16 (-0.05-0.36)	0.1861	0.7950	-0.20 (-0.82-0.42)<br>0.11 (-0.04-0.25)	0.2275	0.7656	-0.18 (-0.83-0.47)<br>0.14 (-0.04-0.31)	0.1909	0.6484
rs769214 (*CAT*)	GG vs. AA<br>GA vs. AA	0.03 (-0.22-0.27)<br>-0.09 (-0.30-0.12)	0.5744	0.8723	0.01 (-0.23-0.25)<br>-0.10 (-0.28-0.08)	0.4859	0.7956	0.01 (-0.21-0.22)<br>-0.11 (-0.30-0.07)	0.3371	0.7234
rs2280788 (*CCL5*)	GG vs. CC<br>GC vs. CC	-0.31 (-0.82-0.21)	0.2290	0.7950	-0.09 (-0.60-0.43)	0.7266	0.8283	-0.06 (-0.62-0.51)	0.8339	0.8800
rs1799864 (*CCR2*)	AA vs. GG<br>AG vs. GG	-0.05 (-0.83-0.72)<br>-0.05 (-0.25-0.15)	0.8763	0.9944	-0.02 (-0.61-0.57)<br>-0.04 (-0.21-0.13)	0.8835	0.9092	-0.14 (-0.63-0.35)<br>-0.03 (-0.20-0.14)	0.7952	0.8800
rs1205 (*CRP*)	AA vs. GG<br>AG vs. GG	-0.34 (-0.81-0.13)<br>-0.04 (-0.22-0.14)	0.2714	0.7950	-0.46 (-0.86--0.05)<br>-0.00 (-0.16-0.16)	0.0539	0.7656	-0.51 (-0.98--0.04)<br>-0.04 (-0.21-0.14)	0.0659	0.6484
rs1417938 (*CRP*)	TT vs. AA<br>TA vs. AA	-0.73 (-1.16--0.30)<br>0.07 (-0.23-0.37)	0.1658	0.7950	-0.75 (-1.36--0.14)<br>0.12 (-0.21-0.46)	0.1747	0.7656	-0.55 (-1.04--0.06)<br>0.17 (-0.16-0.51)	0.1776	0.6484
rs1800947 (*CRP*)	CC vs. GG<br>CG vs. GG	-0.44 (-1.52-0.64)	0.4109	0.7950	-0.60 (-1.50-0.30)	0.1830	0.7656	-0.55 (-1.44-0.34)	0.2124	0.6484
rs2808630 (*CRP*)	GG vs. AA<br>GA vs. AA	0.42 (-0.16-1.00)<br>0.08 (-0.13-0.28)	0.2892	0.7950	0.26 (-0.20-0.72)<br>0.05 (-0.11-0.21)	0.4548	0.7956	0.14 (-0.19-0.47)<br>0.03 (-0.15-0.21)	0.7077	0.8800

Variant	Genotype	Crude Model			Age-Sex Adjusted Model			Fully Adjusted[b] Model		
		**β coefficient (95% CI)**	**P-value**	**FDR-adjusted P-value**	**β coefficient (95% CI)**	**P-value**	**FDR-adjusted P-value**	**β coefficient (95% CI)**	**P-value**	**FDR-adjusted P-value**
rs3091244 (*CRP*)	AA vs. CC<br>AC vs. CC<br>AT vs. CC<br>CT vs. CC<br>TT vs. CC	-0.04 (-0.25-0.17)<br>0.08 (-0.16-0.31)<br>0.01 (-0.31-0.33)<br>-0.11 (-0.37-0.16)<br>-0.23 (-0.66-0.19)	0.4578	0.8298	0.03 (-0.14-0.20)<br>0.13 (-0.07-0.32)<br>0.07 (-0.13-0.27)<br>0.01 (-0.24-0.25)<br>-0.18 (-0.63-0.27)	0.5121	0.7956	0.06 (-0.15-0.27)<br>0.21 (0.02-0.40)<br>0.15 (-0.06-0.37)<br>0.04 (-0.26-0.33)<br>-0.03 (-0.49-0.43)	0.4655	0.7354
rs3093058 (*CRP*)	TT vs. AA<br>TA vs. AA	-0.31 (-0.83-0.22)<br>-0.17 (-0.39-0.05)	0.1659	0.7950	-0.28 (-0.83-0.27)<br>-0.14 (-0.34-0.06)	0.2616	0.7656	-0.23 (-0.83-0.37)<br>-0.15 (-0.37-0.06)	0.2800	0.6767
rs3093066 (*CRP*)	AA vs. CC<br>AC vs. CC	-0.03 (-0.24-0.17)<br>0.20 (-0.06-0.45)	0.1253	0.7950	0.06 (-0.17-0.29)<br>0.19 (-0.01-0.39)	0.0717	0.7656	-0.01 (-0.24-0.23)<br>0.23 (0.03-0.44)	**0.0277**	0.6484
rs11265260 (*CRP*)	GG vs. AA<br>GA vs. AA	0.77 (-1.29-2.83)<br>-0.08 (-0.33-0.16)	0.5297	0.8723	0.86 (-0.70-2.43)<br>-0.09 (-0.35-0.18)	0.3988	0.7838	0.89 (-0.65-2.42)<br>-0.04 (-0.32-0.24)	0.4393	0.7354
rs12093699 (*CRP*)	AA vs. GG<br>AG vs. GG	0.19 (-0.11-0.48)<br>0.07 (-0.12-0.26)	0.3767	0.7950	0.18 (-0.10-0.45)<br>0.09 (-0.10-0.28)	0.3127	0.7656	0.17 (-0.12-0.46)<br>0.07 (-0.13-0.27)	0.4241	0.7354
rs12744244 (*CRP*)	AA vs. CC<br>AC vs. CC	-0.85 (-1.53--0.17)<br>0.05 (-0.35-0.45)	0.4249	0.7950	-0.54 (-1.13-0.05)<br>0.08 (-0.33-0.50)	0.5452	0.7956	-0.50 (-1.11-0.10)<br>0.12 (-0.29-0.53)	0.4701	0.7354
rs2027471 (*CRP*)	AA vs. TT<br>AT vs. TT	-0.16 (-0.56-0.24)<br>-0.12 (-0.31-0.07)	0.3941	0.7950	-0.28 (-0.66-0.10)<br>-0.07 (-0.24-0.10)	0.2689	0.7656	-0.37 (-0.84-0.10)<br>-0.09 (-0.27-0.09)	0.1865	0.6484
rs2592887 (*CRP*)	AA vs. GG<br>AG vs. GG	0.15 (-0.22-0.52)<br>0.05 (-0.22-0.32)	0.5996	0.8723	0.12 (-0.19-0.43)<br>0.09 (-0.12-0.31)	0.6368	0.7956	0.07 (-0.24-0.39)<br>0.05 (-0.20-0.31)	0.8800	0.8800
rs2794520 (*CRP*)	AA vs. GG<br>AG vs. GG	-0.14 (-0.53-0.26)<br>-0.07 (-0.26-0.11)	0.6141	0.8723	-0.24 (-0.61-0.14)<br>-0.02 (-0.19-0.15)	0.4168	0.7919	-0.32 (-0.79-0.16)<br>-0.04 (-0.22-0.14)	0.3186	0.7234
rs3093075 (*CRP*)	AA vs. CC<br>AC vs. CC	-0.09 (-0.31-0.13)<br>0.18 (-0.06-0.43)	0.1166	0.7950	-0.05 (-0.28-0.17)<br>0.16 (-0.04-0.36)	0.1146	0.7656	-0.06 (-0.33-0.20)<br>0.22 (0.03-0.41)	**0.0304**	0.6484
rs1799963 (*F2*)	AG vs. GG	-0.88 (-2.20-0.44)	0.1810	0.7950	-1.04 (-1.90--0.18)	**0.0205**	0.7656	-0.96 (-1.88--0.04)	**0.0411**	0.6484
rs6025 (*F5*)	AA vs. GG<br>AG vs. GG	 0.07 (-0.43-0.56)	0.7817	0.9647	 0.23 (-0.27-0.73)	0.3442	0.7656	 0.18 (-0.43-0.80)	0.5440	0.7517
rs1801274 (*FCGR2A*)	AA vs. GG<br>AG vs. GG	0.18 (-0.14-0.51)<br>0.14 (-0.09-0.37)	0.4243	0.7950	0.24 (-0.08-0.56)<br>0.17 (-0.07-0.42)	0.2229	0.7656	0.23 (-0.10-0.55)<br>0.17 (-0.10-0.43)	0.2515	0.6767
rs1800790 (*FGB*)	AA vs. GG<br>AG vs. GG	0.68 (-0.05-1.41)<br>-0.13 (-0.42-0.16)	0.1696	0.7950	0.56 (0.04-1.07)<br>-0.08 (-0.36-0.21)	0.2685	0.7656	0.65 (0.17-1.13)<br>0.04 (-0.33-0.40)	0.3617	0.7234
rs1260326 (*GCKR*)	TT vs. CC<br>TC vs. CC	-0.03 (-0.43-0.36)<br>0.05 (-0.16-0.26)	0.7997	0.9663	-0.12 (-0.47-0.23)<br>0.00 (-0.21-0.21)	0.7873	0.8799	-0.11 (-0.48-0.26)<br>-0.00 (-0.23-0.23)	0.8368	0.8800
rs1143623 (*IL1B*)	CC vs. GG<br>CG vs. GG	0.29 (-0.73-1.32)<br>-0.08 (-0.31-0.15)	0.6166	0.8723	0.51 (-0.45-1.47)<br>-0.06 (-0.25-0.13)	0.3746	0.7838	0.75 (-0.07-1.57)<br>-0.02 (-0.25-0.21)	0.2252	0.6531
rs1800871 (*IL10*)	TT vs. CC<br>TC vs. CC	-0.10 (-0.40-0.19)<br>0.00 (-0.16-0.17)	0.6724	0.9070	-0.10 (-0.39-0.19)<br>0.01 (-0.14-0.16)	0.6264	0.7956	-0.11 (-0.41-0.20)<br>-0.02 (-0.20-0.16)	0.7300	0.8800
rs1800872 (*IL10*)	AA vs. CC<br>AC vs. CC	-0.09 (-0.38-0.20)<br>0.00 (-0.16-0.17)	0.7430	0.9576	-0.09 (-0.39-0.20)<br>0.01 (-0.14-0.16)	0.6777	0.8032	-0.09 (-0.40-0.22)<br>-0.02 (-0.20-0.16)	0.7967	0.8800
rs1800896 (*IL10*)	GG vs. AA<br>GA vs. AA	-0.01 (-0.30-0.28)<br>-0.05 (-0.24-0.15)	0.8786	0.9944	-0.02 (-0.30-0.25)<br>-0.08 (-0.24-0.08)	0.6346	0.7956	-0.02 (-0.33-0.29)<br>-0.07 (-0.25-0.11)	0.7425	0.8800
rs2243248 (*IL4*)	GG vs. TT<br>GT vs. TT	0.03 (-0.58-0.64)<br>-0.02 (-0.23-0.20)	0.9827	0.9944	0.21 (-0.32-0.75)<br>-0.04 (-0.20-0.13)	0.6051	0.7956	0.38 (-0.25-1.00)<br>-0.07 (-0.24-0.09)	0.2706	0.6767
rs2243250 (*IL4*)	TT vs. CC<br>TC vs. CC	-0.05 (-0.48-0.38)<br>-0.07 (-0.45-0.31)	0.9237	0.9944	-0.02 (-0.41-0.37)<br>-0.07 (-0.41-0.27)	0.8825	0.9092	0.01 (-0.38-0.39)<br>-0.05 (-0.42-0.31)	0.8732	0.8800
rs2243270 (*IL4*)	GG vs. AA<br>GA vs. AA	-0.22 (-0.61-0.18)<br>-0.28 (-0.52--0.04)	0.3534	0.7950	-0.14 (-0.52-0.24)<br>-0.25 (-0.47--0.02)	0.3492	0.7656	-0.12 (-0.52-0.29)<br>-0.21 (-0.48-0.06)	0.4866	0.7354
rs1801275 (*IL4R*)	GG vs. AA<br>GA vs. AA	0.00 (-0.19-0.19)<br>0.10 (-0.10-0.30)	0.2791	0.7950	-0.01 (-0.23-0.21)<br>0.14 (-0.11-0.38)	0.1427	0.7656	-0.06 (-0.35-0.23)<br>0.13 (-0.13-0.39)	0.1170	0.6484
rs1805015 (*IL4R*)	CC vs. TT<br>CT vs. TT	-0.04 (-0.27-0.19)<br>-0.09 (-0.30-0.13)	0.6345	0.8762	-0.00 (-0.23-0.22)<br>-0.03 (-0.25-0.18)	0.9092	0.9092	-0.08 (-0.32-0.16)<br>-0.06 (-0.27-0.15)	0.7564	0.8800
rs5918 (*ITGB3*)	CC vs. TT<br>CT vs. TT	-0.04 (-0.72-0.63)<br>0.18 (-0.04-0.40)	0.2025	0.7950	-0.07 (-0.55-0.41)<br>0.10 (-0.13-0.33)	0.4689	0.7956	0.15 (-0.42-0.73)<br>0.16 (-0.06-0.39)	0.2122	0.6484
rs11003125 (*MBL2*)	GG vs. CC<br>GC vs. CC	0.25 (-0.67-1.17)<br>-0.26 (-0.48--0.04)	0.1245	0.7950	0.10 (-0.70-0.89)<br>-0.19 (-0.37--0.01)	0.2319	0.7656	0.16 (-0.68-0.99)<br>-0.22 (-0.45-0.01)	0.2099	0.6484
rs1800450 (*MBL2*)	AA vs. GG<br>AG vs. GG	0.72 (-0.22-1.67)<br>0.26 (-0.25-0.76)	0.2751	0.7950	0.79 (0.25-1.33)<br>0.28 (-0.14-0.70)	0.1281	0.7656	0.85 (0.31-1.38)<br>0.27 (-0.20-0.74)	0.1610	0.6484
rs1800451 (*MBL2*)	AA vs. GG<br>AG vs. GG	-0.28 (-0.75-0.18)<br>0.15 (-0.10-0.40)	0.1797	0.7950	-0.22 (-0.63-0.20)<br>0.11 (-0.09-0.31)	0.2498	0.7656	-0.21 (-0.63-0.20)<br>0.08 (-0.13-0.29)	0.3534	0.7234
rs5030737 (*MBL2*)	TT vs. CC<br>TC vs. CC	 -0.24 (-0.74-0.27)	0.3429	0.7950	 -0.12 (-0.74-0.50)	0.6905	0.8032	 -0.25 (-1.03-0.52)	0.5072	0.7354
rs7096206 (*MBL2*)	CC vs. GG<br>CG vs. GG	0.19 (-0.22-0.59)<br>-0.18 (-0.41-0.04)	0.1203	0.7950	0.03 (-0.45-0.50)<br>-0.16 (-0.33-0.01)	0.1691	0.7656	0.15 (-0.38-0.68)<br>-0.19 (-0.36--0.03)	0.0727	0.6484
rs1800482 (*NOS2A*)	CC vs. GG<br>CG vs. GG	1.35 (-0.08-2.78)<br>0.04 (-0.17-0.25)	0.0973	0.7950	1.33 (-0.16-2.83)<br>0.02 (-0.19-0.23)	0.1188	0.7656	1.37 (-0.17-2.91)<br>0.04 (-0.17-0.24)	0.1129	0.6484
rs9282799 (*NOS2A*)	TT vs. CC<br>TC vs. CC	-1.80 (-2.84--0.77)<br>-0.07 (-0.43-0.29)	0.2086	0.7950	-1.38 (-2.58--0.17)<br>0.08 (-0.29-0.45)	0.3369	0.7656	-1.32 (-2.50--0.14)<br>0.08 (-0.28-0.45)	0.3268	0.7234
rs1799983 (*NOS3*)	TT vs. GG<br>TG vs. GG	-0.28 (-1.16-0.59)<br>0.03 (-0.22-0.28)	0.7782	0.9647	-0.55 (-1.12-0.03)<br>0.03 (-0.18-0.23)	0.3040	0.7656	-0.78 (-1.32--0.23)<br>0.01 (-0.19-0.22)	0.1245	0.6484
rs2070744 (*NOS3*)	CC vs. TT<br>CT vs. TT	-0.17 (-0.89-0.54)<br>0.05 (-0.16-0.27)	0.7080	0.9333	-0.25 (-0.86-0.35)<br>0.05 (-0.12-0.22)	0.5330	0.7956	-0.45 (-0.87--0.03)<br>0.03 (-0.13-0.19)	0.1920	0.6484
rs662 (*PON1*)	GG vs. AA<br>GA vs. AA	-0.05 (-0.33-0.22)<br>0.04 (-0.21-0.28)	0.5488	0.8723	0.01 (-0.25-0.26)<br>0.05 (-0.15-0.24)	0.8202	0.8908	-0.03 (-0.27-0.22)<br>0.07 (-0.12-0.25)	0.4811	0.7354
rs854560 (*PON1*)	AA vs. TT<br>AT vs. TT	0.08 (-0.62-0.77)<br>-0.01 (-0.23-0.21)	0.9577	0.9944	0.06 (-0.52-0.65)<br>-0.09 (-0.29-0.11)	0.6421	0.7956	-0.10 (-0.60-0.40)<br>-0.12 (-0.32-0.08)	0.4140	0.7354
rs1801282 (*PPARG*)	GG vs. CC<br>GC vs. CC									

Variant	Genotype	Crude Model			Age-Sex Adjusted Model			Fully Adjusted[b] Model		
		β coefficient (95% CI)	P-value	FDR-adjusted P-value	β coefficient (95% CI)	P-value	FDR-adjusted P-value	β coefficient (95% CI)	P-value	FDR-adjusted P-value
rs1799762 (*SERPINE1*)	4G4G vs. 5G5G<br>4G5G vs. 5G5G	0.17 (-0.22-0.56)<br>-0.04 (-0.22-0.14)	0.5126	0.8723	0.19 (-0.22-0.61)<br>-0.07 (-0.25-0.11)	0.3917	0.7838	0.21 (-0.22-0.64)<br>-0.10 (-0.27-0.08)	0.2709	0.6767
rs1800468 (*TGFB1/B9D2*)	AA vs. GG<br>AG vs. GG									
rs1800469 (*TGFB1/B9D2*)	TT vs. CC<br>TC vs. CC	0.04 (-0.32-0.39)<br>0.20 (0.00-0.41)	0.1034	0.7950	-0.01 (-0.38-0.36)<br>0.20 (0.00-0.40)	0.1033	0.7656	0.11 (-0.25-0.48)<br>0.21 (0.00-0.42)	0.0937	0.6484
rs1800470 (*TGFB1*)	CC vs. TT<br>CT vs. TT	0.18 (-0.09-0.45)<br>0.04 (-0.19-0.27)	0.2897	0.7950	0.18 (-0.09-0.45)<br>0.04 (-0.18-0.27)	0.2905	0.7656	0.27 (-0.03-0.56)<br>0.07 (-0.19-0.32)	0.1491	0.6484
rs4986790 (*TLR4*)	GG vs. AA<br>GA vs. AA	0.16 (-1.02-1.34)<br>0.00 (-0.34-0.35)	0.9653	0.9944	0.60 (-0.57-1.77)<br>-0.02 (-0.31-0.27)	0.5875	0.7956	0.57 (-0.58-1.72)<br>-0.04 (-0.35-0.27)	0.5877	0.7747
rs1800629 (*TNF*)	AA vs. GG<br>AG vs. GG	-0.24 (-0.64-0.16)<br>-0.07 (-0.32-0.17)	0.5096	0.8723	-0.18 (-0.64-0.28)<br>-0.05 (-0.27-0.17)	0.6743	0.8032	-0.15 (-0.68-0.38)<br>0.00 (-0.19-0.20)	0.8523	0.8800
rs1800750 (*TNF*)	AA vs. GG<br>AG vs. GG	-1.13 (-3.59-1.33)<br>0.04 (-0.27-0.36)	0.5631	0.8723	-0.87 (-3.35-1.61)<br>0.11 (-0.19-0.42)	0.5641	0.7956	-0.82 (-3.40-1.76)<br>0.09 (-0.23-0.41)	0.6533	0.8420
rs361525 (*TNF*)	AA vs. GG<br>AG vs. GG	-0.51 (-0.97--0.05)<br>-0.09 (-0.37-0.20)	0.4217	0.7950	-0.69 (-1.92-0.53)<br>-0.03 (-0.34-0.27)	0.6170	0.7956	-0.71 (-1.99-0.57)<br>-0.06 (-0.37-0.24)	0.5615	0.7574
rs2239185 (*VDR*)	CC vs. TT<br>CT vs. TT	0.04 (-0.20-0.27)<br>-0.03 (-0.27-0.22)	0.8361	0.9897	-0.00 (-0.24-0.23)<br>0.03 (-0.20-0.26)	0.8945	0.9092	0.04 (-0.17-0.25)<br>0.12 (-0.11-0.36)	0.3841	0.7354
rs731236 (*VDR*)	CC vs. TT<br>CT vs. TT	-0.02 (-0.26-0.23)<br>0.10 (-0.08-0.28)	0.3608	0.7950	-0.05 (-0.30-0.20)<br>0.10 (-0.07-0.27)	0.3403	0.7656	-0.02 (-0.30-0.25)<br>0.09 (-0.10-0.28)	0.5055	0.7354
rs890945 (Chr 5q33.3)	AA vs. TT<br>AT vs. TT	-0.39 (-0.68--0.10)<br>-0.03 (-0.27-0.21)	0.1233	0.7950	-0.36 (-0.60--0.12)<br>-0.04 (-0.25-0.17)	0.1007	0.7656	-0.36 (-0.60--0.12)<br>-0.04 (-0.23-0.16)	0.0682	0.6484
Mexican Americans										
rs1042713 (*ADRB2*)	AA vs. GG<br>AG vs. GG	0.02 (-0.28-0.33)<br>-0.04 (-0.33-0.25)	0.8149	0.9914	0.05 (-0.22-0.32)<br>-0.02 (-0.32-0.28)	0.7944	0.9958	0.02 (-0.21-0.25)<br>-0.06 (-0.38-0.25)	0.7012	0.9132
rs1042714 (*ADRB2*)	GG vs. CC<br>GC vs. CC	-0.11 (-0.53-0.32)<br>0.01 (-0.13-0.16)	0.8216	0.9914	-0.12 (-0.51-0.28)<br>0.00 (-0.12-0.12)	0.7451	0.9841	-0.05 (-0.49-0.39)<br>0.02 (-0.09-0.14)	0.8641	0.9744
rs429358 (*APOE*)	CC vs. TT<br>CT vs. TT	-0.32 (-0.76-0.12)<br>0.17 (-0.06-0.40)	0.1130	0.7031	-0.34 (-0.77-0.09)<br>0.15 (-0.07-0.37)	0.1292	0.9710	-0.32 (-0.82-0.19)<br>0.10 (-0.12-0.32)	0.2912	0.9132
rs7412 (*APOE*)	TT vs. CC<br>TC vs. CC									
rs769214 (*CAT*)	GG vs. AA<br>GA vs. AA	0.03 (-0.16-0.22)<br>-0.04 (-0.17-0.09)	0.5524	0.9667	0.03 (-0.13-0.20)<br>-0.01 (-0.14-0.11)	0.7515	0.9841	0.03 (-0.12-0.18)<br>-0.02 (-0.16-0.11)	0.6808	0.9132
rs2280788 (*CCL5*)	GG vs. CC<br>GC vs. CC	0.09 (-0.64-0.83)	0.7940	0.9914	0.05 (-0.67-0.77)	0.8931	0.9958	0.05 (-0.71-0.81)	0.8913	0.9744
rs1799864 (*CCR2*)	AA vs. GG<br>AG vs. GG	0.05 (-0.53-0.63)<br>-0.12 (-0.28-0.03)	0.3907	0.9268	0.10 (-0.47-0.67)<br>-0.10 (-0.25-0.06)	0.4631	0.9804	0.10 (-0.49-0.69)<br>-0.09 (-0.26-0.07)	0.4938	0.9132
rs1205 (*CRP*)	AA vs. GG<br>AG vs. GG	0.01 (-0.23-0.25)<br>-0.01 (-0.18-0.17)	0.9875	0.9914	-0.01 (-0.25-0.22)<br>-0.03 (-0.20-0.14)	0.9182	0.9958	-0.03 (-0.27-0.22)<br>-0.04 (-0.22-0.13)	0.8736	0.9744
rs1417938 (*CRP*)	TT vs. AA<br>TA vs. AA	0.01 (-0.32-0.35)<br>-0.00 (-0.17-0.17)	0.9914	0.9914	-0.01 (-0.32-0.30)<br>-0.02 (-0.16-0.13)	0.9777	0.9958	0.03 (-0.29-0.35)<br>0.03 (-0.11-0.17)	0.9201	0.9744
rs1800947 (*CRP*)	CC vs. GG<br>CG vs. GG	-0.03 (-0.65-0.59)	0.9155	0.9914	-0.25 (-0.92-0.43)	0.4570	0.9804	-0.35 (-0.77-0.08)	0.1040	0.9040
rs2808630 (*CRP*)	GG vs. AA<br>GA vs. AA	0.13 (-0.21-0.47)<br>-0.18 (-0.40-0.03)	0.1103	0.7031	0.12 (-0.21-0.44)<br>-0.13 (-0.33-0.07)	0.2436	0.9710	0.08 (-0.20-0.36)<br>-0.09 (-0.28-0.11)	0.4506	0.9132
rs3091244 (*CRP*)	AA vs. CC<br>AC vs. CC<br>AT vs. CC<br>CT vs. CC<br>TT vs. CC	-0.31 (-1.56-0.94)<br>0.14 (-0.19-0.47)<br>-0.23 (-0.78-0.33)<br>0.03 (-0.15-0.21)<br>0.05 (-0.29-0.40)	0.7432	0.9914	-0.27 (-1.35-0.81)<br>0.16 (-0.14-0.46)<br>-0.08 (-0.64-0.48)<br>0.02 (-0.14-0.18)<br>0.01 (-0.31-0.32)	0.8225	0.9958	-0.31 (-1.29-0.67)<br>0.21 (-0.10-0.51)<br>-0.05 (-0.57-0.47)<br>0.07 (-0.09-0.24)<br>0.05 (-0.27-0.36)	0.6880	0.9132
rs3093058 (*CRP*)	TT vs. AA<br>TA vs. AA									
rs3093066 (*CRP*)	AA vs. CC<br>AC vs. CC	-0.31 (-0.72-0.10)	0.1298	0.7269	-0.34 (-0.77-0.09)	0.1143	0.9710	-0.42 (-0.88-0.05)	0.0748	0.9040
rs11265260 (*CRP*)	GG vs. AA<br>GA vs. AA	-0.42 (-1.83-0.98)<br>-0.13 (-0.41-0.14)	0.4859	0.9383	-0.26 (-1.40-0.87)<br>-0.04 (-0.27-0.20)	0.8247	0.9958	-0.34 (-1.43-0.74)<br>-0.00 (-0.23-0.23)	0.8041	0.9744
rs12093699 (*CRP*)	AA vs. GG<br>AG vs. GG	0.02 (-0.27-0.31)<br>0.03 (-0.12-0.19)	0.9233	0.9914	0.02 (-0.24-0.28)<br>0.00 (-0.16-0.16)	0.9776	0.9958	0.02 (-0.24-0.27)<br>0.02 (-0.13-0.17)	0.9714	0.9822
rs12744244 (*CRP*)	AA vs. CC<br>AC vs. CC	-0.06 (-1.18-1.06)<br>0.02 (-0.25-0.29)	0.9803	0.9914	0.01 (-0.97-0.98)<br>0.00 (-0.26-0.26)	0.9993	0.9993	0.02 (-0.99-1.03)<br>-0.02 (-0.28-0.24)	0.9822	0.9822
rs2027471 (*CRP*)	AA vs. TT<br>AT vs. TT	-0.02 (-0.28-0.24)<br>-0.03 (-0.23-0.16)	0.9362	0.9914	-0.04 (-0.30-0.23)<br>-0.08 (-0.25-0.10)	0.6914	0.9804	-0.07 (-0.35-0.22)<br>-0.09 (-0.25-0.07)	0.5916	0.9132
rs2592887 (*CRP*)	AA vs. GG<br>AG vs. GG	0.05 (-0.20-0.30)<br>-0.02 (-0.26-0.22)	0.8599	0.9914	0.03 (-0.21-0.27)<br>-0.07 (-0.29-0.16)	0.6782	0.9804	0.03 (-0.24-0.29)<br>-0.08 (-0.29-0.13)	0.6011	0.9132
rs2794520 (*CRP*)	AA vs. GG<br>AG vs. GG	0.03 (-0.20-0.27)<br>-0.06 (-0.27-0.14)	0.6749	0.9914	0.01 (-0.23-0.26)<br>-0.09 (-0.28-0.10)	0.5390	0.9804	0.00 (-0.25-0.25)<br>-0.09 (-0.27-0.10)	0.5646	0.9132
rs3093075 (*CRP*)	AA vs. CC<br>AC vs. CC	-2.07 (-2.72--1.42)<br>-0.01 (-0.27-0.25)	**0.0214**	0.5992	-1.77 (-2.36--1.18)<br>0.04 (-0.22-0.30)	**0.0404**	0.9710	-1.82 (-2.48--1.16)<br>0.05 (-0.20-0.29)	**0.0243**	0.9040
rs1799963 (*F2*)	AG vs. GG	0.03 (-0.77-0.83)	0.9361	0.9914	-0.05 (-0.90-0.81)	0.9130	0.9958	-0.04 (-1.01-0.94)	0.9380	0.9744
rs6025 (*F5*)	AA vs. GG<br>AG vs. GG									
rs1801274 (*FCGR2A*)	AA vs. GG<br>AG vs. GG	0.14 (-0.10-0.38)<br>0.03 (-0.17-0.23)	0.3002	0.9268	0.10 (-0.11-0.31)<br>-0.03 (-0.20-0.14)	0.2293	0.9710	0.07 (-0.15-0.30)<br>-0.04 (-0.25-0.17)	0.3925	0.9132
rs1800790 (*FGB*)	AA vs. GG<br>AG vs. GG	-0.09 (-0.76-0.59)<br>-0.01 (-0.19-0.16)	0.9457	0.9914	0.07 (-0.61-0.74)<br>0.03 (-0.13-0.19)	0.9241	0.9958	0.09 (-0.58-0.76)<br>0.05 (-0.11-0.22)	0.8157	0.9744

Variant	Genotype	Crude Model			Age-Sex Adjusted Model			Fully Adjusted[b] Model		
		β coefficient (95% CI)	P-value	FDR-adjusted P-value	β coefficient (95% CI)	P-value	FDR-adjusted P-value	β coefficient (95% CI)	P-value	FDR-adjusted P-value
rs1260326 (*GCKR*)	TT vs. CC<br>TC vs. CC	0.08 (-0.39-0.55)<br>-0.11 (-0.26-0.05)	0.4477	0.9268	0.05 (-0.39-0.50)<br>-0.10 (-0.24-0.04)	0.5146	0.9804	0.08 (-0.36-0.53)<br>-0.05 (-0.18-0.09)	0.6509	0.9132
rs1143623 (*IL1B*)	CC vs. GG<br>CG vs. GG	0.06 (-0.18-0.30)<br>0.09 (-0.07-0.26)	0.5840	0.9709	0.16 (-0.06-0.39)<br>0.13 (-0.04-0.29)	0.2100	0.9710	0.19 (-0.04-0.43)<br>0.10 (-0.09-0.30)	0.2009	0.9040
rs1800871 (*IL10*)	TT vs. CC<br>TC vs. CC	0.13 (-0.12-0.38)<br>0.02 (-0.15-0.19)	0.5315	0.9601	0.11 (-0.14-0.37)<br>-0.01 (-0.18-0.15)	0.5153	0.9804	0.09 (-0.17-0.36)<br>-0.04 (-0.21-0.13)	0.5088	0.9132
rs1800872 (*IL10*)	AA vs. CC<br>AC vs. CC	0.13 (-0.13-0.39)<br>0.04 (-0.15-0.22)	0.5895	0.9709	0.11 (-0.15-0.37)<br>0.01 (-0.18-0.19)	0.6158	0.9804	0.09 (-0.18-0.36)<br>-0.02 (-0.20-0.16)	0.6421	0.9132
rs1800896 (*IL10*)	GG vs. AA<br>GA vs. AA	-0.26 (-0.73-0.20)<br>-0.13 (-0.37-0.10)	0.3885	0.9268	-0.29 (-0.70-0.12)<br>-0.11 (-0.36-0.14)	0.3470	0.9710	-0.29 (-0.70-0.12)<br>-0.14 (-0.38-0.10)	0.2952	0.9132
rs2243248 (*IL4*)	GG vs. TT<br>GT vs. TT	-0.14 (-0.74-0.47)<br>-0.13 (-0.30-0.05)	0.3894	0.9268	-0.23 (-0.75-0.29)<br>-0.03 (-0.23-0.18)	0.6791	0.9804	-0.24 (-0.78-0.29)<br>-0.03 (-0.22-0.16)	0.6196	0.9132
rs2243250 (*IL4*)	TT vs. CC<br>TC vs. CC	-0.03 (-0.35-0.29)<br>-0.05 (-0.31-0.21)	0.9061	0.9914	0.00 (-0.32-0.33)<br>-0.02 (-0.25-0.21)	0.9760	0.9958	-0.03 (-0.36-0.29)<br>-0.06 (-0.28-0.15)	0.8577	0.9744
rs2243270 (*IL4*)	GG vs. AA<br>GA vs. AA	-0.06 (-0.41-0.29)<br>-0.08 (-0.30-0.15)	0.7764	0.9914	-0.03 (-0.38-0.32)<br>-0.06 (-0.24-0.13)	0.8451	0.9958	-0.06 (-0.40-0.29)<br>-0.07 (-0.26-0.11)	0.7632	0.9713
rs1801275 (*IL4R*)	GG vs. AA<br>GA vs. AA	0.19 (-0.16-0.54)<br>-0.02 (-0.20-0.16)	0.4139	0.9268	0.26 (-0.17-0.70)<br>0.02 (-0.16-0.19)	0.3105	0.9710	0.29 (-0.16-0.74)<br>0.02 (-0.18-0.22)	0.3119	0.9132
rs1805015 (*IL4R*)	CC vs. TT<br>CT vs. TT	0.86 (0.02-1.71)<br>-0.02 (-0.29-0.25)	0.1025	0.7031	0.91 (-0.04-1.86)<br>-0.01 (-0.26-0.25)	0.1104	0.9710	1.16 (0.09-2.22)<br>0.02 (-0.22-0.25)	0.0507	0.9040
rs5918 (*ITGB3*)	CC vs. TT<br>CT vs. TT	0.46 (-0.10-1.03)<br>0.02 (-0.26-0.30)	0.4065	0.9268	0.55 (0.00-1.10)<br>0.02 (-0.27-0.32)	0.3211	0.9710	0.67 (0.09-1.25)<br>-0.03 (-0.28-0.22)	0.1697	0.9040
rs11003125 (*MBL2*)	GG vs. CC<br>GC vs. CC	-0.18 (-0.47-0.11)<br>-0.11 (-0.39-0.17)	0.3300	0.9268	-0.12 (-0.40-0.15)<br>-0.08 (-0.33-0.16)	0.5089	0.9804	-0.09 (-0.36-0.18)<br>-0.12 (-0.35-0.12)	0.4734	0.9132
rs1800450 (*MBL2*)	AA vs. GG<br>AG vs. GG	-0.05 (-0.38-0.27)<br>0.12 (-0.10-0.33)	0.3616	0.9268	-0.07 (-0.32-0.18)<br>0.08 (-0.14-0.29)	0.5181	0.9804	-0.05 (-0.31-0.20)<br>0.12 (-0.11-0.35)	0.3288	0.9132
rs1800451 (*MBL2*)	AA vs. GG<br>AG vs. GG	-0.08 (-0.44-0.27)	0.6314	0.9914	-0.14 (-0.55-0.27)	0.4978	0.9804	-0.14 (-0.62-0.33)	0.5342	0.9132
rs5030737 (*MBL2*)	TT vs. CC<br>TC vs. CC	0.08 (-0.49-0.65)<br>0.37 (-0.00-0.75)	0.0534	0.7031	-0.13 (-1.02-0.77)<br>0.41 (-0.01-0.83)	0.0613	0.9710	-0.04 (-0.92-0.85)<br>0.38 (-0.07-0.82)	0.1084	0.9040
rs7096206 (*MBL2*)	CC vs. GG<br>CG vs. GG	0.25 (-0.51-1.01)<br>0.17 (-0.07-0.42)	0.3269	0.9268	0.23 (-0.59-1.05)<br>0.12 (-0.15-0.40)	0.5801	0.9804	-0.02 (-0.63-0.60)<br>0.10 (-0.20-0.40)	0.7001	0.9132
rs1800482 (*NOS2A*)	CC vs. GG<br>CG vs. GG									
rs9282799 (*NOS2A*)	TT vs. CC<br>TC vs. CC	0.67 (0.29-1.05)	**0.0014**	0.0784	0.59 (-0.24-1.42)	0.1567	0.9710	0.58 (-0.44-1.59)	0.2513	0.9132
rs1799983 (*NOS3*)	TT vs. GG<br>TG vs. GG	-0.05 (-0.49-0.39)<br>-0.19 (-0.43-0.05)	0.2171	0.9268	-0.18 (-0.57-0.22)<br>-0.21 (-0.41--0.00)	0.0844	0.9710	-0.18 (-0.56-0.19)<br>-0.24 (-0.43--0.05)	**0.0326**	0.9040
rs2070744 (*NOS3*)	CC vs. TT<br>CT vs. TT	-0.07 (-0.58-0.44)<br>-0.08 (-0.29-0.12)	0.7415	0.9914	-0.14 (-0.68-0.39)<br>-0.10 (-0.32-0.11)	0.6168	0.9804	-0.15 (-0.69-0.39)<br>-0.13 (-0.35-0.08)	0.5072	0.9132
rs662 (*PON1*)	GG vs. AA<br>GA vs. AA	-0.17 (-0.47-0.13)<br>-0.10 (-0.31-0.12)	0.4165	0.9268	-0.14 (-0.40-0.12)<br>-0.04 (-0.23-0.15)	0.4477	0.9804	-0.10 (-0.35-0.14)<br>-0.08 (-0.27-0.11)	0.5639	0.9132
rs854560 (*PON1*)	AA vs. TT<br>AT vs. TT	-0.03 (-0.71-0.64)<br>-0.05 (-0.28-0.18)	0.8655	0.9914	-0.09 (-0.77-0.59)<br>-0.08 (-0.28-0.11)	0.7149	0.9830	-0.00 (-0.66-0.66)<br>-0.12 (-0.33-0.08)	0.5636	0.9132
rs1801282 (*PPARG*)	GG vs. CC<br>GC vs. CC	0.08 (-0.60-0.76)<br>-0.16 (-0.39-0.07)	0.2628	0.9268	0.19 (-0.60-0.98)<br>-0.16 (-0.38-0.07)	0.2866	0.9710	0.26 (-0.42-0.95)<br>-0.15 (-0.37-0.07)	0.2260	0.9040
rs1799762 (*SERPINE1*)	4G4G vs. 5G5G<br>4G5G vs. 5G5G	0.25 (0.05-0.46)<br>0.05 (-0.11-0.20)	0.1069	0.7031	0.19 (-0.02-0.40)<br>-0.02 (-0.17-0.14)	0.1934	0.9710	0.13 (-0.06-0.32)<br>-0.06 (-0.21-0.09)	0.2102	0.9040
rs1800468 (*TGFB1/B9D2*)	AA vs. GG<br>AG vs. GG	-0.76 (-1.83-0.30)<br>-0.08 (-0.44-0.28)	0.4634	0.9268	-0.70 (-1.53-0.13)<br>-0.15 (-0.49-0.18)	0.2847	0.9710	-0.65 (-1.53-0.23)<br>-0.13 (-0.48-0.22)	0.3664	0.9132
rs1800469 (*TGFB1/B9D2*)	TT vs. CC<br>TC vs. CC	-0.01 (-0.30-0.28)<br>0.07 (-0.15-0.28)	0.7835	0.9914	0.01 (-0.26-0.28)<br>0.05 (-0.19-0.28)	0.9006	0.9958	0.08 (-0.17-0.34)<br>0.04 (-0.21-0.29)	0.8251	0.9744
rs1800470 (*TGFB1*)	CC vs. TT<br>CT vs. TT	0.08 (-0.20-0.35)<br>0.01 (-0.20-0.22)	0.7552	0.9914	0.09 (-0.20-0.38)<br>-0.00 (-0.24-0.23)	0.6799	0.9804	0.16 (-0.15-0.48)<br>0.02 (-0.20-0.24)	0.3660	0.9132
rs4986790 (*TLR4*)	GG vs. AA<br>GA vs. AA	0.23 (-0.21-0.68)<br>-0.26 (-0.58-0.06)	0.1025	0.7031	-0.10 (-1.23-1.02)<br>-0.28 (-0.62-0.05)	0.1074	0.9710	0.01 (-1.22-1.24)<br>-0.31 (-0.71-0.09)	0.1369	0.9040
rs1800629 (*TNF*)	AA vs. GG<br>AG vs. GG	0.14 (-0.84-1.13)<br>0.08 (-0.19-0.36)	0.7384	0.9914	-0.34 (-1.07-0.38)<br>0.03 (-0.23-0.30)	0.6952	0.9804	0.03 (-0.43-0.50)<br>0.02 (-0.25-0.28)	0.9396	0.9744
rs1800750 (*TNF*)	AA vs. GG<br>AG vs. GG	0.27 (-0.19-0.74)	0.2328	0.9268	0.21 (-0.25-0.68)	0.3531	0.9710	0.28 (-0.11-0.66)	0.1522	0.9040
rs361525 (*TNF*)	AA vs. GG<br>AG vs. GG	1.42 (-0.89-3.73)<br>-0.01 (-0.32-0.30)	0.4025	0.9268	1.27 (-0.44-2.97)<br>-0.01 (-0.33-0.31)	0.3956	0.9804	1.21 (-0.66-3.07)<br>-0.01 (-0.28-0.27)	0.3804	0.9132
rs2239185 (*VDR*)	CC vs. TT<br>CT vs. TT	-0.19 (-0.38-0.00)<br>0.01 (-0.20-0.23)	0.1569	0.7988	-0.17 (-0.37-0.03)<br>0.02 (-0.19-0.23)	0.1862	0.9710	-0.19 (-0.41-0.03)<br>-0.02 (-0.23-0.20)	0.2193	0.9040
rs731236 (*VDR*)	CC vs. TT<br>CT vs. TT	-0.18 (-0.57-0.22)<br>0.07 (-0.16-0.30)	0.5059	0.9443	-0.20 (-0.54-0.15)<br>0.09 (-0.13-0.32)	0.3399	0.9710	-0.14 (-0.49-0.21)<br>0.07 (-0.18-0.33)	0.5613	0.9132
rs890945 (Chr 5q33.3)	AA vs. TT<br>AT vs. TT	-0.26 (-0.66-0.14)<br>-0.09 (-0.41-0.23)	0.4490	0.9268	-0.18 (-0.55-0.20)<br>-0.11 (-0.44-0.23)	0.5624	0.9804	-0.22 (-0.60-0.16)<br>-0.09 (-0.44-0.26)	0.5382	0.9132

CI, confidence interval; FDR, false-discovery rate. Variants with missing results had unstable statistical models.

a) Defined as log-transformed albumin-to-creatinine ratio (ACR) and analyzed as a continuous variable. b) Analyses adjusted for age, sex, alcohol consumption, educational attainment, and waist:hip ratio.

Table S3. Complete results of associations of candidate gene polymorphisms and sex-specific albuminuria[a], additive genetic model

Variant	Crude Model			Age-Adjusted Model			Fully Adjusted[b] Model		
	OR (95% CI)	P-value	FDR-adjusted P-value	OR (95% CI)	P-value	FDR-adjusted P-value	OR (95% CI)	P-value	FDR-adjusted P-value
Non-Hispanic whites									
rs1042713 (*ADRB2*)	0.97 (0.74-1.27)	0.7939	0.9475	0.99 (0.77-1.27)	0.9292	0.9452	0.99 (0.74-1.32)	0.9436	0.9907
rs1042714 (*ADRB2*)	0.99 (0.80-1.24)	0.9424	0.9907	0.98 (0.78-1.24)	0.8868	0.9231	0.96 (0.74-1.25)	0.7542	0.9818
rs429358 (*APOE*)	0.86 (0.62-1.19)	0.3410	0.7904	0.92 (0.66-1.29)	0.6172	0.9231	0.93 (0.66-1.32)	0.6858	0.9818
rs7412 (*APOE*)	1.23 (0.93-1.64)	0.1374	0.7904	1.15 (0.88-1.52)	0.2924	0.9231	1.13 (0.81-1.57)	0.4544	0.9818
rs769214 (*CAT*)	0.98 (0.74-1.28)	0.8633	0.9475	0.97 (0.75-1.26)	0.8219	0.9231	0.98 (0.74-1.29)	0.8778	0.9818
rs2280788 (*CCL5*)	1.04 (0.67-1.61)	0.8685	0.9475	1.22 (0.74-2.03)	0.4213	0.9231	1.19 (0.66-2.15)	0.5578	0.9818
rs1799864 (*CCR2*)	1.11 (0.79-1.56)	0.5427	0.9303	1.14 (0.78-1.65)	0.4923	0.9231	1.17 (0.84-1.64)	0.3370	0.9818
rs1205 (*CRP*)	0.91 (0.78-1.07)	0.2422	0.7904	0.95 (0.80-1.13)	0.5532	0.9231	0.98 (0.81-1.20)	0.8644	0.9818
rs1417938 (*CRP*)	1.09 (0.80-1.48)	0.5813	0.9475	1.04 (0.78-1.39)	0.7840	0.9231	0.95 (0.69-1.31)	0.7594	0.9818
rs1800947 (*CRP*)	1.09 (0.53-2.26)	0.8090	0.9475	1.08 (0.57-2.03)	0.8050	0.9231	1.06 (0.61-1.87)	0.8194	0.9818
rs2808630 (*CRP*)	0.94 (0.67-1.32)	0.7148	0.9475	0.96 (0.72-1.30)	0.8031	0.9231	1.02 (0.75-1.39)	0.8798	0.9818
rs3091244 (*CRP*)[c]	1.11 (0.72-1.73)<br>1.12 (0.83-1.52)	0.6243	0.9475	1.05 (0.66-1.66)<br>1.08 (0.81-1.44)	0.8326	0.9231	1.02 (0.58-1.79)<br>1.01 (0.73-1.38)	0.9971	0.9971
rs3093058 (*CRP*)	2.56 (0.41-15.87)	0.2974	0.7904	2.48 (0.50-12.39)	0.2540	0.9231	2.30 (0.39-13.58)	0.3422	0.9818
rs3093066 (*CRP*)	1.17 (0.40-3.42)	0.7593	0.9475	1.17 (0.39-3.49)	0.7680	0.9231	1.64 (0.65-4.11)	0.2804	0.9818
rs11265260 (*CRP*)	1.01 (0.68-1.51)	0.9577	0.9907	0.93 (0.60-1.45)	0.7394	0.9231	0.90 (0.54-1.50)	0.6711	0.9818
rs12093699 (*CRP*)	1.13 (0.82-1.55)	0.4306	0.7904	1.09 (0.80-1.48)	0.5619	0.9231	1.04 (0.75-1.43)	0.8041	0.9818
rs12744244 (*CRP*)	1.18 (0.84-1.65)	0.3240	0.7904	1.14 (0.81-1.60)	0.4362	0.9231	1.09 (0.76-1.56)	0.6309	0.9818
rs2027471 (*CRP*)	0.88 (0.76-1.03)	0.1000	0.7904	0.93 (0.78-1.10)	0.3712	0.9231	0.97 (0.79-1.18)	0.7433	0.9818
rs2592887 (*CRP*)	0.91 (0.77-1.09)	0.2853	0.7904	0.93 (0.78-1.11)	0.4098	0.9231	0.95 (0.79-1.15)	0.5943	0.9818
rs2794520 (*CRP*)	0.93 (0.80-1.09)	0.3704	0.7904	0.98 (0.82-1.17)	0.8215	0.9231	1.01 (0.83-1.22)	0.9442	0.9907
rs3093075 (*CRP*)	1.04 (0.70-1.54)	0.8475	0.9475	0.97 (0.63-1.49)	0.8737	0.9231	0.96 (0.57-1.64)	0.8836	0.9818
rs1799963 (*F2*)	1.88 (0.87-4.03)	0.1024	0.7904	1.57 (0.65-3.76)	0.2981	0.9231	1.79 (0.71-4.52)	0.2062	0.9818
rs6025 (*F5*)	1.26 (0.78-2.06)	0.3288	0.7904	1.09 (0.63-1.89)	0.7346	0.9231	1.02 (0.60-1.74)	0.9292	0.9907
rs1801274 (*FCGR2A*)	1.05 (0.85-1.29)	0.6705	0.9475	1.05 (0.84-1.31)	0.6480	0.9231	1.09 (0.84-1.40)	0.5085	0.9818
rs1800790 (*FGB*)	0.96 (0.78-1.20)	0.7222	0.9475	0.98 (0.79-1.21)	0.8259	0.9231	0.94 (0.73-1.23)	0.6502	0.9818
rs1260326 (*GCKR*)	1.11 (0.86-1.44)	0.4037	0.7904	1.17 (0.91-1.51)	0.2129	0.9231	1.21 (0.96-1.53)	0.1002	0.9818
rs1143623 (*IL1B*)	1.03 (0.75-1.41)	0.8469	0.9475	1.04 (0.74-1.47)	0.8073	0.9231	1.07 (0.79-1.45)	0.6397	0.9818
rs1800871 (*IL10*)	1.00 (0.82-1.21)	0.9835	0.9916	1.01 (0.84-1.22)	0.8918	0.9231	1.02 (0.82-1.27)	0.8542	0.9818
rs1800872 (*IL10*)	0.97 (0.79-1.18)	0.7394	0.9475	0.99 (0.81-1.19)	0.8760	0.9231	1.01 (0.81-1.24)	0.9577	0.9907
rs1800896 (*IL10*)	0.95 (0.75-1.21)	0.6694	0.9475	0.95 (0.74-1.21)	0.6465	0.9231	0.95 (0.75-1.22)	0.6849	0.9818
rs2243248 (*IL4*)	1.05 (0.71-1.55)	0.7982	0.9475	1.25 (0.85-1.82)	0.2409	0.9231	1.15 (0.71-1.85)	0.5656	0.9818
rs2243250 (*IL4*)	0.91 (0.70-1.17)	0.4347	0.7904	0.90 (0.69-1.17)	0.3988	0.9231	0.90 (0.69-1.16)	0.4021	0.9818
rs2243270 (*IL4*)	0.90 (0.71-1.15)	0.3940	0.7904	0.89 (0.69-1.16)	0.3787	0.9231	0.90 (0.70-1.17)	0.4333	0.9818
rs1801275 (*IL4R*)	1.01 (0.76-1.34)	0.9543	0.9907	1.08 (0.79-1.48)	0.6322	0.9231	1.08 (0.75-1.54)	0.6640	0.9818
rs1805015 (*IL4R*)	1.04 (0.72-1.50)	0.8475	0.9475	1.08 (0.72-1.62)	0.6948	0.9231	1.07 (0.68-1.68)	0.7632	0.9818
rs5918 (*ITGB3*)	1.04 (0.77-1.39)	0.8012	0.9475	1.04 (0.75-1.45)	0.7876	0.9231	1.04 (0.77-1.39)	0.8077	0.9818
rs11003125 (*MBL2*)	1.24 (0.96-1.61)	0.0889	0.7904	1.21 (0.94-1.55)	0.1290	0.9231	1.22 (0.92-1.60)	0.1535	0.9818
rs1800450 (*MBL2*)	1.13 (0.87-1.45)	0.3413	0.7904	1.08 (0.83-1.42)	0.5449	0.9231	1.09 (0.82-1.45)	0.5589	0.9818
rs1800451 (*MBL2*)	1.22 (0.77-1.94)	0.3885	0.7904	1.17 (0.74-1.86)	0.4845	0.9231	1.22 (0.76-1.94)	0.3935	0.9818
rs5030737 (*MBL2*)	1.49 (0.86-2.60)	0.1491	0.7904	1.55 (0.86-2.77)	0.1370	0.9231	1.58 (0.86-2.92)	0.1350	0.9818
rs7096206 (*MBL2*)	0.97 (0.75-1.26)	0.8028	0.9475	0.97 (0.73-1.28)	0.7971	0.9231	1.03 (0.76-1.40)	0.8346	0.9818
rs1800482 (*NOS2A*)	3.86 (0.28-52.28)	0.2951	0.7904	1.82 (0.14-24.37)	0.6367	0.9231	2.04 (0.19-22.44)	0.5441	0.9818
rs9282799 (*NOS2A*)	3.76 (0.95-14.82)	0.0578	0.7904	1.77 (0.44-7.19)	0.4054	0.9231	1.74 (0.40-7.50)	0.4418	0.9818
rs1799983 (*NOS3*)	1.12 (0.87-1.43)	0.3671	0.7904	1.18 (0.93-1.51)	0.1711	0.9231	1.19 (0.94-1.51)	0.1423	0.9818
rs2070744 (*NOS3*)	0.96 (0.78-1.18)	0.6937	0.9475	1.00 (0.79-1.25)	0.9855	0.9855	1.00 (0.76-1.31)	0.9946	0.9971
rs662 (*PON1*)	1.00 (0.78-1.28)	0.9916	0.9916	1.06 (0.83-1.35)	0.6349	0.9231	1.15 (0.90-1.47)	0.2649	0.9818
rs854560 (*PON1*)	1.10 (0.90-1.34)	0.3434	0.7904	1.08 (0.89-1.31)	0.4141	0.9231	1.03 (0.85-1.24)	0.7830	0.9818
rs1801282 (*PPARG*)	1.18 (0.87-1.59)	0.2672	0.7904	1.24 (0.95-1.63)	0.1118	0.9231	1.30 (0.93-1.82)	0.1140	0.9818
rs1799762 (*SERPINE1*)	1.08 (0.89-1.31)	0.4131	0.7904	1.11 (0.89-1.38)	0.3542	0.9231	1.13 (0.91-1.41)	0.2587	0.9818
rs1800468 (*TGFB1/B9D2*)	1.18 (0.75-1.85)	0.4547	0.8024	1.28 (0.80-2.04)	0.2896	0.9231	1.32 (0.84-2.06)	0.2141	0.9818
rs1800469 (*TGFB1/B9D2*)	1.12 (0.86-1.45)	0.3784	0.7904	1.13 (0.87-1.47)	0.3598	0.9231	1.12 (0.84-1.50)	0.4315	0.9818
rs1800470 (*TGFB1*)	1.16 (0.85-1.58)	0.3237	0.7904	1.15 (0.85-1.56)	0.3361	0.9231	1.14 (0.86-1.50)	0.3513	0.9818
rs4986790 (*TLR4*)	1.14 (0.82-1.58)	0.4228	0.7904	1.14 (0.82-1.58)	0.4235	0.9231	1.11 (0.78-1.57)	0.5513	0.9818
rs1800629 (*TNF*)	0.92 (0.62-1.36)	0.6622	0.9475	0.93 (0.62-1.38)	0.6934	0.9231	0.90 (0.55-1.45)	0.6397	0.9818
rs1800750 (*TNF*)	2.38 (1.56-3.63)	**0.0003**	**0.0180**	3.07 (1.84-5.11)	**0.0001**	**0.0059**	2.74 (1.34-5.61)	**0.0079**	0.2370
rs361525 (*TNF*)	1.59 (1.07-2.36)	**0.0235**	0.7050	1.84 (1.14-2.97)	**0.0143**	0.4219	1.99 (1.23-3.25)	**0.0075**	0.2370
rs2239185 (*VDR*)	0.87 (0.70-1.07)	0.1752	0.7904	0.89 (0.72-1.10)	0.2757	0.9231	0.95 (0.78-1.17)	0.6336	0.9818
rs731236 (*VDR*)	1.27 (1.01-1.58)	**0.0404**	0.7904	1.24 (1.00-1.54)	**0.0465**	0.9145	1.16 (0.95-1.42)	0.1278	0.9818
rs890945 (Chr 5q33.3)	1.17 (0.90-1.52)	0.2401	0.7904	1.22 (0.93-1.62)	0.1493	0.9231	1.14 (0.87-1.49)	0.3181	0.9818

Variant	Crude Model			Age-Adjusted Model			Fully Adjusted[b] Model		
	OR (95% CI)	P-value	FDR-adjusted P-value	OR (95% CI)	P-value	FDR-adjusted P-value	OR (95% CI)	P-value	FDR-adjusted P-value
Non-Hispanic blacks									
rs1042713 (*ADRB2*)	1.00 (0.79-1.27)	0.9897	0.9897	1.04 (0.81-1.34)	0.7574	0.9132	1.00 (0.75-1.34)	1.0000	1.0000
rs1042714 (*ADRB2*)	1.00 (0.74-1.35)	0.9841	0.9897	0.95 (0.69-1.32)	0.7493	0.9132	0.97 (0.67-1.40)	0.8451	0.9390
rs429358 (*APOE*)	0.98 (0.74-1.29)	0.8861	0.9667	1.05 (0.80-1.37)	0.7250	0.9132	1.01 (0.79-1.30)	0.9366	0.9835
rs7412 (*APOE*)	1.40 (0.94-2.09)	0.0908	0.6582	1.35 (0.95-1.92)	0.0938	0.6818	1.46 (1.00-2.14)	0.0502	0.5280
rs769214 (*CAT*)	0.92 (0.71-1.18)	0.4759	0.8649	0.91 (0.69-1.19)	0.4645	0.8900	0.91 (0.68-1.21)	0.4852	0.8822
rs2280788 (*CCL5*)	0.31 (0.03-2.76)	0.2796	0.8649	0.41 (0.04-4.20)	0.4354	0.8900	0.42 (0.04-4.86)	0.4677	0.8769
rs1799864 (*CCR2*)	0.91 (0.69-1.20)	0.4774	0.8649	0.93 (0.69-1.25)	0.6092	0.9132	0.91 (0.68-1.21)	0.5014	0.8848
rs1205 (*CRP*)	0.81 (0.62-1.07)	0.1285	0.7009	0.81 (0.60-1.08)	0.1350	0.7060	0.79 (0.58-1.09)	0.1447	0.7235
rs1417938 (*CRP*)	0.95 (0.69-1.32)	0.7540	0.9423	1.01 (0.66-1.53)	0.9779	0.9917	1.17 (0.76-1.82)	0.4629	0.8769
rs1800947 (*CRP*)	0.68 (0.17-2.68)	0.5658	0.9386	0.50 (0.13-1.94)	0.3031	0.8900	0.57 (0.15-2.14)	0.3853	0.8769
rs2808630 (*CRP*)	1.39 (1.12-1.72)	**0.0041**	0.2460	1.33 (1.10-1.61)	**0.0049**	0.2891	1.30 (1.04-1.64)	**0.0232**	0.4515
rs3091244 (*CRP*)[c]	1.01 (0.73-1.39)<br>0.79 (0.58-1.06)	0.2463	0.8649	1.02 (0.77-1.36)<br>0.82 (0.60-1.11)	0.3312	0.8900	1.02 (0.75-1.40)<br>0.88 (0.62-1.26)	0.6909	0.9390
rs3093058 (*CRP*)	0.67 (0.49-0.91)	**0.0134**	0.4020	0.68 (0.51-0.92)	**0.0152**	0.4484	0.66 (0.48-0.89)	**0.0091**	0.4515
rs3093066 (*CRP*)	1.21 (0.87-1.68)	0.2412	0.8649	1.25 (0.92-1.71)	0.1436	0.7060	1.18 (0.83-1.69)	0.3343	0.8769
rs11265260 (*CRP*)	1.04 (0.67-1.63)	0.8445	0.9560	1.05 (0.62-1.78)	0.8396	0.9227	1.15 (0.65-2.04)	0.6205	0.9390
rs12093699 (*CRP*)	0.98 (0.79-1.21)	0.8154	0.9423	0.96 (0.77-1.20)	0.7369	0.9132	0.92 (0.70-1.21)	0.5381	0.9225
rs12744244 (*CRP*)	0.79 (0.44-1.40)	0.4010	0.8649	0.81 (0.43-1.53)	0.5047	0.8900	0.88 (0.43-1.82)	0.7260	0.9390
rs2027471 (*CRP*)	0.86 (0.65-1.14)	0.2682	0.8649	0.86 (0.63-1.16)	0.3022	0.8900	0.79 (0.56-1.12)	0.1802	0.7792
rs2592887 (*CRP*)	0.97 (0.76-1.24)	0.7900	0.9423	0.96 (0.75-1.21)	0.7048	0.9132	0.90 (0.67-1.20)	0.4548	0.8769
rs2794520 (*CRP*)	0.89 (0.67-1.17)	0.3720	0.8649	0.90 (0.67-1.20)	0.4437	0.8900	0.84 (0.60-1.18)	0.3035	0.8769
rs3093075 (*CRP*)	1.11 (0.85-1.46)	0.4297	0.8649	1.11 (0.86-1.44)	0.4146	0.8900	1.11 (0.84-1.46)	0.4563	0.8769
rs1799963 (*F2*)	0.70 (0.09-5.60)	0.7294	0.9423	0.59 (0.07-4.96)	0.6125	0.9132	0.76 (0.09-6.53)	0.7947	0.9390
rs6025 (*F5*)	0.74 (0.15-3.68)	0.6984	0.9423	0.99 (0.19-5.22)	0.9917	0.9917	1.19 (0.22-6.36)	0.8320	0.9390
rs1801274 (*FCGR2A*)	1.24 (0.89-1.73)	0.1918	0.8649	1.32 (0.94-1.85)	0.1040	0.6818	1.31 (0.93-1.86)	0.1183	0.6453
rs1800790 (*FGB*)	0.87 (0.51-1.49)	0.6076	0.9386	0.90 (0.50-1.64)	0.7246	0.9132	1.02 (0.53-1.96)	0.9507	0.9835
rs1260326 (*GCKR*)	0.93 (0.72-1.20)	0.5823	0.9386	0.88 (0.66-1.16)	0.3441	0.8900	0.83 (0.58-1.18)	0.2842	0.8769
rs1143623 (*IL1B*)	0.88 (0.53-1.48)	0.6257	0.9386	0.91 (0.52-1.61)	0.7414	0.9132	0.97 (0.62-1.52)	0.8972	0.9654
rs1800871 (*IL10*)	0.97 (0.76-1.23)	0.7711	0.9423	0.96 (0.73-1.26)	0.7573	0.9132	0.97 (0.73-1.28)	0.8116	0.9390
rs1800872 (*IL10*)	0.97 (0.77-1.24)	0.8167	0.9423	0.97 (0.74-1.26)	0.7897	0.9145	0.97 (0.74-1.28)	0.8289	0.9390
rs1800896 (*IL10*)	0.92 (0.74-1.14)	0.4460	0.8649	0.89 (0.71-1.13)	0.3205	0.8900	0.90 (0.69-1.18)	0.4362	0.8769
rs2243248 (*IL4*)	0.87 (0.63-1.20)	0.3793	0.8649	0.88 (0.63-1.23)	0.4483	0.8900	0.85 (0.57-1.26)	0.4011	0.8769
rs2243250 (*IL4*)	1.17 (0.86-1.59)	0.3133	0.8649	1.21 (0.86-1.70)	0.2544	0.8900	1.31 (0.93-1.84)	0.1138	0.6453
rs2243270 (*IL4*)	0.98 (0.69-1.37)	0.8836	0.9667	1.02 (0.71-1.47)	0.8936	0.9415	1.07 (0.74-1.55)	0.6959	0.9390
rs1801275 (*IL4R*)	1.09 (0.88-1.35)	0.4177	0.8649	1.04 (0.81-1.33)	0.7477	0.9132	0.95 (0.71-1.28)	0.7314	0.9390
rs1805015 (*IL4R*)	1.05 (0.82-1.36)	0.6719	0.9423	1.08 (0.82-1.41)	0.5761	0.9132	0.98 (0.76-1.27)	0.9010	0.9654
rs5918 (*ITGB3*)	1.05 (0.80-1.37)	0.7073	0.9423	0.96 (0.71-1.28)	0.7584	0.9132	1.05 (0.73-1.50)	0.7903	0.9390
rs11003125 (*MBL2*)	1.17 (0.84-1.63)	0.3247	0.8649	1.23 (0.89-1.70)	0.1927	0.8020	1.31 (0.86-1.97)	0.1948	0.7792
rs1800450 (*MBL2*)	1.54 (0.75-3.14)	0.2249	0.8649	1.63 (0.84-3.15)	0.1395	0.7060	1.64 (0.78-3.48)	0.1834	0.7792
rs1800451 (*MBL2*)	1.15 (0.89-1.50)	0.2785	0.8649	1.18 (0.87-1.60)	0.2800	0.8900	1.16 (0.84-1.61)	0.3540	0.8769
rs5030737 (*MBL2*)	0.80 (0.19-3.28)	0.7428	0.9423	1.04 (0.22-4.97)	0.9581	0.9917	1.48 (0.30-7.25)	0.6113	0.9390
rs7096206 (*MBL2*)	0.70 (0.50-0.99)	**0.0423**	0.5340	0.69 (0.49-0.97)	**0.0333**	0.6549	0.66 (0.44-1.01)	0.0530	0.5280
rs1800482 (*NOS2A*)	1.31 (0.95-1.83)	0.0994	0.6582	1.27 (0.87-1.87)	0.2039	0.8020	1.45 (0.97-2.18)	0.0704	0.5280
rs9282799 (*NOS2A*)	0.77 (0.44-1.36)	0.3525	0.8649	0.92 (0.50-1.71)	0.7905	0.9145	0.79 (0.41-1.51)	0.4580	0.8769
rs1799983 (*NOS3*)	1.00 (0.72-1.38)	0.9845	0.9897	0.97 (0.69-1.37)	0.8626	0.9253	1.04 (0.72-1.50)	0.8338	0.9390
rs2070744 (*NOS3*)	0.99 (0.72-1.36)	0.9455	0.9897	0.97 (0.74-1.27)	0.8082	0.9170	1.00 (0.78-1.29)	0.9804	0.9970
rs662 (*PON1*)	1.05 (0.83-1.34)	0.6635	0.9423	1.12 (0.85-1.48)	0.4019	0.8900	1.04 (0.79-1.37)	0.7836	0.9390
rs854560 (*PON1*)	0.99 (0.67-1.48)	0.9663	0.9897	0.92 (0.60-1.41)	0.6879	0.9132	0.88 (0.58-1.35)	0.5543	0.9238
rs1801282 (*PPARG*)	0.78 (0.37-1.64)	0.4901	0.8649	0.87 (0.41-1.86)	0.7037	0.9132	0.89 (0.39-2.04)	0.7724	0.9390
rs1799762 (*SERPINE1*)	1.11 (0.83-1.49)	0.4528	0.8649	1.11 (0.81-1.51)	0.5129	0.8900	1.05 (0.76-1.45)	0.7591	0.9390
rs1800468 (*TGFB1/B9D2*)	0.48 (0.24-0.96)	**0.0384**	0.5340	0.47 (0.21-1.03)	0.0599	0.6818	0.44 (0.18-1.05)	0.0622	0.5280
rs1800469 (*TGFB1/B9D2*)	1.07 (0.82-1.39)	0.6106	0.9386	1.03 (0.76-1.40)	0.8445	0.9227	1.10 (0.77-1.56)	0.5921	0.9390
rs1800470 (*TGFB1*)	1.13 (0.86-1.48)	0.3794	0.8649	1.12 (0.82-1.53)	0.4482	0.8900	1.23 (0.86-1.75)	0.2385	0.8418
rs4986790 (*TLR4*)	0.80 (0.47-1.38)	0.4045	0.8649	0.80 (0.45-1.41)	0.4219	0.8900	0.73 (0.36-1.47)	0.3632	0.8769
rs1800629 (*TNF*)	0.69 (0.47-0.99)	**0.0468**	0.5340	0.71 (0.49-1.03)	0.0710	0.6818	0.79 (0.54-1.16)	0.2172	0.8145
rs1800750 (*TNF*)	0.68 (0.29-1.56)	0.3451	0.8649	0.73 (0.29-1.84)	0.4837	0.8900	0.64 (0.24-1.73)	0.3650	0.8769
rs361525 (*TNF*)	0.59 (0.31-1.14)	0.1097	0.6582	0.62 (0.30-1.27)	0.1768	0.8020	0.54 (0.25-1.15)	0.1059	0.6453
rs2239185 (*VDR*)	0.95 (0.77-1.17)	0.6176	0.9386	0.93 (0.76-1.15)	0.5104	0.8900	0.91 (0.75-1.12)	0.3720	0.8769
rs731236 (*VDR*)	1.25 (0.98-1.59)	0.0736	0.6309	1.25 (0.96-1.63)	0.0903	0.6818	1.33 (1.03-1.72)	**0.0301**	0.4515
rs890945 (Chr 5q33.3)	0.78 (0.61-1.00)	0.0534	0.5340	0.76 (0.59-1.00)	**0.0481**	0.6818	0.71 (0.53-0.95)	**0.0236**	0.4515

Variant	Crude Model			Age-Adjusted Model			Fully Adjusted[b] Model		
	OR (95% CI)	P-value	FDR-adjusted P-value	OR (95% CI)	P-value	FDR-adjusted P-value	OR (95% CI)	P-value	FDR-adjusted P-value
Mexican Americans									
rs1042713 (*ADRB2*)	1.12 (0.77-1.62)	0.5408	0.8280	1.16 (0.81-1.66)	0.4071	0.8311	1.11 (0.76-1.62)	0.5810	0.8327
rs1042714 (*ADRB2*)	1.03 (0.77-1.39)	0.8167	0.9266	1.02 (0.78-1.32)	0.9038	0.9459	1.07 (0.82-1.39)	0.6119	0.8450
rs429358 (*APOE*)	1.18 (0.84-1.64)	0.3265	0.7705	1.20 (0.86-1.68)	0.2721	0.7432	1.20 (0.86-1.67)	0.2676	0.7318
rs7412 (*APOE*)	0.65 (0.37-1.15)	0.1300	0.5012	0.66 (0.35-1.28)	0.2094	0.6918	0.43 (0.17-1.07)	0.0681	0.4140
rs769214 (*CAT*)	1.12 (0.86-1.47)	0.3889	0.8014	1.11 (0.84-1.47)	0.4299	0.8311	1.11 (0.79-1.55)	0.5338	0.8166
rs2280788 (*CCL5*)	0.68 (0.11-4.17)	0.6603	0.9060	0.67 (0.11-4.11)	0.6530	0.8853	0.74 (0.12-4.38)	0.7285	0.8990
rs1799864 (*CCR2*)	1.03 (0.76-1.39)	0.8486	0.9447	1.10 (0.81-1.49)	0.5324	0.8823	1.09 (0.78-1.54)	0.5886	0.8327
rs1205 (*CRP*)	1.01 (0.77-1.33)	0.9527	0.9691	0.99 (0.74-1.32)	0.9210	0.9459	0.98 (0.72-1.34)	0.8972	0.9461
rs1417938 (*CRP*)	1.12 (0.81-1.54)	0.4809	0.8014	1.09 (0.80-1.50)	0.5586	0.8853	1.10 (0.81-1.50)	0.5235	0.8166
rs1800947 (*CRP*)	0.52 (0.16-1.70)	0.2661	0.7136	0.38 (0.10-1.36)	0.1284	0.4655	0.26 (0.06-1.14)	0.0729	0.4140
rs2808630 (*CRP*)	0.81 (0.54-1.23)	0.3103	0.7705	0.83 (0.56-1.24)	0.3535	0.8220	0.84 (0.56-1.28)	0.4084	0.7540
rs3091244 (*CRP*)[c]	0.41 (0.21-0.81)	0.0692	0.4745	0.44 (0.24-0.81)	0.0681	0.3546	0.47 (0.25-0.89)	0.1002	0.4151
	1.09 (0.82-1.47)			1.07 (0.82-1.40)			1.10 (0.83-1.47)		
rs3093058 (*CRP*)	1.37 (0.66-2.83)	0.3790	0.8014	1.36 (0.53-3.52)	0.5047	0.8823	1.80 (0.78-4.18)	0.1615	0.5084
rs3093066 (*CRP*)	0.28 (0.06-1.26)	0.0946	0.4745	0.27 (0.06-1.18)	0.0790	0.3546	0.18 (0.03-1.26)	0.0815	0.4140
rs11265260 (*CRP*)	0.39 (0.21-0.72)	**0.0041**	0.1121	0.44 (0.26-0.74)	**0.0036**	0.1044	0.51 (0.29-0.92)	**0.0274**	0.3202
rs12093699 (*CRP*)	1.07 (0.80-1.42)	0.6491	0.9060	1.05 (0.80-1.38)	0.7021	0.8853	1.01 (0.78-1.31)	0.9239	0.9569
rs12744244 (*CRP*)	0.78 (0.43-1.43)	0.4114	0.8014	0.76 (0.41-1.39)	0.3543	0.8220	0.72 (0.45-1.14)	0.1555	0.5084
rs2027471 (*CRP*)	1.00 (0.79-1.25)	0.9871	0.9871	0.98 (0.77-1.24)	0.8500	0.9337	0.97 (0.75-1.24)	0.7747	0.9149
rs2592887 (*CRP*)	0.99 (0.79-1.24)	0.9525	0.9691	0.97 (0.75-1.25)	0.8041	0.9328	0.98 (0.75-1.28)	0.8538	0.9335
rs2794520 (*CRP*)	0.99 (0.78-1.26)	0.9337	0.9691	0.97 (0.75-1.25)	0.7896	0.9328	0.97 (0.74-1.27)	0.8045	0.9149
rs3093075 (*CRP*)	0.40 (0.22-0.75)	**0.0057**	0.1121	0.43 (0.24-0.77)	**0.0063**	0.1218	0.45 (0.26-0.80)	**0.0088**	0.2069
rs1799963 (*F2*)	1.84 (0.49-6.90)	0.3468	0.7870	1.84 (0.46-7.39)	0.3730	0.8311	2.12 (0.51-8.74)	0.2833	0.7318
rs6025 (*F5*)	1.59 (0.48-5.23)	0.4304	0.8014	1.55 (0.42-5.68)	0.4896	0.8823	1.74 (0.43-7.05)	0.4229	0.7540
rs1801274 (*FCGR2A*)	1.22 (0.90-1.66)	0.1969	0.5809	1.21 (0.87-1.67)	0.2405	0.7342	1.19 (0.85-1.67)	0.3014	0.7318
rs1800790 (*FGB*)	0.77 (0.53-1.10)	0.1417	0.5012	0.82 (0.57-1.18)	0.2697	0.7432	0.91 (0.58-1.42)	0.6621	0.8534
rs1260326 (*GCKR*)	1.08 (0.83-1.40)	0.5621	0.8280	1.07 (0.82-1.38)	0.6145	0.8853	1.12 (0.87-1.43)	0.3646	0.7540
rs1143623 (*IL1B*)	1.24 (1.09-1.40)	**0.0022**	0.1121	1.36 (1.20-1.55)	**0.0001**	**0.0058**	1.46 (1.28-1.67)	**<0.0001**	**<0.0001**
rs1800871 (*IL10*)	1.29 (1.03-1.62)	**0.0267**	0.3151	1.28 (0.98-1.68)	0.0650	0.3546	1.22 (0.91-1.62)	0.1753	0.5084
rs1800872 (*IL10*)	1.30 (1.04-1.64)	**0.0242**	0.3151	1.29 (0.99-1.68)	0.0601	0.3546	1.22 (0.91-1.63)	0.1673	0.5084
rs1800896 (*IL10*)	0.70 (0.47-1.04)	0.0749	0.4745	0.68 (0.44-1.06)	0.0856	0.3546	0.70 (0.44-1.11)	0.1202	0.4648
rs2243248 (*IL4*)	0.87 (0.54-1.42)	0.5754	0.8280	0.98 (0.61-1.57)	0.9175	0.9459	0.96 (0.62-1.50)	0.8691	0.9335
rs2243250 (*IL4*)	0.96 (0.70-1.31)	0.7759	0.9266	0.99 (0.71-1.37)	0.9296	0.9459	0.96 (0.69-1.33)	0.7936	0.9149
rs2243270 (*IL4*)	0.89 (0.64-1.24)	0.4722	0.8014	0.91 (0.65-1.28)	0.5695	0.8853	0.91 (0.65-1.27)	0.5484	0.8166
rs1801275 (*IL4R*)	0.98 (0.71-1.36)	0.9170	0.9691	1.03 (0.71-1.50)	0.8532	0.9337	1.01 (0.65-1.57)	0.9570	0.9738
rs1805015 (*IL4R*)	1.05 (0.78-1.42)	0.7271	0.9266	1.08 (0.77-1.51)	0.6528	0.8853	1.12 (0.68-1.84)	0.6553	0.8534
rs5918 (*ITGB3*)	1.22 (0.76-1.97)	0.3958	0.8014	1.23 (0.74-2.06)	0.4125	0.8311	1.27 (0.73-2.21)	0.3860	0.7540
rs11003125 (*MBL2*)	0.95 (0.67-1.36)	0.7798	0.9266	1.00 (0.70-1.43)	1.0000	1.0000	1.03 (0.71-1.50)	0.8658	0.9335
rs1800450 (*MBL2*)	1.19 (0.75-1.89)	0.4502	0.8014	1.10 (0.67-1.81)	0.6937	0.8853	1.23 (0.74-2.05)	0.3997	0.7540
rs1800451 (*MBL2*)	0.91 (0.40-2.06)	0.8155	0.9266	0.87 (0.34-2.22)	0.7588	0.9169	0.81 (0.32-2.09)	0.6567	0.8534
rs5030737 (*MBL2*)	2.02 (0.93-4.38)	0.0742	0.4745	2.24 (0.90-5.61)	0.0813	0.3546	2.31 (0.88-6.07)	0.0865	0.4140
rs7096206 (*MBL2*)	1.18 (0.74-1.89)	0.4720	0.8014	1.14 (0.64-2.03)	0.6541	0.8853	0.90 (0.52-1.55)	0.6870	0.8662
rs1800482 (*NOS2A*)	1.29 (0.22-7.53)	0.7666	0.9266	1.72 (0.31-9.59)	0.5195	0.8823			
rs9282799 (*NOS2A*)									
rs1799983 (*NOS3*)	0.77 (0.51-1.16)	0.1955	0.5809	0.70 (0.47-1.04)	0.0760	0.3546	0.70 (0.46-1.07)	0.0928	0.4140
rs2070744 (*NOS3*)	0.76 (0.54-1.08)	0.1157	0.5012	0.70 (0.49-1.00)	0.0524	0.3546	0.67 (0.47-0.95)	**0.0276**	0.3202
rs662 (*PON1*)	1.05 (0.80-1.38)	0.7055	0.9266	1.10 (0.85-1.43)	0.4511	0.8440	1.13 (0.78-1.63)	0.4929	0.8166
rs854560 (*PON1*)	0.75 (0.52-1.06)	0.0965	0.4745	0.68 (0.48-0.96)	**0.0311**	0.3546	0.67 (0.43-1.04)	0.0735	0.4140
rs1801282 (*PPARG*)	0.77 (0.53-1.10)	0.1444	0.5012	0.79 (0.53-1.16)	0.2147	0.6918	0.85 (0.56-1.29)	0.4290	0.7540
rs1799762 (*SERPINE1*)	1.04 (0.82-1.31)	0.7462	0.9266	0.96 (0.76-1.22)	0.7411	0.9145	0.88 (0.68-1.14)	0.3028	0.7318
rs1800468 (*TGFB1/B9D2*)	0.70 (0.41-1.19)	0.1785	0.5809	0.62 (0.37-1.04)	0.0694	0.3546	0.67 (0.37-1.21)	0.1709	0.5084
rs1800469 (*TGFB1/B9D2*)	1.08 (0.86-1.35)	0.4890	0.8014	1.10 (0.87-1.38)	0.4183	0.8311	1.08 (0.83-1.41)	0.5342	0.8166
rs1800470 (*TGFB1*)	1.13 (0.88-1.45)	0.3190	0.7705	1.15 (0.88-1.50)	0.3013	0.7598	1.16 (0.84-1.58)	0.3551	0.7540
rs4986790 (*TLR4*)	0.58 (0.27-1.22)	0.1427	0.5012	0.56 (0.28-1.13)	0.1012	0.3913	0.53 (0.26-1.09)	0.0814	0.4140
rs1800629 (*TNF*)	0.63 (0.38-1.04)	0.0697	0.4745	0.58 (0.35-0.95)	**0.0322**	0.3546	0.56 (0.36-0.86)	**0.0107**	0.2069
rs1800750 (*TNF*)	1.44 (0.44-4.77)	0.5345	0.8280	1.29 (0.38-4.38)	0.6733	0.8853	1.00 (0.44-2.28)	1.0000	1.0000
rs361525 (*TNF*)	0.91 (0.40-2.04)	0.8044	0.9266	0.92 (0.39-2.16)	0.8417	0.9337	0.78 (0.44-1.37)	0.3722	0.7540
rs2239185 (*VDR*)	0.88 (0.72-1.08)	0.2121	0.5959	0.88 (0.69-1.12)	0.2819	0.7432	0.91 (0.67-1.25)	0.5491	0.8166
rs731236 (*VDR*)	0.92 (0.68-1.24)	0.5744	0.8280	0.94 (0.69-1.27)	0.6714	0.8853	0.84 (0.58-1.24)	0.3659	0.7540
rs890945 (Chr 5q33.3)	1.02 (0.78-1.34)	0.8781	0.9594	1.07 (0.81-1.41)	0.6297	0.8853	1.05 (0.74-1.48)	0.7788	0.9149

CI, confidence interval; FDR, false-discovery rate; OR, odds ratio. Variants with missing results had unstable statistical models.

a) Defined as urinary albumin-to-creatinine ratio (ACR) above sex-specific thresholds (≥ 17 mg/g in men and ≥ 25 mg/g in women). b) Analyses adjusted for age, alcohol consumption, educational attainment, and waist:hip ratio. c) For this tri-allelic variant, the first beta coefficient corresponds to the A allele, while the second corresponds to the T allele. (The C allele is the reference.) The unadjusted and FDR-adjusted P values are for the overall test of association.

Table S4. Complete results of associations of candidate gene polymorphisms and sex-specific albuminuria[a], codominant genetic model

Variant	Genotype	Crude Model			Age-Adjusted Model			Fully Adjusted[b] Model		
		OR (95% CI)	P-value	FDR-adjusted P-value	OR (95% CI)	P-value	FDR-adjusted P-value	OR (95% CI)	P-value	FDR-adjusted P-value
Non-Hispanic whites										
rs1042713 (*ADRB2*)	AA vs. GG<br>AG vs. GG	0.94 (0.55-1.61)<br>0.96 (0.63-1.46)	0.9569	0.9612	1.00 (0.61-1.63)<br>0.96 (0.62-1.48)	0.9668	0.9847	1.01 (0.58-1.76)<br>0.94 (0.57-1.57)	0.9478	0.9720
rs1042714 (*ADRB2*)	GG vs. CC<br>GC vs. CC	1.00 (0.64-1.59)<br>0.94 (0.67-1.32)	0.9173	0.9612	1.02 (0.63-1.64)<br>0.86 (0.62-1.20)	0.6227	0.8952	0.95 (0.55-1.65)<br>0.88 (0.61-1.27)	0.7888	0.9239
rs429358 (*APOE*)	CC vs. TT<br>CT vs. TT	0.49 (0.19-1.27)<br>0.90 (0.62-1.33)	0.4225	0.8703	0.58 (0.24-1.43)<br>0.97 (0.66-1.43)	0.6161	0.8952	0.56 (0.23-1.35)<br>1.00 (0.66-1.51)	0.5895	0.8783
rs7412 (*APOE*)	TT vs. CC<br>TC vs. CC	0.45 (0.04-4.95)<br>1.33 (0.93-1.91)	0.2051	0.8703	0.41 (0.05-3.63)<br>1.24 (0.88-1.74)	0.2911	0.8952	0.42 (0.05-3.66)<br>1.21 (0.82-1.79)	0.4067	0.8783
rs769214 (*CAT*)	GG vs. AA<br>GA vs. AA	1.04 (0.59-1.86)<br>0.89 (0.62-1.28)	0.7436	0.9017	1.07 (0.62-1.86)<br>0.85 (0.60-1.20)	0.5521	0.8952	1.09 (0.61-1.96)<br>0.85 (0.60-1.20)	0.5356	0.8783
rs2280788 (*CCL5*)	GG vs. CC<br>GC vs. CC	3.02 (0.53-17.07)<br>0.91 (0.56-1.50)	0.3874	0.8703	4.07 (0.29-57.93)<br>1.08 (0.65-1.81)	0.4272	0.8952	5.71 (0.11-292.34)<br>1.01 (0.59-1.72)	0.4735	0.8783
rs1799864 (*CCR2*)	AA vs. GG<br>AG vs. GG	1.25 (0.25-6.16)<br>1.10 (0.78-1.57)	0.8311	0.9475	1.37 (0.31-6.01)<br>1.12 (0.75-1.69)	0.7807	0.8952	1.02 (0.25-4.23)<br>1.23 (0.83-1.82)	0.5658	0.8783
rs1205 (*CRP*)	AA vs. GG<br>AG vs. GG	0.88 (0.62-1.24)<br>0.87 (0.68-1.10)	0.4216	0.8703	1.01 (0.70-1.45)<br>0.86 (0.67-1.11)	0.4001	0.8952	1.10 (0.75-1.62)<br>0.86 (0.65-1.16)	0.3860	0.8783
rs1417938 (*CRP*)	TT vs. AA<br>TA vs. AA	1.33 (0.72-2.45)<br>0.98 (0.65-1.47)	0.5837	0.8703	1.24 (0.70-2.18)<br>0.92 (0.62-1.38)	0.5952	0.8952	1.03 (0.54-1.97)<br>0.86 (0.56-1.32)	0.6984	0.9095
rs1800947 (*CRP*)	CC vs. GG<br>CG vs. GG									
rs2808630 (*CRP*)	GG vs. AA<br>GA vs. AA	0.79 (0.42-1.47)<br>1.00 (0.63-1.61)	0.7481	0.9017	0.86 (0.51-1.47)<br>1.01 (0.65-1.55)	0.8477	0.9278	0.93 (0.54-1.59)<br>1.09 (0.69-1.73)	0.7865	0.9239
rs3091244 (*CRP*)	AA vs. CC<br>AC vs. CC<br>AT vs. CC<br>CT vs. CC<br>TT vs. CC	0.84 (0.07-10.54)<br>1.09 (0.55-2.17)<br>1.25 (0.63-2.50)<br>1.02 (0.65-1.58)<br>1.36 (0.73-2.55)	0.8184	0.9475	0.52 (0.04-6.83)<br>1.03 (0.48-2.21)<br>1.19 (0.61-2.32)<br>0.95 (0.62-1.47)<br>1.28 (0.72-2.26)	0.8406	0.9278	0.43 (0.03-6.02)<br>1.02 (0.42-2.44)<br>1.14 (0.53-2.49)<br>0.91 (0.60-1.40)<br>1.08 (0.55-2.11)	0.9366	0.9720
rs3093058 (*CRP*)	TT vs. AA<br>TA vs. AA	 2.56 (0.41-15.87)	0.2974	0.8703	 2.48 (0.50-12.39)	0.2540	0.8952	 2.30 (0.39-13.58)	0.3422	0.8783
rs3093066 (*CRP*)	AA vs. CC<br>AC vs. CC	 1.17 (0.40-3.42)	0.7593	0.9017	 1.17 (0.39-3.49)	0.7680	0.8952	 1.64 (0.65-4.11)	0.2804	0.8783
rs11265260 (*CRP*)	GG vs. AA<br>GA vs. AA									
rs12093699 (*CRP*)	AA vs. GG<br>AG vs. GG	1.31 (0.70-2.42)<br>1.11 (0.68-1.80)	0.6986	0.9017	1.25 (0.69-2.28)<br>1.05 (0.65-1.68)	0.7813	0.8952	1.11 (0.56-2.21)<br>1.02 (0.65-1.59)	0.9546	0.9720
rs12744244 (*CRP*)	AA vs. CC<br>AC vs. CC	1.10 (0.44-2.80)<br>1.28 (0.79-2.07)	0.5171	0.8703	1.11 (0.42-2.96)<br>1.20 (0.73-1.97)	0.7023	0.8952	0.85 (0.29-2.53)<br>1.21 (0.78-1.87)	0.5960	0.8783
rs2027471 (*CRP*)	AA vs. TT<br>AT vs. TT	0.83 (0.59-1.17)<br>0.84 (0.66-1.06)	0.2637	0.8703	0.94 (0.64-1.39)<br>0.85 (0.66-1.09)	0.4146	0.8952	1.06 (0.71-1.59)<br>0.86 (0.64-1.15)	0.4219	0.8783
rs2592887 (*CRP*)	AA vs. GG<br>AG vs. GG	0.82 (0.58-1.18)<br>0.93 (0.68-1.26)	0.5955	0.8703	0.86 (0.60-1.25)<br>0.94 (0.69-1.28)	0.7447	0.8952	0.93 (0.64-1.35)<br>0.91 (0.64-1.29)	0.8094	0.9239
rs2794520 (*CRP*)	AA vs. GG<br>AG vs. GG	0.93 (0.66-1.31)<br>0.88 (0.70-1.10)	0.5165	0.8703	1.08 (0.74-1.58)<br>0.88 (0.70-1.11)	0.4124	0.8952	1.13 (0.76-1.69)<br>0.90 (0.69-1.19)	0.4886	0.8783
rs3093075 (*CRP*)	AA vs. CC<br>AC vs. CC	0.80 (0.07-8.73)<br>1.06 (0.72-1.55)	0.9277	0.9612	0.52 (0.05-5.87)<br>1.00 (0.64-1.56)	0.8603	0.9278	0.44 (0.04-5.42)<br>1.02 (0.60-1.72)	0.7888	0.9239
rs1799963 (*F2*)	AG vs. GG	1.88 (0.87-4.03)	0.1024	0.8703	1.57 (0.65-3.76)	0.2981	0.8952	1.79 (0.71-4.52)	0.2062	0.8783
rs6025 (*F5*)	AA vs. GG<br>AG vs. GG									
rs1801274 (*FCGR2A*)	AA vs. GG<br>AG vs. GG	1.07 (0.73-1.58)<br>0.80 (0.59-1.08)	0.1935	0.8703	1.09 (0.72-1.65)<br>0.89 (0.64-1.23)	0.4775	0.8952	1.16 (0.72-1.89)<br>0.93 (0.66-1.32)	0.4952	0.8783
rs1800790 (*FGB*)	AA vs. GG<br>AG vs. GG	1.14 (0.61-2.15)<br>0.90 (0.72-1.13)	0.5780	0.8703	1.13 (0.56-2.27)<br>0.93 (0.73-1.17)	0.7370	0.8952	1.01 (0.45-2.26)<br>0.91 (0.66-1.24)	0.8249	0.9239
rs1260326 (*GCKR*)	TT vs. CC<br>TC vs. CC	1.16 (0.66-2.04)<br>1.42 (0.95-2.13)	0.1777	0.8703	1.28 (0.74-2.23)<br>1.50 (0.99-2.28)	0.1180	0.8952	1.39 (0.83-2.31)<br>1.53 (1.04-2.25)	0.0926	0.8783
rs1143623 (*IL1B*)	CC vs. GG<br>CG vs. GG	1.00 (0.46-2.18)<br>1.07 (0.79-1.46)	0.8650	0.9612	1.11 (0.49-2.51)<br>1.02 (0.75-1.41)	0.8991	0.9510	1.06 (0.48-2.37)<br>1.13 (0.85-1.50)	0.7308	0.9239
rs1800871 (*IL10*)	TT vs. CC<br>TC vs. CC	0.80 (0.45-1.42)<br>1.10 (0.78-1.56)	0.5783	0.8703	0.78 (0.43-1.41)<br>1.14 (0.80-1.63)	0.4548	0.8952	0.79 (0.46-1.36)<br>1.15 (0.79-1.67)	0.4572	0.8783
rs1800872 (*IL10*)	AA vs. CC<br>AC vs. CC	0.73 (0.40-1.33)<br>1.08 (0.79-1.46)	0.4786	0.8703	0.72 (0.39-1.33)<br>1.12 (0.82-1.51)	0.3895	0.8952	0.76 (0.44-1.33)<br>1.14 (0.81-1.60)	0.4257	0.8783
rs1800896 (*IL10*)	GG vs. AA<br>GA vs. AA	0.86 (0.48-1.52)<br>1.27 (0.93-1.73)	0.1806	0.8703	0.85 (0.47-1.53)<br>1.22 (0.89-1.67)	0.2558	0.8952	0.87 (0.48-1.55)<br>1.24 (0.89-1.72)	0.2681	0.8783
rs2243248 (*IL4*)	GG vs. TT<br>GT vs. TT	0.51 (0.07-3.69)<br>1.09 (0.70-1.71)	0.7217	0.9017	0.64 (0.11-3.79)<br>1.31 (0.86-1.99)	0.2749	0.8952	0.22 (0.03-1.98)<br>1.22 (0.73-2.04)	0.4080	0.8783
rs2243250 (*IL4*)	TT vs. CC<br>TC vs. CC	0.60 (0.20-1.78)<br>0.97 (0.66-1.41)	0.6361	0.8843	0.59 (0.19-1.80)<br>0.95 (0.66-1.37)	0.6132	0.8952	0.56 (0.18-1.72)<br>0.97 (0.69-1.37)	0.5615	0.8783
rs2243270 (*IL4*)	GG vs. AA<br>GA vs. AA	0.58 (0.19-1.78)<br>0.96 (0.70-1.32)	0.5878	0.8703	0.58 (0.19-1.81)<br>0.95 (0.69-1.29)	0.5784	0.8952	0.64 (0.21-1.93)<br>0.95 (0.71-1.28)	0.6520	0.8805
rs1801275 (*IL4R*)	GG vs. AA<br>GA vs. AA	1.08 (0.55-2.09)<br>0.99 (0.70-1.38)	0.9612	0.9612	1.34 (0.61-2.94)<br>1.02 (0.74-1.41)	0.6793	0.8952	1.08 (0.45-2.60)<br>1.10 (0.75-1.63)	0.8096	0.9239

Variant	Genotype	Crude Model			Age-Adjusted Model			Fully Adjusted[b] Model		
		OR (95% CI)	P-value	FDR-adjusted P-value	OR (95% CI)	P-value	FDR-adjusted P-value	OR (95% CI)	P-value	FDR-adjusted P-value
rs1805015 (*IL4R*)	CC vs. TT<br>CT vs. TT	1.54 (0.71-3.33)<br>0.94 (0.61-1.45)	0.5469	0.8703	1.98 (0.84-4.64)<br>0.95 (0.61-1.48)	0.3261	0.8952	1.62 (0.61-4.34)<br>0.99 (0.61-1.59)	0.6170	0.8805
rs5918 (*ITGB3*)	CC vs. TT<br>CT vs. TT	1.06 (0.43-2.61)<br>1.04 (0.67-1.62)	0.9595	0.9612	1.10 (0.50-2.43)<br>1.04 (0.63-1.71)	0.9367	0.9720	1.24 (0.56-2.75)<br>1.00 (0.67-1.49)	0.8712	0.9382
rs11003125 (*MBL2*)	GG vs. CC<br>GC vs. CC	1.57 (0.95-2.60)<br>1.21 (0.84-1.73)	0.1587	0.8703	1.51 (0.91-2.51)<br>1.14 (0.81-1.62)	0.2371	0.8952	1.60 (0.95-2.69)<br>1.04 (0.73-1.49)	0.1253	0.8783
rs1800450 (*MBL2*)	AA vs. GG<br>AG vs. GG	1.45 (0.69-3.05)<br>1.08 (0.76-1.52)	0.5810	0.8703	1.37 (0.59-3.22)<br>1.03 (0.72-1.47)	0.7352	0.8952	1.54 (0.66-3.62)<br>0.99 (0.66-1.49)	0.5904	0.8783
rs1800451 (*MBL2*)	AA vs. GG<br>AG vs. GG	14.27 (0.99-204.93)<br>0.97 (0.51-1.86)	0.1376	0.8703						
rs5030737 (*MBL2*)	TT vs. CC<br>TC vs. CC	2.87 (0.45-18.41)<br>1.45 (0.81-2.58)	0.2312	0.8703	2.61 (0.25-27.41)<br>1.53 (0.84-2.77)	0.2655	0.8952	2.84 (0.21-37.68)<br>1.56 (0.83-2.91)	0.2848	0.8783
rs7096206 (*MBL2*)	CC vs. GG<br>CG vs. GG	1.38 (0.86-2.20)<br>0.79 (0.53-1.16)	0.1495	0.8703	1.42 (0.81-2.48)<br>0.77 (0.53-1.12)	0.1206	0.8952	1.77 (0.93-3.38)<br>0.78 (0.52-1.16)	0.0692	0.8783
rs1800482 (*NOS2A*)	CC vs. GG<br>CG vs. GG	3.86 (0.28-52.28)	0.2951	0.8703	1.82 (0.14-24.37)	0.6367	0.8952	2.04 (0.19-22.44)	0.5441	0.8783
rs9282799 (*NOS2A*)	TT vs. CC<br>TC vs. CC	3.76 (0.95-14.82)	0.0578	0.8703	1.77 (0.44-7.19)	0.4054	0.8952	1.74 (0.40-7.50)	0.4418	0.8783
rs1799983 (*NOS3*)	TT vs. GG<br>TG vs. GG	1.27 (0.71-2.29)<br>1.09 (0.84-1.41)	0.5700	0.8703	1.44 (0.82-2.54)<br>1.14 (0.86-1.51)	0.3227	0.8952	1.44 (0.84-2.49)<br>1.17 (0.87-1.57)	0.3025	0.8783
rs2070744 (*NOS3*)	CC vs. TT<br>CT vs. TT	0.91 (0.59-1.43)<br>0.98 (0.75-1.28)	0.8969	0.9612	0.99 (0.61-1.60)<br>1.01 (0.75-1.36)	0.9951	0.9951	1.00 (0.56-1.78)<br>1.00 (0.73-1.36)	0.9997	0.9997
rs662 (*PON1*)	GG vs. AA<br>GA vs. AA	1.09 (0.64-1.87)<br>0.91 (0.65-1.27)	0.7388	0.9017	1.20 (0.72-2.02)<br>0.98 (0.69-1.39)	0.7227	0.8952	1.39 (0.82-2.36)<br>1.07 (0.76-1.52)	0.4370	0.8783
rs854560 (*PON1*)	AA vs. TT<br>AT vs. TT	1.08 (0.63-1.84)<br>1.29 (1.04-1.59)	0.2417	0.8703	1.06 (0.63-1.78)<br>1.23 (0.96-1.58)	0.3901	0.8952	0.93 (0.54-1.59)<br>1.19 (0.87-1.62)	0.4646	0.8783
rs1801282 (*PPARG*)	GG vs. CC<br>GC vs. CC	1.05 (0.31-3.54)<br>1.27 (0.91-1.77)	0.4385	0.8703	1.36 (0.50-3.71)<br>1.28 (0.92-1.77)	0.2971	0.8952	1.21 (0.26-5.62)<br>1.40 (0.97-2.03)	0.2861	0.8783
rs1799762 (*SERPINE1*)	4G4G vs. 5G5G<br>4G5G vs. 5G5G	1.22 (0.84-1.78)<br>1.39 (0.97-1.98)	0.3091	0.8703	1.28 (0.84-1.94)<br>1.42 (0.96-2.09)	0.3483	0.8952	1.36 (0.89-2.07)<br>1.58 (1.03-2.41)	0.2345	0.8783
rs1800468 (*TGFB1/B9D2*)	AA vs. GG<br>AG vs. GG	3.36 (0.74-15.33)<br>1.05 (0.65-1.71)	0.2865	0.8703	4.17 (0.75-23.15)<br>1.13 (0.68-1.89)	0.2318	0.8952	3.88 (0.64-23.42)<br>1.17 (0.73-1.89)	0.2327	0.8783
rs1800469 (*TGFB1/B9D2*)	TT vs. CC<br>TC vs. CC	1.26 (0.76-2.08)<br>1.11 (0.76-1.64)	0.6134	0.8741	1.34 (0.81-2.21)<br>1.06 (0.71-1.60)	0.5449	0.8952	1.35 (0.78-2.31)<br>1.03 (0.67-1.61)	0.5649	0.8783
rs1800470 (*TGFB1*)	CC vs. TT<br>CT vs. TT	1.37 (0.73-2.56)<br>1.12 (0.80-1.56)	0.4297	0.8703	1.35 (0.73-2.49)<br>1.10 (0.78-1.57)	0.4786	0.8952	1.32 (0.75-2.29)<br>1.08 (0.75-1.56)	0.5194	0.8783
rs4986790 (*TLR4*)	GG vs. AA<br>GA vs. AA	1.19 (0.27-5.35)<br>1.15 (0.77-1.72)	0.7359	0.9017	1.39 (0.26-7.30)<br>1.13 (0.76-1.67)	0.7553	0.8952	1.30 (0.23-7.14)<br>1.10 (0.73-1.66)	0.8531	0.9367
rs1800629 (*TNF*)	AA vs. GG<br>AG vs. GG	0.56 (0.28-1.13)<br>1.01 (0.57-1.80)	0.5918	0.8703	0.68 (0.33-1.40)<br>0.97 (0.55-1.70)	0.7444	0.8952	0.56 (0.26-1.22)<br>0.96 (0.52-1.76)	0.6343	0.8805
rs1800750 (*TNF*)	AA vs. GG<br>AG vs. GG	11.55 (0.53-250.67)<br>2.18 (1.19-3.97)	**0.0328**	0.8703	11.90 (1.30-108.82)<br>2.98 (1.52-5.84)	**0.0019**	0.1045	14.13 (1.88-105.96)<br>2.52 (0.95-6.70)	**0.0303**	0.8484
rs361525 (*TNF*)	AA vs. GG<br>AG vs. GG	3.91 (1.10-13.91)<br>1.40 (0.87-2.27)	**0.0488**	0.8703	6.04 (1.08-33.67)<br>1.58 (0.99-2.51)	**0.0299**	0.8223	11.71 (2.51-54.63)<br>1.53 (0.98-2.38)	**0.0026**	0.1456
rs2239185 (*VDR*)	CC vs. TT<br>CT vs. TT	0.74 (0.47-1.16)<br>0.97 (0.71-1.32)	0.3228	0.8703	0.78 (0.49-1.23)<br>0.99 (0.72-1.37)	0.4283	0.8952	0.89 (0.58-1.38)<br>1.09 (0.72-1.63)	0.6604	0.8805
rs731236 (*VDR*)	CC vs. TT<br>CT vs. TT	1.60 (1.01-2.51)<br>1.28 (0.92-1.79)	0.0856	0.8703	1.54 (1.00-2.37)<br>1.27 (0.91-1.77)	0.1031	0.8952	1.31 (0.86-1.99)<br>1.27 (0.87-1.85)	0.3302	0.8783
rs890945 (Chr 5q33.3)	AA vs. TT<br>AT vs. TT	1.31 (0.57-2.99)<br>1.18 (0.87-1.61)	0.5205	0.8703	1.57 (0.76-3.26)<br>1.20 (0.86-1.66)	0.2915	0.8952	1.18 (0.57-2.43)<br>1.18 (0.80-1.75)	0.5955	0.8783
Non-Hispanic blacks										
rs1042713 (*ADRB2*)	AA vs. GG<br>AG vs. GG	1.00 (0.62-1.61)<br>1.00 (0.71-1.40)	0.9996	0.9996	1.08 (0.65-1.79)<br>1.01 (0.73-1.40)	0.9067	0.9646	1.00 (0.54-1.83)<br>1.11 (0.73-1.69)	0.8323	0.8843
rs1042714 (*ADRB2*)	GG vs. CC<br>GC vs. CC	0.19 (0.05-0.75)<br>1.30 (0.86-1.98)	0.0699	0.8396	0.21 (0.05-0.79)<br>1.18 (0.76-1.81)	0.1422	0.7503	0.25 (0.07-0.93)<br>1.18 (0.72-1.94)	0.2126	0.6834
rs429358 (*APOE*)	CC vs. TT<br>CT vs. TT	1.02 (0.45-2.30)<br>0.96 (0.67-1.37)	0.9683	0.9877	0.99 (0.46-2.15)<br>1.10 (0.73-1.64)	0.8761	0.9646	0.86 (0.40-1.88)<br>1.08 (0.75-1.57)	0.8065	0.8843
rs7412 (*APOE*)	TT vs. CC<br>TC vs. CC	1.21 (0.23-6.44)<br>1.46 (0.96-2.23)	0.1418	0.8396	1.35 (0.34-5.38)<br>1.38 (0.94-2.04)	0.1625	0.7503	1.47 (0.36-5.97)<br>1.51 (0.98-2.35)	0.0988	0.6834
rs769214 (*CAT*)	GG vs. AA<br>GA vs. AA	0.86 (0.48-1.53)<br>0.87 (0.63-1.22)	0.7277	0.8810	0.84 (0.45-1.55)<br>0.87 (0.62-1.23)	0.7176	0.8970	0.83 (0.45-1.51)<br>0.90 (0.59-1.36)	0.7689	0.8843
rs2280788 (*CCL5*)	GG vs. CC<br>GC vs. CC	0.31 (0.03-2.76)	0.2796	0.8396	0.41 (0.04-4.20)	0.4354	0.8192	0.42 (0.04-4.86)	0.4677	0.7794
rs1799864 (*CCR2*)	AA vs. GG<br>AG vs. GG	1.25 (0.34-4.63)<br>0.84 (0.63-1.13)	0.5432	0.8810	1.37 (0.46-4.07)<br>0.85 (0.65-1.12)	0.4483	0.8192	1.24 (0.34-4.52)<br>0.85 (0.64-1.14)	0.5777	0.7794
rs1205 (*CRP*)	AA vs. GG<br>AG vs. GG	0.47 (0.16-1.38)<br>0.91 (0.68-1.22)	0.2519	0.8396	0.39 (0.13-1.14)<br>0.98 (0.70-1.36)	0.1468	0.7503	0.36 (0.10-1.31)<br>0.97 (0.68-1.37)	0.1860	0.6834
rs1417938 (*CRP*)	TT vs. AA<br>TA vs. AA									
rs1800947 (*CRP*)	CC vs. GG<br>CG vs. GG	0.68 (0.17-2.68)	0.5658	0.8810	0.50 (0.13-1.94)	0.3031	0.7578	0.57 (0.15-2.14)	0.3853	0.7794
rs2808630 (*CRP*)	GG vs. AA<br>GA vs. AA	1.05 (0.38-2.92)<br>1.59 (1.17-2.14)	**0.0218**	0.6222	0.82 (0.30-2.22)<br>1.59 (1.19-2.12)	**0.0172**	0.7503	0.66 (0.24-1.76)<br>1.62 (1.16-2.28)	**0.0141**	0.4157

Variant	Genotype	Crude Model			Age-Adjusted Model			Fully Adjusted[b] Model		
		**OR (95% CI)**	**P-value**	**FDR-adjusted P-value**	**OR (95% CI)**	**P-value**	**FDR-adjusted P-value**	**OR (95% CI)**	**P-value**	**FDR-adjusted P-value**
rs3091244 (*CRP*)	AA vs. CC<br>AC vs. CC<br>AT vs. CC<br>CT vs. CC<br>TT vs. CC	0.76 (0.42-1.39)<br>1.06 (0.58-1.96)<br>0.94 (0.46-1.90)<br>0.75 (0.44-1.28)<br>0.54 (0.23-1.26)	0.4676	0.8810	0.79 (0.45-1.39)<br>1.16 (0.61-2.20)<br>1.03 (0.54-1.96)<br>0.87 (0.48-1.56)<br>0.55 (0.21-1.42)	0.5084	0.8192	0.65 (0.33-1.29)<br>1.38 (0.72-2.66)<br>1.15 (0.54-2.44)<br>0.92 (0.47-1.79)<br>0.78 (0.30-2.02)	0.4346	0.7794
rs3093058 (*CRP*)	TT vs. AA<br>TA vs. AA	0.48 (0.17-1.36)<br>0.66 (0.48-0.91)	**0.0244**	0.6222	0.49 (0.16-1.44)<br>0.68 (0.50-0.92)	**0.0339**	0.7503	0.54 (0.18-1.65)<br>0.63 (0.45-0.89)	**0.0321**	0.4157
rs3093066 (*CRP*)	AA vs. CC<br>AC vs. CC	1.09 (0.57-2.08)<br>1.37 (0.83-2.28)	0.2918	0.8396	1.22 (0.64-2.35)<br>1.39 (0.87-2.21)	0.2390	0.7503	0.79 (0.31-1.99)<br>1.46 (0.90-2.37)	0.1674	0.6834
rs11265260 (*CRP*)	GG vs. AA<br>GA vs. AA	4.60 (0.76-27.94)<br>0.89 (0.52-1.53)	0.2351	0.8396	6.52 (1.15-36.96)<br>0.87 (0.45-1.68)	0.1800	0.7503	7.86 (1.40-44.18)<br>0.92 (0.45-1.88)	0.1509	0.6834
rs12093699 (*CRP*)	AA vs. GG<br>AG vs. GG	0.86 (0.52-1.43)<br>1.06 (0.70-1.62)	0.7624	0.8837	0.83 (0.52-1.34)<br>1.06 (0.67-1.69)	0.7054	0.8970	0.72 (0.40-1.30)<br>1.05 (0.64-1.75)	0.5622	0.7794
rs12744244 (*CRP*)	AA vs. CC<br>AC vs. CC									
rs2027471 (*CRP*)	AA vs. TT<br>AT vs. TT	0.81 (0.34-1.93)<br>0.82 (0.55-1.20)	0.5623	0.8810	0.68 (0.26-1.82)<br>0.89 (0.59-1.34)	0.6340	0.8568	0.55 (0.16-1.85)<br>0.84 (0.55-1.28)	0.4519	0.7794
rs2592887 (*CRP*)	AA vs. GG<br>AG vs. GG	0.95 (0.59-1.52)<br>0.84 (0.60-1.18)	0.5920	0.8810	0.92 (0.57-1.48)<br>0.88 (0.67-1.17)	0.7600	0.9048	0.82 (0.47-1.42)<br>0.78 (0.54-1.14)	0.5076	0.7794
rs2794520 (*CRP*)	AA vs. GG<br>AG vs. GG	0.85 (0.37-1.95)<br>0.85 (0.59-1.23)	0.6694	0.8810	0.74 (0.29-1.89)<br>0.94 (0.63-1.39)	0.7469	0.9048	0.63 (0.20-1.97)<br>0.89 (0.59-1.35)	0.5964	0.7794
rs3093075 (*CRP*)	AA vs. CC<br>AC vs. CC	0.79 (0.50-1.25)<br>1.36 (0.87-2.13)	0.1473	0.8396	0.78 (0.49-1.23)<br>1.37 (0.88-2.13)	0.1355	0.7503	0.59 (0.34-1.00)<br>1.54 (0.97-2.43)	**0.0292**	0.4157
rs1799963 (*F2*)	AG vs. GG	0.70 (0.09-5.60)	0.7294	0.8810	0.59 (0.07-4.96)	0.6125	0.8568	0.76 (0.09-6.53)	0.7947	0.8843
rs6025 (*F5*)	AA vs. GG<br>AG vs. GG	 0.74 (0.15-3.68)	0.6984	0.8810	 0.99 (0.19-5.22)	0.9917	0.9917	 1.19 (0.22-6.36)	0.8320	0.8843
rs1801274 (*FCGR2A*)	AA vs. GG<br>AG vs. GG	1.54 (0.80-2.97)<br>1.25 (0.78-1.99)	0.3139	0.8396	1.75 (0.88-3.49)<br>1.35 (0.80-2.28)	0.1862	0.7503	1.76 (0.85-3.63)<br>1.42 (0.80-2.50)	0.2088	0.6834
rs1800790 (*FGB*)	AA vs. GG<br>AG vs. GG	1.71 (0.54-5.42)<br>0.74 (0.40-1.37)	0.3655	0.8473	1.58 (0.46-5.48)<br>0.78 (0.40-1.53)	0.5306	0.8192	1.77 (0.80-3.91)<br>0.94 (0.43-2.03)	0.6718	0.8357
rs1260326 (*GCKR*)	TT vs. CC<br>TC vs. CC	0.65 (0.29-1.46)<br>1.02 (0.73-1.42)	0.5835	0.8810	0.56 (0.23-1.34)<br>0.96 (0.67-1.37)	0.4280	0.8192	0.60 (0.24-1.50)<br>0.86 (0.56-1.31)	0.4447	0.7794
rs1143623 (*IL1B*)	CC vs. GG<br>CG vs. GG	1.87 (0.33-10.57)<br>0.77 (0.51-1.15)	0.3457	0.8396	2.76 (0.41-18.58)<br>0.76 (0.49-1.18)	0.2490	0.7503	3.75 (0.98-14.38)<br>0.82 (0.55-1.21)	0.0748	0.6579
rs1800871 (*IL10*)	TT vs. CC<br>TC vs. CC	0.91 (0.55-1.52)<br>1.00 (0.71-1.41)	0.9119	0.9491	0.90 (0.50-1.60)<br>1.00 (0.67-1.50)	0.9046	0.9646	0.94 (0.54-1.66)<br>0.95 (0.60-1.51)	0.9654	0.9775
rs1800872 (*IL10*)	AA vs. CC<br>AC vs. CC	0.92 (0.55-1.55)<br>1.02 (0.72-1.43)	0.9100	0.9491	0.90 (0.50-1.63)<br>1.02 (0.68-1.53)	0.9002	0.9646	0.94 (0.53-1.68)<br>0.97 (0.62-1.53)	0.9775	0.9775
rs1800896 (*IL10*)	GG vs. AA<br>GA vs. AA	0.90 (0.57-1.42)<br>0.87 (0.60-1.27)	0.7011	0.8810	0.87 (0.51-1.46)<br>0.81 (0.57-1.16)	0.4899	0.8192	0.89 (0.48-1.63)<br>0.83 (0.57-1.20)	0.6020	0.7794
rs2243248 (*IL4*)	GG vs. TT<br>GT vs. TT	0.77 (0.25-2.36)<br>0.87 (0.58-1.30)	0.6991	0.8810	1.07 (0.32-3.56)<br>0.83 (0.55-1.25)	0.6301	0.8568	1.29 (0.36-4.58)<br>0.75 (0.47-1.19)	0.4005	0.7794
rs2243250 (*IL4*)	TT vs. CC<br>TC vs. CC	1.12 (0.57-2.23)<br>0.75 (0.36-1.60)	0.2240	0.8396	1.17 (0.56-2.46)<br>0.73 (0.33-1.61)	0.1629	0.7503	1.37 (0.62-3.03)<br>0.81 (0.32-2.04)	0.1523	0.6834
rs2243270 (*IL4*)	GG vs. AA<br>GA vs. AA	0.83 (0.46-1.50)<br>0.66 (0.41-1.06)	0.3400	0.8396	0.89 (0.47-1.69)<br>0.65 (0.40-1.05)	0.2551	0.7503	1.01 (0.52-1.96)<br>0.75 (0.43-1.31)	0.4399	0.7794
rs1801275 (*IL4R*)	GG vs. AA<br>GA vs. AA	1.15 (0.71-1.85)<br>1.01 (0.60-1.68)	0.6738	0.8810	1.08 (0.61-1.92)<br>1.05 (0.55-1.98)	0.9525	0.9777	0.96 (0.49-1.89)<br>1.08 (0.57-2.04)	0.8306	0.8843
rs1805015 (*IL4R*)	CC vs. TT<br>CT vs. TT	1.22 (0.81-1.85)<br>0.91 (0.60-1.37)	0.3949	0.8756	1.26 (0.78-2.04)<br>0.95 (0.63-1.43)	0.4729	0.8192	1.07 (0.67-1.71)<br>0.85 (0.57-1.27)	0.5156	0.7794
rs5918 (*ITGB3*)	CC vs. TT<br>CT vs. TT	0.45 (0.06-3.24)<br>1.15 (0.85-1.54)	0.4479	0.8810	0.41 (0.06-2.97)<br>1.03 (0.74-1.44)	0.5914	0.8568	0.81 (0.12-5.30)<br>1.08 (0.73-1.59)	0.8983	0.9350
rs11003125 (*MBL2*)	GG vs. CC<br>GC vs. CC	1.34 (0.38-4.72)<br>1.18 (0.77-1.82)	0.6717	0.8810	1.08 (0.35-3.35)<br>1.35 (0.88-2.06)	0.3508	0.8192	1.15 (0.37-3.60)<br>1.48 (0.84-2.58)	0.2965	0.7561
rs1800450 (*MBL2*)	AA vs. GG<br>AG vs. GG	1.77 (0.29-10.95)<br>1.59 (0.69-3.68)	0.3286	0.8396	2.20 (0.56-8.58)<br>1.66 (0.76-3.67)	0.2065	0.7503	2.88 (0.78-10.64)<br>1.63 (0.68-3.93)	0.2412	0.6834
rs1800451 (*MBL2*)	AA vs. GG<br>AG vs. GG	0.94 (0.46-1.91)<br>1.39 (0.95-2.04)	0.1707	0.8396	1.04 (0.47-2.31)<br>1.37 (0.94-1.98)	0.2391	0.7503	1.17 (0.51-2.66)<br>1.26 (0.86-1.87)	0.4844	0.7794
rs5030737 (*MBL2*)	TT vs. CC<br>TC vs. CC	 0.80 (0.19-3.28)	0.7428	0.8810	 1.04 (0.22-4.97)	0.9581	0.9777	 1.48 (0.30-7.25)	0.6113	0.7794
rs7096206 (*MBL2*)	CC vs. GG<br>CG vs. GG	0.32 (0.07-1.54)<br>0.74 (0.49-1.13)	0.1534	0.8396	0.25 (0.05-1.33)<br>0.76 (0.49-1.18)	0.1444	0.7503	0.39 (0.07-2.14)<br>0.68 (0.40-1.13)	0.1831	0.6834
rs1800482 (*NOS2A*)	CC vs. GG<br>CG vs. GG	3.74 (0.59-23.56)<br>1.16 (0.82-1.64)	0.2004	0.8396	4.12 (0.42-40.69)<br>1.10 (0.74-1.63)	0.2876	0.7568	4.03 (0.48-33.61)<br>1.29 (0.83-1.99)	0.2163	0.6834
rs9282799 (*NOS2A*)	TT vs. CC<br>TC vs. CC									
rs1799983 (*NOS3*)	TT vs. GG<br>TG vs. GG									
rs2070744 (*NOS3*)	CC vs. TT<br>CT vs. TT	1.46 (0.50-4.28)<br>0.93 (0.68-1.28)	0.6548	0.8810	1.31 (0.45-3.84)<br>0.92 (0.69-1.23)	0.7114	0.8970	1.45 (0.57-3.68)<br>0.96 (0.70-1.30)	0.6921	0.8404
rs662 (*PON1*)	GG vs. AA<br>GA vs. AA	1.37 (0.79-2.35)<br>1.54 (0.95-2.49)	0.2703	0.8396	1.52 (0.85-2.72)<br>1.58 (0.99-2.51)	0.2802	0.7568	1.32 (0.73-2.39)<br>1.51 (0.93-2.47)	0.3479	0.7794
rs854560 (*PON1*)	AA vs. TT<br>AT vs. TT	1.26 (0.50-3.17)<br>0.92 (0.57-1.49)	0.8036	0.9107	1.30 (0.57-2.97)<br>0.82 (0.49-1.37)	0.5025	0.8192	1.27 (0.57-2.82)<br>0.79 (0.46-1.35)	0.4311	0.7794
rs1801282 (*PPARG*)	GG vs. CC<br>GC vs. CC									

Variant	Genotype	Crude Model			Age-Adjusted Model			Fully Adjusted[b] Model		
		OR (95% CI)	P-value	FDR-adjusted P-value	OR (95% CI)	P-value	FDR-adjusted P-value	OR (95% CI)	P-value	FDR-adjusted P-value
rs1799762 (*SERPINE1*)	4G4G vs. 5G5G<br>4G5G vs. 5G5G	1.43 (0.80-2.58)<br>1.01 (0.72-1.43)	0.4136	0.8789	1.54 (0.74-3.21)<br>0.96 (0.66-1.40)	0.3889	0.8192	1.55 (0.75-3.22)<br>0.86 (0.60-1.23)	0.2215	0.6834
rs1800468 (*TGFB1/B9D2*)	AA vs. GG<br>AG vs. GG									
rs1800469 (*TGFB1/B9D2*)	TT vs. CC<br>TC vs. CC	0.92 (0.55-1.54)<br>1.18 (0.80-1.76)	0.4849	0.8810	0.81 (0.42-1.58)<br>1.17 (0.74-1.87)	0.5407	0.8192	0.98 (0.51-1.90)<br>1.21 (0.72-2.05)	0.5879	0.7794
rs1800470 (*TGFB1*)	CC vs. TT<br>CT vs. TT	1.28 (0.77-2.13)<br>0.95 (0.61-1.48)	0.3428	0.8396	1.28 (0.71-2.29)<br>0.95 (0.59-1.52)	0.4330	0.8192	1.52 (0.77-3.00)<br>1.08 (0.62-1.87)	0.2880	0.7561
rs4986790 (*TLR4*)	GG vs. AA<br>GA vs. AA	0.91 (0.10-8.58)<br>0.77 (0.46-1.29)	0.5918	0.8810	1.95 (0.20-19.41)<br>0.73 (0.43-1.22)	0.4083	0.8192	1.56 (0.15-15.97)<br>0.65 (0.34-1.26)	0.3839	0.7794
rs1800629 (*TNF*)	AA vs. GG<br>AG vs. GG									
rs1800750 (*TNF*)	AA vs. GG<br>AG vs. GG									
rs361525 (*TNF*)	AA vs. GG<br>AG vs. GG									
rs2239185 (*VDR*)	CC vs. TT<br>CT vs. TT	0.92 (0.61-1.39)<br>0.90 (0.62-1.31)	0.8290	0.9191	0.86 (0.56-1.33)<br>0.97 (0.64-1.45)	0.8102	0.9421	0.76 (0.48-1.18)<br>1.16 (0.76-1.77)	0.2396	0.6834
rs731236 (*VDR*)	CC vs. TT<br>CT vs. TT	1.30 (0.72-2.32)<br>1.44 (1.08-1.92)	0.0574	0.8396	1.27 (0.67-2.41)<br>1.48 (1.08-2.04)	0.0720	0.7503	1.43 (0.74-2.78)<br>1.58 (1.18-2.12)	**0.0326**	0.4157
rs890945 (Chr 5q33.3)	AA vs. TT<br>AT vs. TT	0.51 (0.28-0.96)<br>0.84 (0.56-1.27)	0.2067	0.8396	0.51 (0.27-0.95)<br>0.81 (0.52-1.27)	0.2072	0.7503	0.39 (0.17-0.86)<br>0.78 (0.52-1.18)	0.0774	0.6579
Mexican Americans										
rs1042713 (*ADRB2*)	AA vs. GG<br>AG vs. GG	1.34 (0.69-2.57)<br>0.94 (0.58-1.50)	0.3397	0.8092	1.44 (0.76-2.72)<br>0.97 (0.60-1.58)	0.2894	0.7717	1.36 (0.73-2.53)<br>0.86 (0.49-1.53)	0.2713	0.7635
rs1042714 (*ADRB2*)	GG vs. CC<br>GC vs. CC	0.73 (0.32-1.66)<br>1.15 (0.77-1.71)	0.5208	0.8728	0.69 (0.34-1.38)<br>1.14 (0.79-1.64)	0.4164	0.8514	0.78 (0.37-1.63)<br>1.19 (0.83-1.71)	0.4051	0.7635
rs429358 (*APOE*)	CC vs. TT<br>CT vs. TT	0.77 (0.24-2.51)<br>1.31 (0.88-1.96)	0.2852	0.7355	0.90 (0.31-2.58)<br>1.31 (0.88-1.95)	0.2862	0.7717	0.81 (0.23-2.84)<br>1.33 (0.92-1.91)	0.2381	0.7635
rs7412 (*APOE*)	TT vs. CC<br>TC vs. CC									
rs769214 (*CAT*)	GG vs. AA<br>GA vs. AA	1.25 (0.73-2.11)<br>1.01 (0.66-1.54)	0.4426	0.8341	1.24 (0.71-2.16)<br>1.08 (0.68-1.73)	0.5879	0.8886	1.22 (0.63-2.37)<br>1.03 (0.61-1.75)	0.5975	0.8392
rs2280788 (*CCL5*)	GG vs. CC<br>GC vs. CC	0.68 (0.11-4.17)	0.6603	0.8931	0.67 (0.11-4.11)	0.6530	0.8886	0.74 (0.12-4.38)	0.7285	0.8392
rs1799864 (*CCR2*)	AA vs. GG<br>AG vs. GG	1.30 (0.46-3.62)<br>0.95 (0.65-1.37)	0.7621	0.8931	1.48 (0.48-4.59)<br>1.01 (0.72-1.42)	0.6529	0.8886	1.59 (0.52-4.85)<br>0.97 (0.69-1.35)	0.5357	0.8203
rs1205 (*CRP*)	AA vs. GG<br>AG vs. GG	0.91 (0.47-1.76)<br>1.14 (0.81-1.60)	0.6353	0.8931	0.88 (0.42-1.84)<br>1.09 (0.78-1.53)	0.7400	0.8886	0.86 (0.40-1.86)<br>1.11 (0.75-1.63)	0.7104	0.8392
rs1417938 (*CRP*)	TT vs. AA<br>TA vs. AA	1.17 (0.49-2.80)<br>1.20 (0.90-1.60)	0.6334	0.8931	1.12 (0.46-2.74)<br>1.18 (0.90-1.55)	0.6789	0.8886	1.12 (0.45-2.81)<br>1.21 (0.91-1.61)	0.6449	0.8392
rs1800947 (*CRP*)	CC vs. GG<br>CG vs. GG	0.52 (0.16-1.70)	0.2661	0.7355	0.38 (0.10-1.36)	0.1284	0.7376	0.26 (0.06-1.14)	0.0729	0.6656
rs2808630 (*CRP*)	GG vs. AA<br>GA vs. AA	0.95 (0.39-2.29)<br>0.70 (0.37-1.30)	0.3962	0.8341	0.94 (0.38-2.32)<br>0.73 (0.39-1.34)	0.4789	0.8514	0.73 (0.33-1.62)<br>0.84 (0.45-1.56)	0.6415	0.8392
rs3091244 (*CRP*)	AA vs. CC<br>AC vs. CC<br>AT vs. CC<br>CT vs. CC<br>TT vs. CC									
rs3093058 (*CRP*)	TT vs. AA<br>TA vs. AA									
rs3093066 (*CRP*)	AA vs. CC<br>AC vs. CC	0.28 (0.06-1.26)	0.0946	0.7355	0.27 (0.06-1.18)	0.0790	0.5863	0.18 (0.03-1.26)	0.0815	0.6656
rs11265260 (*CRP*)	GG vs. AA<br>GA vs. AA									
rs12093699 (*CRP*)	AA vs. GG<br>AG vs. GG	1.14 (0.54-2.43)<br>1.06 (0.78-1.42)	0.8561	0.8931	1.15 (0.55-2.37)<br>1.01 (0.74-1.37)	0.8482	0.8886	1.02 (0.51-2.04)<br>1.01 (0.72-1.42)	0.9906	1.0000
rs12744244 (*CRP*)	AA vs. CC<br>AC vs. CC	0.67 (0.10-4.37)<br>0.77 (0.43-1.38)	0.5369	0.8728	0.66 (0.11-3.76)<br>0.74 (0.41-1.34)	0.4522	0.8514	0.71 (0.12-4.25)<br>0.69 (0.42-1.13)	0.3329	0.7635
rs2027471 (*CRP*)	AA vs. TT<br>AT vs. TT	0.92 (0.53-1.61)<br>1.11 (0.81-1.51)	0.7061	0.8931	0.91 (0.50-1.67)<br>1.05 (0.76-1.45)	0.8484	0.8886	0.87 (0.46-1.65)<br>1.06 (0.74-1.52)	0.7707	0.8392
rs2592887 (*CRP*)	AA vs. GG<br>AG vs. GG	0.97 (0.58-1.61)<br>1.04 (0.76-1.41)	0.9349	0.9349	0.94 (0.53-1.67)<br>0.97 (0.71-1.31)	0.9557	0.9557	0.95 (0.52-1.72)<br>0.99 (0.73-1.33)	0.9636	1.0000
rs2794520 (*CRP*)	AA vs. GG<br>AG vs. GG	0.91 (0.49-1.70)<br>1.08 (0.81-1.44)	0.7715	0.8931	0.88 (0.43-1.81)<br>1.03 (0.77-1.39)	0.8363	0.8886	0.87 (0.41-1.81)<br>1.07 (0.78-1.46)	0.7537	0.8392
rs3093075 (*CRP*)	AA vs. CC<br>AC vs. CC									
rs1799963 (*F2*)	AG vs. GG	1.84 (0.49-6.90)	0.3468	0.8092	1.84 (0.46-7.39)	0.3730	0.8514	2.12 (0.51-8.74)	0.2833	0.7635
rs6025 (*F5*)	AA vs. GG<br>AG vs. GG									
rs1801274 (*FCGR2A*)	AA vs. GG<br>AG vs. GG	1.45 (0.80-2.65)<br>1.11 (0.73-1.69)	0.2369	0.7355	1.40 (0.76-2.59)<br>1.02 (0.68-1.54)	0.2305	0.7376	1.37 (0.72-2.60)<br>1.02 (0.69-1.52)	0.2832	0.7635

Variant	Genotype	Crude Model			Age-Adjusted Model			Fully Adjusted[b] Model		
		**OR (95% CI)**	**P-value**	**FDR-adjusted P-value**	**OR (95% CI)**	**P-value**	**FDR-adjusted P-value**	**OR (95% CI)**	**P-value**	**FDR-adjusted P-value**
rs1800790 (*FGB*)	AA vs. GG<br>AG vs. GG	0.92 (0.19-4.55)<br>0.70 (0.40-1.24)	0.4342	0.8341	1.27 (0.27-6.04)<br>0.73 (0.42-1.27)	0.4594	0.8514	1.46 (0.26-8.36)<br>0.81 (0.42-1.54)	0.6715	0.8392
rs1260326 (*GCKR*)	TT vs. CC<br>TC vs. CC	1.40 (0.80-2.44)<br>0.82 (0.51-1.32)	0.2510	0.7355	1.33 (0.74-2.38)<br>0.84 (0.51-1.38)	0.3755	0.8514	1.38 (0.76-2.50)<br>0.96 (0.59-1.57)	0.5212	0.8203
rs1143623 (*IL1B*)	CC vs. GG<br>CG vs. GG	1.52 (1.16-1.99)<br>1.34 (0.91-1.99)	0.1526	0.7355	1.84 (1.41-2.40)<br>1.45 (0.95-2.20)	**0.0467**	0.5863	2.14 (1.63-2.81)<br>1.63 (1.08-2.45)	**0.0073**	0.3577
rs1800871 (*IL10*)	TT vs. CC<br>TC vs. CC	1.70 (1.09-2.65)<br>1.22 (0.72-2.07)	0.2020	0.7355	1.71 (1.04-2.80)<br>1.15 (0.65-2.01)	0.2157	0.7376	1.56 (0.93-2.63)<br>1.03 (0.54-1.95)	0.3443	0.7635
rs1800872 (*IL10*)	AA vs. CC<br>AC vs. CC	1.72 (1.11-2.68)<br>1.24 (0.72-2.12)	0.1920	0.7355	1.73 (1.06-2.81)<br>1.15 (0.65-2.05)	0.2075	0.7376	1.58 (0.95-2.62)<br>1.03 (0.54-1.98)	0.3384	0.7635
rs1800896 (*IL10*)	GG vs. AA<br>GA vs. AA	0.60 (0.22-1.60)<br>0.63 (0.36-1.08)	0.1927	0.7355	0.56 (0.19-1.65)<br>0.61 (0.34-1.08)	0.1845	0.7376	0.65 (0.22-1.86)<br>0.58 (0.32-1.04)	0.1856	0.6996
rs2243248 (*IL4*)	GG vs. TT<br>GT vs. TT	0.79 (0.13-4.88)<br>0.87 (0.57-1.34)	0.7753	0.8931	0.73 (0.11-4.72)<br>1.04 (0.63-1.72)	0.9023	0.9215	0.71 (0.10-4.90)<br>1.03 (0.67-1.59)	0.8666	0.9231
rs2243250 (*IL4*)	TT vs. CC<br>TC vs. CC	0.94 (0.53-1.67)<br>0.89 (0.53-1.50)	0.8334	0.8931	1.00 (0.54-1.83)<br>0.91 (0.53-1.55)	0.8400	0.8886	0.96 (0.52-1.76)<br>0.85 (0.49-1.45)	0.7319	0.8392
rs2243270 (*IL4*)	GG vs. AA<br>GA vs. AA	0.83 (0.46-1.52)<br>0.79 (0.50-1.26)	0.4783	0.8680	0.87 (0.46-1.65)<br>0.79 (0.51-1.23)	0.4716	0.8514	0.87 (0.45-1.67)<br>0.78 (0.52-1.18)	0.4753	0.8203
rs1801275 (*IL4R*)	GG vs. AA<br>GA vs. AA	1.37 (0.82-2.27)<br>0.71 (0.49-1.04)	**0.0470**	0.7355	1.56 (0.84-2.89)<br>0.73 (0.49-1.11)	0.0575	0.5863	1.46 (0.67-3.16)<br>0.74 (0.48-1.15)	0.1467	0.6758
rs1805015 (*IL4R*)	CC vs. TT<br>CT vs. TT	2.17 (0.81-5.83)<br>0.81 (0.51-1.28)	0.1712	0.7355	2.53 (0.70-9.11)<br>0.80 (0.50-1.29)	0.1873	0.7376	3.12 (0.73-13.44)<br>0.77 (0.49-1.22)	0.1123	0.6758
rs5918 (*ITGB3*)	CC vs. TT<br>CT vs. TT	2.33 (0.75-7.21)<br>1.07 (0.56-2.05)	0.4296	0.8341	2.71 (0.74-9.85)<br>1.05 (0.52-2.09)	0.3892	0.8514	4.28 (0.95-19.40)<br>0.97 (0.49-1.91)	0.1661	0.6782
rs11003125 (*MBL2*)	GG vs. CC<br>GC vs. CC	0.91 (0.46-1.80)<br>0.88 (0.49-1.58)	0.7775	0.8931	1.00 (0.50-2.00)<br>0.91 (0.51-1.62)	0.8235	0.8886	1.06 (0.52-2.17)<br>0.90 (0.50-1.60)	0.7374	0.8392
rs1800450 (*MBL2*)	AA vs. GG<br>AG vs. GG	0.84 (0.29-2.43)<br>1.31 (0.74-2.32)	0.4171	0.8341	0.73 (0.25-2.15)<br>1.21 (0.65-2.25)	0.5921	0.8886	0.80 (0.28-2.33)<br>1.41 (0.74-2.67)	0.3357	0.7635
rs1800451 (*MBL2*)	AA vs. GG<br>AG vs. GG	 0.91 (0.40-2.06)	0.8155	0.8931	 0.87 (0.34-2.22)	0.7588	0.8886	 0.81 (0.32-2.09)	0.6567	0.8392
rs5030737 (*MBL2*)	TT vs. CC<br>TC vs. CC									
rs7096206 (*MBL2*)	CC vs. GG<br>CG vs. GG	1.36 (0.22-8.34)<br>1.19 (0.77-1.83)	0.7288	0.8931	1.31 (0.18-9.28)<br>1.13 (0.66-1.95)	0.8516	0.8886	0.22 (0.04-1.18)<br>1.04 (0.56-1.96)	0.4480	0.8130
rs1800482 (*NOS2A*)	CC vs. GG<br>CG vs. GG									
rs9282799 (*NOS2A*)	TT vs. CC<br>TC vs. CC									
rs1799983 (*NOS3*)	TT vs. GG<br>TG vs. GG	1.29 (0.48-3.43)<br>0.57 (0.38-0.83)	**0.0451**	0.7355	1.00 (0.35-2.92)<br>0.52 (0.39-0.70)	**0.0325**	0.5863	1.07 (0.36-3.24)<br>0.50 (0.38-0.66)	**0.0290**	0.4737
rs2070744 (*NOS3*)	CC vs. TT<br>CT vs. TT	0.73 (0.32-1.66)<br>0.68 (0.48-0.98)	0.1324	0.7355	0.60 (0.24-1.52)<br>0.64 (0.46-0.90)	0.0747	0.5863	0.60 (0.20-1.75)<br>0.59 (0.45-0.78)	0.0620	0.6656
rs662 (*PON1*)	GG vs. AA<br>GA vs. AA	1.11 (0.65-1.89)<br>0.98 (0.71-1.36)	0.7477	0.8931	1.21 (0.72-2.04)<br>1.09 (0.79-1.51)	0.5546	0.8886	1.28 (0.63-2.60)<br>1.03 (0.64-1.64)	0.5175	0.8203
rs854560 (*PON1*)	AA vs. TT<br>AT vs. TT	0.57 (0.20-1.64)<br>0.74 (0.52-1.06)	0.1837	0.7355	0.49 (0.16-1.52)<br>0.67 (0.47-0.96)	0.0855	0.5863	0.61 (0.20-1.92)<br>0.61 (0.38-0.99)	0.1077	0.6758
rs1801282 (*PPARG*)	GG vs. CC<br>GC vs. CC	1.17 (0.35-3.95)<br>0.70 (0.50-0.98)	0.1322	0.7355	1.34 (0.43-4.21)<br>0.71 (0.48-1.05)	0.1574	0.7376	1.62 (0.50-5.23)<br>0.76 (0.51-1.12)	0.2285	0.7635
rs1799762 (*SERPINE1*)	4G4G vs. 5G5G<br>4G5G vs. 5G5G	1.12 (0.60-2.08)<br>1.00 (0.71-1.40)	0.8913	0.9099	0.98 (0.53-1.83)<br>0.90 (0.64-1.27)	0.8289	0.8886	0.87 (0.46-1.66)<br>0.77 (0.55-1.07)	0.3936	0.7635
rs1800468 (*TGFB1/B9D2*)	AA vs. GG<br>AG vs. GG									
rs1800469 (*TGFB1/B9D2*)	TT vs. CC<br>TC vs. CC	1.16 (0.75-1.80)<br>1.10 (0.70-1.74)	0.7922	0.8931	1.20 (0.77-1.87)<br>1.10 (0.68-1.79)	0.7155	0.8886	1.19 (0.72-1.95)<br>1.03 (0.59-1.81)	0.7678	0.8392
rs1800470 (*TGFB1*)	CC vs. TT<br>CT vs. TT	1.27 (0.78-2.04)<br>0.95 (0.66-1.37)	0.2336	0.7355	1.30 (0.78-2.16)<br>0.95 (0.64-1.40)	0.2121	0.7376	1.31 (0.73-2.37)<br>0.85 (0.55-1.32)	0.1356	0.6758
rs4986790 (*TLR4*)	GG vs. AA<br>GA vs. AA									
rs1800629 (*TNF*)	AA vs. GG<br>AG vs. GG	0.22 (0.03-1.92)<br>0.64 (0.36-1.14)	0.1251	0.7355	0.11 (0.01-1.01)<br>0.61 (0.34-1.09)	0.0704	0.5863	0.13 (0.01-1.22)<br>0.59 (0.37-0.92)	**0.0179**	0.4386
rs1800750 (*TNF*)	AA vs. GG<br>AG vs. GG	 1.44 (0.44-4.77)	0.5345	0.8728	 1.29 (0.38-4.38)	0.6733	0.8886	 1.00 (0.44-2.28)	1.0000	1.0000
rs361525 (*TNF*)	AA vs. GG<br>AG vs. GG	3.02 (0.11-79.81)<br>0.83 (0.34-2.04)	0.7271	0.8931	2.93 (0.24-36.09)<br>0.85 (0.32-2.21)	0.6952	0.8886	2.37 (0.17-32.79)<br>0.69 (0.38-1.27)	0.3882	0.7635
rs2239185 (*VDR*)	CC vs. TT<br>CT vs. TT	0.81 (0.53-1.25)<br>1.09 (0.70-1.68)	0.2812	0.7355	0.82 (0.50-1.34)<br>1.13 (0.72-1.77)	0.2758	0.7717	0.89 (0.48-1.65)<br>1.20 (0.80-1.81)	0.3592	0.7635
rs731236 (*VDR*)	CC vs. TT<br>CT vs. TT	0.64 (0.35-1.18)<br>1.00 (0.66-1.52)	0.5522	0.8728	0.60 (0.33-1.12)<br>1.06 (0.70-1.60)	0.4285	0.8514	0.54 (0.20-1.43)<br>0.92 (0.56-1.49)	0.5113	0.8203
rs890945 (Chr 5q33.3)	AA vs. TT<br>AT vs. TT	1.12 (0.68-1.85)<br>0.96 (0.58-1.59)	0.8566	0.8931	1.35 (0.83-2.20)<br>0.94 (0.53-1.66)	0.5773	0.8886	1.26 (0.75-2.12)<br>0.94 (0.48-1.85)	0.7183	0.8392

CI, confidence interval; FDR, false-discovery rate; OR, odds ratio. Variants with missing results had unstable statistical models.

a) Defined as urinary albumin-to-creatinine ratio (ACR) above sex-specific thresholds (≥ 17 mg/g in men and ≥ 25 mg/g in women). b) Analyses adjusted for age, alcohol consumption, educational attainment, and waist:hip ratio.

Table S5. Complete results of associations of candidate gene polymorphisms and single-threshold albuminuria[a], additive genetic model

Variant	Crude Model			Age-Sex Adjusted Model			Fully Adjusted[b] Model		
	OR (95% CI)	P-value	FDR-adjusted P-value	OR (95% CI)	P-value	FDR-adjusted P-value	OR (95% CI)	P-value	FDR-adjusted P-value
Non-Hispanic whites									
rs1042713 (*ADRB2*)	1.00 (0.77-1.31)	0.9694	1.0000	1.04 (0.81-1.32)	0.7494	0.9211	1.05 (0.79-1.41)	0.7153	0.9715
rs1042714 (*ADRB2*)	0.95 (0.77-1.17)	0.6231	0.9892	0.93 (0.74-1.17)	0.5322	0.9211	0.89 (0.66-1.20)	0.4419	0.9715
rs429358 (*APOE*)	0.69 (0.47-1.03)	0.0669	0.6214	0.73 (0.50-1.07)	0.1054	0.5930	0.69 (0.46-1.04)	0.0745	0.6200
rs7412 (*APOE*)	1.00 (0.71-1.39)	0.9822	1.0000	0.92 (0.65-1.32)	0.6534	0.9211	0.89 (0.65-1.21)	0.4368	0.9715
rs769214 (*CAT*)	0.89 (0.60-1.32)	0.5451	0.9851	0.88 (0.61-1.29)	0.5044	0.9211	0.86 (0.58-1.28)	0.4383	0.9715
rs2280788 (*CCL5*)	1.00 (0.52-1.91)	1.0000	1.0000	1.15 (0.57-2.35)	0.6831	0.9211	1.08 (0.48-2.44)	0.8527	0.9715
rs1799864 (*CCR2*)	1.15 (0.81-1.63)	0.4140	0.9274	1.18 (0.80-1.74)	0.3790	0.9053	1.18 (0.80-1.74)	0.3795	0.9613
rs1205 (*CRP*)	0.98 (0.77-1.24)	0.8292	0.9892	1.00 (0.78-1.30)	0.9686	0.9819	1.05 (0.81-1.38)	0.6857	0.9715
rs1417938 (*CRP*)	1.05 (0.75-1.46)	0.7745	0.9892	1.03 (0.74-1.42)	0.8555	0.9309	0.91 (0.63-1.32)	0.6186	0.9715
rs1800947 (*CRP*)	0.90 (0.43-1.89)	0.7683	0.9892	0.88 (0.45-1.70)	0.6812	0.9211	1.02 (0.55-1.91)	0.9391	0.9715
rs2808630 (*CRP*)	0.94 (0.66-1.36)	0.7448	0.9892	0.94 (0.66-1.33)	0.7140	0.9211	1.03 (0.72-1.46)	0.8722	0.9715
rs3091244 (*CRP*)[c]	1.02 (0.65-1.60)<br>1.08 (0.79-1.49)	0.8109	0.9892	0.98 (0.62-1.56)<br>1.08 (0.79-1.47)	0.8407	0.9309	0.88 (0.47-1.66)<br>0.96 (0.66-1.39)	0.9047	0.9715
rs3093058 (*CRP*)	3.18 (0.47-21.24)	0.2211	0.8886	4.06 (0.75-21.88)	0.0991	0.5930	3.66 (0.57-23.54)	0.1631	0.6524
rs3093066 (*CRP*)	1.00 (0.30-3.28)	0.9970	1.0000	1.11 (0.33-3.76)	0.8573	0.9309	1.44 (0.47-4.44)	0.5050	0.9715
rs11265260 (*CRP*)	0.91 (0.58-1.42)	0.6659	0.9892	0.83 (0.52-1.33)	0.4217	0.9053	0.74 (0.42-1.31)	0.2863	0.8180
rs12093699 (*CRP*)	1.08 (0.77-1.50)	0.6443	0.9892	1.06 (0.76-1.48)	0.7370	0.9211	0.96 (0.66-1.40)	0.8384	0.9715
rs12744244 (*CRP*)	1.02 (0.73-1.42)	0.9149	1.0000	0.99 (0.71-1.37)	0.9387	0.9716	0.91 (0.64-1.30)	0.5900	0.9715
rs2027471 (*CRP*)	0.92 (0.74-1.15)	0.4507	0.9274	0.95 (0.74-1.23)	0.6922	0.9211	1.01 (0.77-1.33)	0.9238	0.9715
rs2592887 (*CRP*)	0.99 (0.78-1.25)	0.9175	1.0000	1.00 (0.77-1.28)	0.9819	0.9819	1.04 (0.79-1.37)	0.7807	0.9715
rs2794520 (*CRP*)	0.98 (0.77-1.23)	0.8271	0.9892	1.01 (0.78-1.31)	0.9244	0.9716	1.05 (0.80-1.37)	0.7102	0.9715
rs3093075 (*CRP*)	0.96 (0.64-1.42)	0.8142	0.9892	0.89 (0.58-1.37)	0.5843	0.9211	0.82 (0.45-1.49)	0.4996	0.9715
rs1799963 (*F2*)	2.01 (0.85-4.72)	0.1058	0.7935	1.69 (0.66-4.30)	0.2589	0.7569	1.95 (0.73-5.22)	0.1759	0.6596
rs6025 (*F5*)	1.29 (0.72-2.32)	0.3705	0.9274	1.14 (0.64-2.05)	0.6406	0.9211	1.02 (0.52-1.98)	0.9599	0.9762
rs1801274 (*FCGR2A*)	1.05 (0.83-1.33)	0.6600	0.9892	1.07 (0.85-1.35)	0.5529	0.9211	1.08 (0.84-1.39)	0.5454	0.9715
rs1800790 (*FGB*)	0.83 (0.61-1.14)	0.2402	0.8886	0.85 (0.63-1.14)	0.2578	0.7569	0.86 (0.61-1.22)	0.3845	0.9613
rs1260326 (*GCKR*)	1.19 (0.92-1.53)	0.1774	0.8886	1.26 (0.98-1.62)	0.0681	0.5930	1.29 (1.02-1.64)	**0.0361**	0.6200
rs1143623 (*IL1B*)	1.17 (0.87-1.58)	0.2921	0.8886	1.21 (0.89-1.64)	0.2179	0.7569	1.27 (0.94-1.73)	0.1157	0.6200
rs1800871 (*IL10*)	0.96 (0.75-1.23)	0.7446	0.9892	0.98 (0.75-1.27)	0.8636	0.9309	0.98 (0.76-1.27)	0.8814	0.9715
rs1800872 (*IL10*)	0.93 (0.69-1.26)	0.6213	0.9892	0.95 (0.70-1.30)	0.7424	0.9211	0.97 (0.74-1.29)	0.8481	0.9715
rs1800896 (*IL10*)	0.91 (0.76-1.07)	0.2420	0.8886	0.87 (0.74-1.04)	0.1181	0.5930	0.87 (0.73-1.05)	0.1387	0.6402
rs2243248 (*IL4*)	0.97 (0.61-1.52)	0.8749	0.9905	1.18 (0.76-1.82)	0.4450	0.9053	0.97 (0.51-1.85)	0.9270	0.9715
rs2243250 (*IL4*)	0.83 (0.59-1.18)	0.2962	0.8886	0.80 (0.56-1.15)	0.2097	0.7569	0.80 (0.55-1.17)	0.2305	0.7897
rs2243270 (*IL4*)	0.82 (0.57-1.18)	0.2676	0.8886	0.78 (0.53-1.15)	0.2007	0.7569	0.79 (0.53-1.20)	0.2553	0.8062
rs1801275 (*IL4R*)	0.97 (0.75-1.24)	0.7922	0.9892	1.03 (0.77-1.36)	0.8499	0.9309	1.06 (0.77-1.45)	0.7114	0.9715
rs1805015 (*IL4R*)	1.00 (0.70-1.43)	0.9936	1.0000	1.04 (0.72-1.51)	0.8186	0.9309	1.07 (0.74-1.55)	0.7060	0.9715
rs5918 (*ITGB3*)	1.04 (0.80-1.34)	0.7703	0.9892	1.06 (0.79-1.41)	0.6940	0.9211	1.02 (0.78-1.33)	0.8865	0.9715
rs11003125 (*MBL2*)	1.26 (0.99-1.61)	0.0602	0.6214	1.23 (0.96-1.57)	0.0963	0.5930	1.25 (0.95-1.64)	0.1065	0.6200
rs1800450 (*MBL2*)	1.14 (0.89-1.45)	0.2873	0.8886	1.09 (0.84-1.42)	0.4975	0.9211	1.08 (0.82-1.42)	0.5648	0.9715
rs1800451 (*MBL2*)	1.52 (0.96-2.39)	0.0725	0.6214	1.46 (0.94-2.28)	0.0897	0.5930	1.50 (0.93-2.43)	0.0948	0.6200
rs5030737 (*MBL2*)	1.28 (0.71-2.32)	0.3967	0.9274	1.30 (0.70-2.40)	0.3859	0.9053	1.37 (0.76-2.49)	0.2843	0.8180
rs7096206 (*MBL2*)	0.91 (0.66-1.25)	0.5324	0.9851	0.90 (0.64-1.28)	0.5557	0.9211	0.95 (0.65-1.38)	0.7716	0.9715
rs1800482 (*NOS2A*)	2.20 (0.27-18.09)	0.4480	0.9274	1.43 (0.20-10.31)	0.7146	0.9211	1.52 (0.26-8.93)	0.6284	0.9715
rs9282799 (*NOS2A*)	1.24 (0.11-14.20)	0.8573	0.9892	0.60 (0.05-7.84)	0.6862	0.9211	0.60 (0.05-7.25)	0.6744	0.9715
rs1799983 (*NOS3*)	1.14 (0.80-1.61)	0.4520	0.9274	1.19 (0.86-1.66)	0.2860	0.7670	1.16 (0.86-1.56)	0.3283	0.8954
rs2070744 (*NOS3*)	1.06 (0.86-1.30)	0.5582	0.9851	1.09 (0.87-1.36)	0.4402	0.9053	1.08 (0.85-1.37)	0.5356	0.9715
rs662 (*PON1*)	1.10 (0.82-1.48)	0.4976	0.9631	1.17 (0.89-1.54)	0.2454	0.7569	1.29 (0.98-1.71)	0.0703	0.6200
rs854560 (*PON1*)	1.09 (0.86-1.38)	0.4566	0.9274	1.09 (0.88-1.35)	0.4163	0.9053	1.04 (0.85-1.26)	0.7162	0.9715
rs1801282 (*PPARG*)	1.23 (0.88-1.73)	0.2168	0.8886	1.29 (0.94-1.78)	0.1040	0.5930	1.37 (0.95-1.97)	0.0898	0.6200
rs1799762 (*SERPINE1*)	0.96 (0.77-1.20)	0.7405	0.9892	0.98 (0.77-1.24)	0.8678	0.9309	1.03 (0.79-1.33)	0.8307	0.9715
rs1800468 (*TGFB1/B9D2*)	1.23 (0.79-1.93)	0.3397	0.9265	1.31 (0.82-2.09)	0.2388	0.7569	1.40 (0.87-2.23)	0.1552	0.6524
rs1800469 (*TGFB1/B9D2*)	1.20 (0.92-1.56)	0.1670	0.8886	1.22 (0.95-1.56)	0.1206	0.5930	1.23 (0.94-1.59)	0.1240	0.6200
rs1800470 (*TGFB1*)	1.17 (0.88-1.54)	0.2617	0.8886	1.16 (0.89-1.50)	0.2694	0.7569	1.17 (0.89-1.54)	0.2369	0.7897
rs4986790 (*TLR4*)	1.23 (0.80-1.89)	0.3354	0.9265	1.23 (0.76-1.98)	0.3825	0.9053	1.16 (0.70-1.92)	0.5519	0.9715
rs1800629 (*TNF*)	0.88 (0.63-1.25)	0.4637	0.9274	0.89 (0.62-1.28)	0.5003	0.9211	0.92 (0.58-1.48)	0.7331	0.9715
rs1800750 (*TNF*)	3.20 (2.06-4.98)	**<0.0001**	**<0.0001**	4.10 (2.28-7.40)	**0.0001**	**0.0059**	3.60 (1.65-7.88)	**0.0025**	0.1500
rs361525 (*TNF*)	1.71 (1.18-2.47)	**0.0060**	0.1800	1.89 (1.20-2.97)	**0.0079**	0.2331	1.94 (1.17-3.22)	**0.0128**	0.3840
rs2239185 (*VDR*)	0.81 (0.67-0.97)	**0.0246**	0.4020	0.82 (0.67-1.01)	0.0568	0.5930	0.84 (0.70-1.02)	0.0817	0.6200
rs731236 (*VDR*)	1.31 (1.03-1.65)	**0.0268**	0.4020	1.29 (1.01-1.63)	**0.0398**	0.5930	1.26 (0.99-1.61)	0.0612	0.6200
rs890945 (Chr 5q33.3)	1.18 (0.86-1.61)	0.2810	0.8886	1.24 (0.89-1.73)	0.1859	0.7569	1.12 (0.82-1.53)	0.4630	0.9715

Variant	Crude Model			Age-Sex Adjusted Model			Fully Adjusted[b] Model		
	OR (95% CI)	P-value	FDR-adjusted P-value	OR (95% CI)	P-value	FDR-adjusted P-value	OR (95% CI)	P-value	FDR-adjusted P-value
Non-Hispanic blacks									
rs1042713 (*ADRB2*)	0.96 (0.72-1.28)	0.7734	0.9544	1.00 (0.72-1.38)	0.9887	1.0000	1.00 (0.69-1.45)	0.9896	0.9896
rs1042714 (*ADRB2*)	0.96 (0.72-1.28)	0.7508	0.9467	0.90 (0.64-1.26)	0.5225	0.8466	0.91 (0.60-1.38)	0.6322	0.9720
rs429358 (*APOE*)	1.19 (0.82-1.71)	0.3430	0.9048	1.29 (0.91-1.81)	0.1429	0.6788	1.20 (0.86-1.68)	0.2625	0.8861
rs7412 (*APOE*)	1.21 (0.74-1.95)	0.4321	0.9048	1.13 (0.73-1.76)	0.5748	0.8466	1.22 (0.80-1.85)	0.3361	0.8861
rs769214 (*CAT*)	0.85 (0.72-1.00)	0.0506	0.5942	0.84 (0.70-1.00)	0.0531	0.5477	0.88 (0.71-1.09)	0.2370	0.8591
rs2280788 (*CCL5*)									
rs1799864 (*CCR2*)	0.88 (0.63-1.23)	0.4403	0.9048	0.90 (0.65-1.24)	0.4981	0.8466	0.84 (0.60-1.17)	0.2859	0.8861
rs1205 (*CRP*)	0.75 (0.60-0.95)	**0.0195**	0.5655	0.75 (0.58-0.98)	**0.0338**	0.5477	0.74 (0.56-0.98)	**0.0394**	0.7760
rs1417938 (*CRP*)	1.07 (0.73-1.57)	0.7358	0.9467	1.14 (0.71-1.83)	0.5787	0.8466	1.38 (0.83-2.30)	0.2005	0.8591
rs1800947 (*CRP*)	0.94 (0.23-3.79)	0.9313	0.9721	0.71 (0.18-2.75)	0.6075	0.8466	0.82 (0.22-2.97)	0.7480	0.9896
rs2808630 (*CRP*)	1.20 (0.98-1.48)	0.0771	0.5942	1.13 (0.93-1.37)	0.2012	0.7760	1.09 (0.91-1.29)	0.3291	0.8861
rs3091244 (*CRP*)[c]	1.14 (0.78-1.67)<br>0.96 (0.66-1.38)	0.6162	0.9048	1.16 (0.84-1.62)<br>1.01 (0.70-1.45)	0.6194	0.8466	1.14 (0.78-1.66)<br>1.08 (0.72-1.62)	0.7794	0.9896
rs3093058 (*CRP*)	0.74 (0.54-1.01)	0.0584	0.5942	0.77 (0.57-1.04)	0.0852	0.5477	0.72 (0.52-0.99)	**0.0466**	0.7760
rs3093066 (*CRP*)	1.26 (0.90-1.78)	0.1709	0.6195	1.31 (0.96-1.80)	0.0875	0.5477	1.19 (0.82-1.73)	0.3542	0.8932
rs11265260 (*CRP*)	1.11 (0.70-1.76)	0.6549	0.9048	1.12 (0.67-1.88)	0.6484	0.8466	1.19 (0.67-2.11)	0.5314	0.9173
rs12093699 (*CRP*)	1.03 (0.77-1.37)	0.8426	0.9721	1.02 (0.76-1.37)	0.8980	1.0000	0.97 (0.67-1.39)	0.8491	0.9896
rs12744244 (*CRP*)	0.80 (0.38-1.69)	0.5460	0.9048	0.83 (0.37-1.88)	0.6464	0.8466	1.01 (0.43-2.39)	0.9865	0.9896
rs2027471 (*CRP*)	0.78 (0.61-1.00)	0.0510	0.5942	0.79 (0.59-1.04)	0.0928	0.5477	0.77 (0.56-1.05)	0.0942	0.8135
rs2592887 (*CRP*)	1.01 (0.75-1.34)	0.9577	0.9721	1.00 (0.76-1.32)	1.0000	1.0000	0.94 (0.68-1.28)	0.6614	0.9836
rs2794520 (*CRP*)	0.83 (0.66-1.05)	0.1132	0.6195	0.84 (0.64-1.10)	0.2042	0.7760	0.83 (0.62-1.12)	0.2203	0.8591
rs3093075 (*CRP*)	1.14 (0.83-1.56)	0.4050	0.9048	1.13 (0.86-1.50)	0.3657	0.8466	1.11 (0.79-1.54)	0.5377	0.9173
rs1799963 (*F2*)									
rs6025 (*F5*)	1.05 (0.20-5.38)	0.9521	0.9721	1.51 (0.27-8.39)	0.6234	0.8466	1.79 (0.33-9.71)	0.4831	0.9173
rs1801274 (*FCGR2A*)	1.32 (0.89-1.97)	0.1637	0.6195	1.41 (0.94-2.12)	0.0966	0.5477	1.37 (0.90-2.08)	0.1355	0.8369
rs1800790 (*FGB*)	0.83 (0.47-1.46)	0.5014	0.9048	0.86 (0.46-1.60)	0.6124	0.8466	1.06 (0.56-2.02)	0.8511	0.9896
rs1260326 (*GCKR*)	0.80 (0.57-1.11)	0.1702	0.6195	0.74 (0.51-1.07)	0.1057	0.5477	0.71 (0.46-1.09)	0.1122	0.8135
rs1143623 (*IL1B*)	0.87 (0.58-1.32)	0.5076	0.9048	0.91 (0.57-1.43)	0.6535	0.8466	1.05 (0.69-1.61)	0.8101	0.9896
rs1800871 (*IL10*)	0.94 (0.71-1.24)	0.6552	0.9048	0.93 (0.68-1.26)	0.6254	0.8466	0.98 (0.71-1.35)	0.8792	0.9896
rs1800872 (*IL10*)	0.93 (0.70-1.24)	0.5918	0.9048	0.91 (0.66-1.25)	0.5513	0.8466	0.95 (0.69-1.33)	0.7682	0.9896
rs1800896 (*IL10*)	0.91 (0.69-1.21)	0.5024	0.9048	0.88 (0.65-1.19)	0.3941	0.8466	0.87 (0.62-1.22)	0.3969	0.9173
rs2243248 (*IL4*)	1.11 (0.80-1.54)	0.5036	0.9048	1.17 (0.85-1.63)	0.3219	0.8466	1.09 (0.74-1.62)	0.6368	0.9720
rs2243250 (*IL4*)	1.20 (0.81-1.79)	0.3542	0.9048	1.24 (0.80-1.93)	0.3258	0.8466	1.39 (0.88-2.20)	0.1443	0.8369
rs2243270 (*IL4*)	0.98 (0.64-1.52)	0.9410	0.9721	1.03 (0.65-1.64)	0.8919	1.0000	1.07 (0.65-1.76)	0.7804	0.9896
rs1801275 (*IL4R*)	1.17 (0.93-1.47)	0.1643	0.6195	1.11 (0.87-1.41)	0.3957	0.8466	1.02 (0.77-1.36)	0.8591	0.9896
rs1805015 (*IL4R*)	1.05 (0.87-1.27)	0.6053	0.9048	1.06 (0.87-1.30)	0.5261	0.8466	0.93 (0.76-1.14)	0.4598	0.9173
rs5918 (*ITGB3*)	1.22 (0.92-1.62)	0.1654	0.6195	1.12 (0.82-1.54)	0.4435	0.8466	1.28 (0.87-1.89)	0.1974	0.8591
rs11003125 (*MBL2*)	0.88 (0.54-1.41)	0.5685	0.9048	0.89 (0.57-1.38)	0.5835	0.8466	0.89 (0.56-1.41)	0.6067	0.9720
rs1800450 (*MBL2*)	1.25 (0.56-2.77)	0.5747	0.9048	1.26 (0.60-2.63)	0.5276	0.8466	1.49 (0.67-3.30)	0.3080	0.8861
rs1800451 (*MBL2*)	1.25 (0.98-1.61)	0.0699	0.5942	1.30 (0.99-1.70)	0.0584	0.5477	1.26 (0.95-1.69)	0.1073	0.8135
rs5030737 (*MBL2*)	0.68 (0.13-3.50)	0.6281	0.9048	0.90 (0.15-5.44)	0.9045	1.0000	1.22 (0.21-7.25)	0.8156	0.9896
rs7096206 (*MBL2*)	0.72 (0.48-1.06)	0.0922	0.5942	0.72 (0.51-1.00)	0.0530	0.5477	0.68 (0.45-1.03)	0.0669	0.7760
rs1800482 (*NOS2A*)	1.19 (0.76-1.84)	0.4340	0.9048	1.13 (0.69-1.84)	0.6133	0.8466	1.32 (0.83-2.10)	0.2289	0.8591
rs9282799 (*NOS2A*)	0.68 (0.36-1.30)	0.2328	0.7501	0.81 (0.41-1.62)	0.5421	0.8466	0.71 (0.35-1.44)	0.3267	0.8861
rs1799983 (*NOS3*)	1.02 (0.74-1.40)	0.8968	0.9721	1.00 (0.69-1.44)	1.0000	1.0000	0.99 (0.63-1.56)	0.9646	0.9896
rs2070744 (*NOS3*)	1.02 (0.68-1.52)	0.9231	0.9721	1.00 (0.71-1.42)	0.9780	1.0000	0.98 (0.70-1.38)	0.9158	0.9896
rs662 (*PON1*)	0.94 (0.75-1.16)	0.5389	0.9048	0.99 (0.78-1.26)	0.9178	1.0000	0.90 (0.67-1.20)	0.4568	0.9173
rs854560 (*PON1*)	0.99 (0.64-1.53)	0.9721	0.9721	0.92 (0.59-1.45)	0.7095	0.8803	0.86 (0.51-1.46)	0.5606	0.9290
rs1801282 (*PPARG*)	0.75 (0.32-1.75)	0.4908	0.9048	0.85 (0.35-2.05)	0.7104	0.8803	0.98 (0.39-2.47)	0.9597	0.9896
rs1799762 (*SERPINE1*)	0.94 (0.67-1.31)	0.6936	0.9356	0.91 (0.66-1.27)	0.5691	0.8466	0.88 (0.62-1.26)	0.4760	0.9173
rs1800468 (*TGFB1/B9D2*)	0.22 (0.06-0.76)	**0.0193**	0.5655	0.21 (0.06-0.78)	**0.0224**	0.5477	0.12 (0.01-0.99)	**0.0493**	0.7760
rs1800469 (*TGFB1/B9D2*)	0.87 (0.63-1.21)	0.3963	0.9048	0.82 (0.56-1.20)	0.2896	0.8466	0.87 (0.57-1.32)	0.4928	0.9173
rs1800470 (*TGFB1*)	1.02 (0.74-1.40)	0.9214	0.9721	1.00 (0.70-1.43)	0.9910	1.0000	1.07 (0.73-1.57)	0.7087	0.9896
rs4986790 (*TLR4*)	0.96 (0.51-1.80)	0.8975	0.9721	0.99 (0.52-1.88)	0.9631	1.0000	0.93 (0.40-2.16)	0.8512	0.9896
rs1800629 (*TNF*)	0.73 (0.50-1.05)	0.0830	0.5942	0.77 (0.51-1.14)	0.1804	0.7760	0.86 (0.56-1.32)	0.4745	0.9173
rs1800750 (*TNF*)	0.87 (0.38-2.02)	0.7409	0.9467	0.97 (0.37-2.54)	0.9450	1.0000	0.98 (0.36-2.66)	0.9646	0.9896
rs361525 (*TNF*)	0.72 (0.36-1.47)	0.3566	0.9048	0.78 (0.36-1.70)	0.5103	0.8466	0.78 (0.36-1.68)	0.5102	0.9173
rs2239185 (*VDR*)	1.08 (0.86-1.36)	0.4920	0.9048	1.07 (0.84-1.36)	0.5550	0.8466	1.09 (0.87-1.35)	0.4499	0.9173
rs731236 (*VDR*)	1.14 (0.92-1.40)	0.2209	0.7501	1.13 (0.90-1.41)	0.2903	0.8466	1.16 (0.90-1.50)	0.2318	0.8591
rs890945 (Chr 5q33.3)	0.80 (0.59-1.07)	0.1242	0.6195	0.79 (0.59-1.04)	0.0884	0.5477	0.74 (0.54-1.01)	0.0593	0.7760

Variant	Crude Model			Age-Sex Adjusted Model			Fully Adjusted[b] Model		
	OR (95% CI)	P-value	FDR-adjusted P-value	OR (95% CI)	P-value	FDR-adjusted P-value	OR (95% CI)	P-value	FDR-adjusted P-value
Mexican Americans									
rs1042713 (*ADRB2*)	0.99 (0.73-1.34)	0.9411	0.9576	1.02 (0.77-1.36)	0.8639	0.9876	0.98 (0.73-1.32)	0.9006	0.9968
rs1042714 (*ADRB2*)	1.06 (0.76-1.47)	0.7371	0.9350	1.04 (0.78-1.39)	0.7772	0.9876	1.13 (0.85-1.50)	0.3799	0.9181
rs429358 (*APOE*)	1.17 (0.88-1.55)	0.2774	0.6914	1.18 (0.88-1.57)	0.2531	0.7593	1.19 (0.87-1.62)	0.2644	0.8140
rs7412 (*APOE*)	0.75 (0.44-1.28)	0.2787	0.6914	0.77 (0.40-1.50)	0.4307	0.8566	0.43 (0.18-1.06)	0.0659	0.5460
rs769214 (*CAT*)	1.16 (0.83-1.62)	0.3702	0.7854	1.16 (0.83-1.62)	0.3587	0.8566	1.12 (0.76-1.67)	0.5516	0.9695
rs2280788 (*CCL5*)	0.89 (0.16-5.07)	0.8891	0.9550	0.84 (0.14-5.13)	0.8393	0.9876	1.08 (0.17-7.02)	0.9353	0.9968
rs1799864 (*CCR2*)	1.08 (0.72-1.64)	0.6861	0.9350	1.16 (0.78-1.73)	0.4358	0.8566	1.18 (0.76-1.82)	0.4394	0.9577
rs1205 (*CRP*)	1.05 (0.74-1.49)	0.7653	0.9350	1.02 (0.71-1.48)	0.8929	0.9876	0.99 (0.68-1.44)	0.9730	0.9968
rs1417938 (*CRP*)	1.14 (0.79-1.63)	0.4662	0.8450	1.11 (0.78-1.59)	0.5371	0.9673	1.10 (0.77-1.58)	0.5693	0.9712
rs1800947 (*CRP*)	0.37 (0.08-1.77)	0.2022	0.6802	0.23 (0.04-1.28)	0.0905	0.4831	0.08 (0.03-0.20)	**<0.0001**	**<0.0001**
rs2808630 (*CRP*)	0.83 (0.58-1.21)	0.3228	0.7201	0.86 (0.61-1.23)	0.3992	0.8566	0.94 (0.68-1.31)	0.7202	0.9968
rs3091244 (*CRP*)[c]	0.38 (0.13-1.07)<br>1.09 (0.80-1.48)	0.1324	0.6802	0.41 (0.15-1.15)<br>1.06 (0.80-1.41)	0.1748	0.6002	0.41 (0.14-1.27)<br>1.07 (0.80-1.45)	0.2034	0.6940
rs3093058 (*CRP*)	1.16 (0.39-3.41)	0.7820	0.9350	1.13 (0.29-4.49)	0.8540	0.9876	1.50 (0.43-5.28)	0.5090	0.9577
rs3093066 (*CRP*)	0.37 (0.08-1.63)	0.1794	0.6802	0.35 (0.08-1.53)	0.1534	0.5829	0.23 (0.03-1.57)	0.1263	0.6105
rs11265260 (*CRP*)	0.32 (0.10-1.01)	0.0514	0.5319	0.37 (0.11-1.24)	0.1017	0.4831	0.42 (0.11-1.53)	0.1778	0.6815
rs12093699 (*CRP*)	1.05 (0.78-1.40)	0.7448	0.9350	1.03 (0.78-1.36)	0.8124	0.9876	1.00 (0.75-1.32)	0.9737	0.9968
rs12744244 (*CRP*)	0.95 (0.55-1.63)	0.8451	0.9550	0.94 (0.54-1.61)	0.8030	0.9876	0.95 (0.61-1.47)	0.8050	0.9968
rs2027471 (*CRP*)	1.02 (0.76-1.39)	0.8755	0.9550	1.00 (0.73-1.37)	0.9827	0.9876	0.95 (0.69-1.29)	0.7123	0.9968
rs2592887 (*CRP*)	1.06 (0.80-1.41)	0.6699	0.9350	1.03 (0.76-1.40)	0.8292	0.9876	1.01 (0.74-1.37)	0.9496	0.9968
rs2794520 (*CRP*)	1.03 (0.76-1.41)	0.8330	0.9550	1.01 (0.72-1.40)	0.9747	0.9876	0.98 (0.71-1.35)	0.8955	0.9968
rs3093075 (*CRP*)	0.37 (0.13-1.07)	0.0642	0.5319	0.40 (0.13-1.21)	0.0991	0.4831	0.39 (0.11-1.32)	0.1228	0.6105
rs1799963 (*F2*)	2.43 (0.67-8.84)	0.1676	0.6802	2.39 (0.56-10.17)	0.2245	0.7109	2.78 (0.69-11.27)	0.1439	0.6420
rs6025 (*F5*)	1.05 (0.25-4.38)	0.9399	0.9576	0.97 (0.25-3.73)	0.9658	0.9876	1.09 (0.23-5.07)	0.9086	0.9968
rs1801274 (*FCGR2A*)	1.31 (0.95-1.80)	0.0963	0.6067	1.29 (0.92-1.82)	0.1339	0.5733	1.29 (0.89-1.86)	0.1666	0.6815
rs1800790 (*FGB*)	0.79 (0.54-1.15)	0.2111	0.6802	0.86 (0.59-1.26)	0.4211	0.8566	0.92 (0.56-1.53)	0.7436	0.9968
rs1260326 (*GCKR*)	1.17 (0.87-1.56)	0.2861	0.6914	1.16 (0.88-1.54)	0.2840	0.8094	1.19 (0.91-1.55)	0.1880	0.6815
rs1143623 (*IL1B*)	1.30 (1.06-1.60)	**0.0149**	0.4321	1.47 (1.22-1.78)	**0.0004**	**0.0171**	1.55 (1.28-1.87)	**0.0001**	**0.0029**
rs1800871 (*IL10*)	1.24 (1.02-1.51)	**0.0361**	0.5234	1.23 (0.96-1.57)	0.0917	0.4831	1.14 (0.84-1.55)	0.3759	0.9181
rs1800872 (*IL10*)	1.25 (1.03-1.52)	**0.0290**	0.5234	1.24 (0.97-1.58)	0.0821	0.4831	1.15 (0.84-1.56)	0.3630	0.9181
rs1800896 (*IL10*)	0.74 (0.49-1.14)	0.1609	0.6802	0.72 (0.44-1.17)	0.1790	0.6002	0.75 (0.43-1.29)	0.2807	0.8140
rs2243248 (*IL4*)	0.93 (0.63-1.37)	0.6968	0.9350	1.05 (0.70-1.57)	0.8030	0.9876	1.00 (0.69-1.45)	0.9940	0.9968
rs2243250 (*IL4*)	0.96 (0.71-1.30)	0.7899	0.9350	1.00 (0.73-1.37)	0.9876	0.9876	0.94 (0.70-1.28)	0.7012	0.9968
rs2243270 (*IL4*)	0.92 (0.67-1.27)	0.5938	0.9350	0.95 (0.69-1.32)	0.7516	0.9876	0.89 (0.64-1.24)	0.4746	0.9577
rs1801275 (*IL4R*)	0.99 (0.67-1.48)	0.9775	0.9775	1.06 (0.67-1.68)	0.7918	0.9876	1.06 (0.62-1.80)	0.8369	0.9968
rs1805015 (*IL4R*)	1.13 (0.75-1.69)	0.5444	0.9045	1.16 (0.73-1.86)	0.5111	0.9673	1.23 (0.65-2.32)	0.5119	0.9577
rs5918 (*ITGB3*)	1.21 (0.75-1.94)	0.4158	0.7854	1.22 (0.73-2.06)	0.4282	0.8566	1.29 (0.72-2.31)	0.3730	0.9181
rs11003125 (*MBL2*)	0.91 (0.65-1.26)	0.5458	0.9045	0.95 (0.68-1.34)	0.7684	0.9876	0.95 (0.65-1.40)	0.7965	0.9968
rs1800450 (*MBL2*)	1.13 (0.68-1.87)	0.6174	0.9350	1.04 (0.61-1.79)	0.8746	0.9876	1.12 (0.64-1.97)	0.6732	0.9968
rs1800451 (*MBL2*)	1.16 (0.51-2.65)	0.7186	0.9350	1.10 (0.41-2.98)	0.8429	0.9876	1.05 (0.38-2.92)	0.9153	0.9968
rs5030737 (*MBL2*)	2.33 (0.87-6.21)	0.0882	0.6067	2.62 (0.82-8.36)	0.0991	0.4831	2.70 (0.85-8.63)	0.0901	0.6105
rs7096206 (*MBL2*)	1.17 (0.79-1.74)	0.4198	0.7854	1.12 (0.68-1.85)	0.6440	0.9876	1.04 (0.62-1.72)	0.8882	0.9968
rs1800482 (*NOS2A*)									
rs9282799 (*NOS2A*)									
rs1799983 (*NOS3*)	0.65 (0.39-1.10)	0.1046	0.6067	0.59 (0.37-0.93)	**0.0256**	0.3648	0.60 (0.37-0.98)	**0.0423**	0.4089
rs2070744 (*NOS3*)	0.66 (0.51-0.85)	**0.0028**	0.1624	0.61 (0.47-0.79)	**0.0006**	**0.0171**	0.62 (0.48-0.80)	**0.0008**	**0.0155**
rs662 (*PON1*)	1.07 (0.80-1.44)	0.6347	0.9350	1.12 (0.86-1.48)	0.3828	0.8566	1.14 (0.78-1.68)	0.4795	0.9577
rs854560 (*PON1*)	0.81 (0.57-1.16)	0.2329	0.6914	0.74 (0.52-1.06)	0.0960	0.4831	0.77 (0.47-1.25)	0.2724	0.8140
rs1801282 (*PPARG*)	0.81 (0.55-1.18)	0.2626	0.6914	0.83 (0.55-1.26)	0.3676	0.8566	0.91 (0.61-1.37)	0.6427	0.9968
rs1799762 (*SERPINE1*)	1.09 (0.83-1.42)	0.5313	0.9045	1.00 (0.77-1.30)	0.9794	0.9876	0.94 (0.71-1.24)	0.6337	0.9968
rs1800468 (*TGFB1/B9D2*)	0.75 (0.50-1.13)	0.1588	0.6802	0.64 (0.44-0.93)	**0.0228**	0.3648	0.70 (0.45-1.09)	0.1116	0.6105
rs1800469 (*TGFB1/B9D2*)	0.92 (0.77-1.11)	0.3903	0.7854	0.93 (0.74-1.18)	0.5498	0.9673	0.93 (0.67-1.28)	0.6301	0.9968
rs1800470 (*TGFB1*)	0.98 (0.75-1.28)	0.8690	0.9550	0.99 (0.73-1.33)	0.9367	0.9876	1.00 (0.67-1.49)	0.9880	0.9968
rs4986790 (*TLR4*)	0.54 (0.21-1.40)	0.1959	0.6802	0.50 (0.20-1.28)	0.1408	0.5733	0.45 (0.17-1.21)	0.1087	0.6105
rs1800629 (*TNF*)	0.75 (0.45-1.27)	0.2729	0.6914	0.67 (0.42-1.07)	0.0901	0.4831	0.65 (0.43-0.98)	**0.0414**	0.4089
rs1800750 (*TNF*)	1.52 (0.54-4.27)	0.4062	0.7854	1.36 (0.46-3.98)	0.5600	0.9673	1.34 (0.50-3.59)	0.5402	0.9695
rs361525 (*TNF*)	1.04 (0.48-2.26)	0.9198	0.9576	1.05 (0.47-2.35)	0.8958	0.9876	1.01 (0.50-2.02)	0.9795	0.9968
rs2239185 (*VDR*)	0.88 (0.67-1.14)	0.3092	0.7173	0.88 (0.65-1.19)	0.3765	0.8566	0.88 (0.63-1.25)	0.4662	0.9577
rs731236 (*VDR*)	0.92 (0.65-1.30)	0.6089	0.9350	0.94 (0.66-1.33)	0.6997	0.9876	0.86 (0.57-1.30)	0.4684	0.9577
rs890945 (Chr 5q33.3)	0.95 (0.66-1.36)	0.7700	0.9350	0.99 (0.66-1.48)	0.9411	0.9876	1.00 (0.62-1.62)	0.9968	0.9968

CI, confidence interval; FDR, false-discovery rate; OR, odds ratio. Variants with missing results had unstable statistical models.

a) Defined as urinary albumin-to-creatinine ratio (ACR) above ≥ 30 mg/g regardless of gender. b) Analyses adjusted for age, sex, alcohol consumption, educational attainment, and waist:hip ratio. c) For this tri-allelic variant, the first beta coefficient corresponds to the A allele, while the second corresponds to the T allele. (The C allele is the reference.) The unadjusted and FDR-adjusted P values are for the overall test of association.

Table S6. Complete results of associations of candidate gene polymorphisms and single-threshold albuminuria[a], codominant genetic model

Variant	Genotype	Crude Model			Age-Sex Adjusted Model			Fully Adjusted[b] Model		
		OR (95% CI)	P-value	FDR-adjusted P-value	OR (95% CI)	P-value	FDR-adjusted P-value	OR (95% CI)	P-value	FDR-adjusted P-value
Non-Hispanic whites										
rs1042713 (*ADRB2*)	AA vs. GG<br>AG vs. GG	1.01 (0.58-1.77)<br>1.00 (0.72-1.39)	0.9970	0.9970	1.08 (0.65-1.82)<br>1.03 (0.73-1.45)	0.9425	0.9606	1.09 (0.60-2.01)<br>1.08 (0.70-1.66)	0.9174	0.9702
rs1042714 (*ADRB2*)	GG vs. CC<br>GC vs. CC	0.88 (0.59-1.32)<br>1.00 (0.69-1.46)	0.7941	0.9706	0.87 (0.56-1.34)<br>0.94 (0.65-1.36)	0.7741	0.8729	0.77 (0.42-1.40)<br>0.97 (0.64-1.46)	0.5598	0.8170
rs429358 (*APOE*)	CC vs. TT<br>CT vs. TT	0.36 (0.14-0.95)<br>0.72 (0.47-1.10)	0.0884	0.8103	0.41 (0.16-1.04)<br>0.76 (0.50-1.14)	0.1318	0.8154	0.38 (0.14-1.06)<br>0.71 (0.46-1.10)	0.0944	0.7270
rs7412 (*APOE*)	TT vs. CC<br>TC vs. CC									
rs769214 (*CAT*)	GG vs. AA<br>GA vs. AA	0.92 (0.39-2.18)<br>0.77 (0.50-1.19)	0.5587	0.9151	0.92 (0.39-2.17)<br>0.76 (0.50-1.13)	0.4988	0.8154	0.90 (0.38-2.15)<br>0.71 (0.48-1.05)	0.3614	0.8065
rs2280788 (*CCL5*)	GG vs. CC<br>GC vs. CC	3.10 (0.55-17.31)<br>0.86 (0.44-1.70)	0.4281	0.9151	3.33 (0.28-39.55)<br>1.01 (0.49-2.08)	0.5852	0.8154	4.90 (0.14-169.04)<br>0.89 (0.41-1.92)	0.5227	0.8065
rs1799864 (*CCR2*)	AA vs. GG<br>AG vs. GG	0.86 (0.21-3.48)<br>1.24 (0.81-1.87)	0.5286	0.9151	1.11 (0.29-4.19)<br>1.23 (0.77-1.94)	0.5838	0.8154	0.46 (0.10-2.12)<br>1.33 (0.83-2.12)	0.2393	0.7601
rs1205 (*CRP*)	AA vs. GG<br>AG vs. GG	1.02 (0.66-1.58)<br>0.91 (0.61-1.34)	0.7891	0.9706	1.14 (0.73-1.78)<br>0.89 (0.60-1.33)	0.5468	0.8154	1.25 (0.78-2.02)<br>0.92 (0.61-1.38)	0.4700	0.8065
rs1417938 (*CRP*)	TT vs. AA<br>TA vs. AA	1.24 (0.65-2.35)<br>0.94 (0.60-1.49)	0.7115	0.9317	1.21 (0.64-2.29)<br>0.92 (0.58-1.44)	0.6842	0.8420	0.92 (0.42-1.99)<br>0.85 (0.53-1.39)	0.7782	0.8661
rs1800947 (*CRP*)	CC vs. GG<br>CG vs. GG									
rs2808630 (*CRP*)	GG vs. AA<br>GA vs. AA	1.03 (0.53-2.02)<br>0.86 (0.54-1.36)	0.6706	0.9151	1.09 (0.60-1.99)<br>0.82 (0.52-1.29)	0.4826	0.8154	1.26 (0.71-2.25)<br>0.92 (0.56-1.51)	0.6061	0.8613
rs3091244 (*CRP*)	AA vs. CC<br>AC vs. CC<br>AT vs. CC<br>CT vs. CC<br>TT vs. CC	1.13 (0.09-13.60)<br>0.91 (0.45-1.85)<br>1.16 (0.57-2.37)<br>0.96 (0.58-1.58)<br>1.26 (0.67-2.38)	0.9058	0.9774	0.87 (0.07-11.10)<br>0.85 (0.40-1.81)<br>1.20 (0.61-2.36)<br>0.92 (0.56-1.52)<br>1.25 (0.68-2.29)	0.8603	0.9119	0.71 (0.04-11.20)<br>0.77 (0.30-1.97)<br>1.01 (0.44-2.33)<br>0.87 (0.53-1.43)<br>0.96 (0.44-2.11)	0.9588	0.9702
rs3093058 (*CRP*)	TT vs. AA<br>TA vs. AA	 3.18 (0.47-21.24)	0.2211	0.9151	 4.06 (0.75-21.88)	0.0991	0.8154	 3.66 (0.57-23.54)	0.1631	0.7270
rs3093066 (*CRP*)	AA vs. CC<br>AC vs. CC	 1.00 (0.30-3.28)	0.9970	0.9970	 1.11 (0.33-3.76)	0.8573	0.9119	 1.44 (0.47-4.44)	0.5050	0.8065
rs11265260 (*CRP*)	GG vs. AA<br>GA vs. AA									
rs12093699 (*CRP*)	AA vs. GG<br>AG vs. GG	1.21 (0.62-2.37)<br>1.04 (0.61-1.78)	0.8673	0.9774	1.19 (0.60-2.35)<br>1.00 (0.59-1.70)	0.8801	0.9146	0.95 (0.41-2.20)<br>0.94 (0.55-1.61)	0.9702	0.9702
rs12744244 (*CRP*)	AA vs. CC<br>AC vs. CC	0.66 (0.19-2.29)<br>1.15 (0.71-1.86)	0.6246	0.9151	0.69 (0.19-2.43)<br>1.08 (0.67-1.74)	0.7556	0.8706	0.35 (0.10-1.29)<br>1.08 (0.69-1.68)	0.3089	0.8065
rs2027471 (*CRP*)	AA vs. TT<br>AT vs. TT	0.94 (0.60-1.47)<br>0.83 (0.55-1.25)	0.5638	0.9151	1.04 (0.66-1.65)<br>0.83 (0.54-1.28)	0.5121	0.8154	1.18 (0.73-1.91)<br>0.87 (0.56-1.35)	0.4894	0.8065
rs2592887 (*CRP*)	AA vs. GG<br>AG vs. GG	0.95 (0.59-1.53)<br>1.04 (0.70-1.55)	0.9063	0.9774	0.98 (0.60-1.60)<br>1.03 (0.68-1.57)	0.9658	0.9658	1.07 (0.63-1.81)<br>1.05 (0.65-1.69)	0.9400	0.9702
rs2794520 (*CRP*)	AA vs. GG<br>AG vs. GG	1.05 (0.65-1.69)<br>0.88 (0.59-1.30)	0.6822	0.9151	1.19 (0.73-1.94)<br>0.86 (0.58-1.29)	0.4441	0.8154	1.25 (0.77-2.03)<br>0.91 (0.60-1.39)	0.5069	0.8065
rs3093075 (*CRP*)	AA vs. CC<br>AC vs. CC	1.12 (0.10-12.05)<br>0.94 (0.62-1.42)	0.9550	0.9910	0.88 (0.08-10.17)<br>0.88 (0.56-1.39)	0.8585	0.9119	0.74 (0.05-10.41)<br>0.81 (0.46-1.44)	0.7035	0.8661
rs1799963 (*F2*)	AG vs. GG	2.01 (0.85-4.72)	0.1058	0.8313	1.69 (0.66-4.30)	0.2589	0.8154	1.95 (0.73-5.22)	0.1759	0.7270
rs6025 (*F5*)	AA vs. GG<br>AG vs. GG									
rs1801274 (*FCGR2A*)	AA vs. GG<br>AG vs. GG	1.11 (0.70-1.75)<br>1.05 (0.79-1.40)	0.8525	0.9774	1.15 (0.71-1.86)<br>1.18 (0.85-1.64)	0.6644	0.8420	1.17 (0.70-1.96)<br>1.18 (0.89-1.56)	0.6441	0.8661
rs1800790 (*FGB*)	AA vs. GG<br>AG vs. GG	0.84 (0.29-2.39)<br>0.79 (0.60-1.06)	0.3935	0.9151	0.82 (0.28-2.45)<br>0.81 (0.62-1.06)	0.4532	0.8154	0.93 (0.32-2.69)<br>0.81 (0.58-1.13)	0.5100	0.8065
rs1260326 (*GCKR*)	TT vs. CC<br>TC vs. CC	1.36 (0.77-2.41)<br>1.47 (0.99-2.19)	0.1887	0.9151	1.54 (0.88-2.68)<br>1.52 (1.01-2.30)	0.1270	0.8154	1.63 (0.96-2.77)<br>1.50 (1.01-2.24)	0.1399	0.7270
rs1143623 (*IL1B*)	CC vs. GG<br>CG vs. GG	1.21 (0.58-2.51)<br>1.29 (0.92-1.81)	0.3459	0.9151	1.37 (0.66-2.82)<br>1.27 (0.89-1.80)	0.3326	0.8154	1.45 (0.70-3.00)<br>1.39 (0.97-1.99)	0.1731	0.7270
rs1800871 (*IL10*)	TT vs. CC<br>TC vs. CC	0.71 (0.31-1.66)<br>1.06 (0.75-1.51)	0.6227	0.9151	0.72 (0.29-1.77)<br>1.10 (0.77-1.56)	0.6000	0.8154	0.67 (0.31-1.46)<br>1.12 (0.78-1.63)	0.4406	0.8065
rs1800872 (*IL10*)	AA vs. CC<br>AC vs. CC	0.63 (0.26-1.54)<br>1.05 (0.73-1.49)	0.5113	0.9151	0.65 (0.26-1.66)<br>1.08 (0.76-1.53)	0.5137	0.8154	0.65 (0.29-1.44)<br>1.13 (0.79-1.62)	0.3938	0.8065
rs1800896 (*IL10*)	GG vs. AA<br>GA vs. AA	0.75 (0.50-1.15)<br>1.24 (0.92-1.69)	**0.0275**	0.7562	0.71 (0.47-1.07)<br>1.17 (0.85-1.62)	**0.0350**	0.8154	0.70 (0.45-1.10)<br>1.22 (0.89-1.67)	**0.0260**	0.7020
rs2243248 (*IL4*)	GG vs. TT<br>GT vs. TT	0.72 (0.10-5.25)<br>0.98 (0.59-1.63)	0.9419	0.9910	0.88 (0.14-5.65)<br>1.21 (0.76-1.94)	0.5835	0.8154	0.30 (0.03-2.80)<br>1.02 (0.51-2.01)	0.7613	0.8661
rs2243250 (*IL4*)	TT vs. CC<br>TC vs. CC	0.57 (0.17-1.96)<br>0.87 (0.58-1.29)	0.5226	0.9151	0.52 (0.14-1.99)<br>0.83 (0.57-1.22)	0.3977	0.8154	0.46 (0.12-1.87)<br>0.85 (0.57-1.25)	0.3746	0.8065
rs2243270 (*IL4*)	GG vs. AA<br>GA vs. AA	0.47 (0.12-1.77)<br>0.87 (0.58-1.29)	0.4118	0.9151	0.44 (0.11-1.78)<br>0.83 (0.56-1.22)	0.3149	0.8154	0.49 (0.12-2.02)<br>0.83 (0.55-1.24)	0.3793	0.8065
rs1801275 (*IL4R*)	GG vs. AA<br>GA vs. AA	1.08 (0.54-2.14)<br>0.92 (0.70-1.20)	0.7753	0.9706	1.33 (0.63-2.79)<br>0.94 (0.72-1.24)	0.5595	0.8154	1.32 (0.60-2.91)<br>1.00 (0.71-1.41)	0.7454	0.8661

Variant	Genotype	Crude Model			Age-Sex Adjusted Model			Fully Adjusted[b] Model		
		OR (95% CI)	P-value	FDR-adjusted P-value	OR (95% CI)	P-value	FDR-adjusted P-value	OR (95% CI)	P-value	FDR-adjusted P-value
rs1805015 (*IL4R*)	CC vs. TT<br>CT vs. TT	1.43 (0.64-3.22)<br>0.91 (0.62-1.34)	0.5597	0.9151	1.72 (0.74-3.97)<br>0.93 (0.62-1.39)	0.4076	0.8154	1.77 (0.69-4.54)<br>0.96 (0.65-1.43)	0.4441	0.8065
rs5918 (*ITGB3*)	CC vs. TT<br>CT vs. TT	1.49 (0.61-3.65)<br>0.94 (0.62-1.42)	0.5919	0.9151	1.71 (0.77-3.80)<br>0.93 (0.60-1.45)	0.4331	0.8154	1.87 (0.83-4.24)<br>0.85 (0.58-1.24)	0.1973	0.7270
rs11003125 (*MBL2*)	GG vs. CC<br>GC vs. CC	1.57 (0.92-2.69)<br>1.30 (0.92-1.85)	0.1740	0.9151	1.49 (0.86-2.58)<br>1.25 (0.89-1.74)	0.2577	0.8154	1.60 (0.90-2.83)<br>1.19 (0.83-1.71)	0.2123	0.7270
rs1800450 (*MBL2*)	AA vs. GG<br>AG vs. GG	1.51 (0.70-3.24)<br>1.07 (0.73-1.59)	0.5551	0.9151	1.45 (0.61-3.43)<br>1.02 (0.68-1.51)	0.6683	0.8420	1.58 (0.67-3.75)<br>0.97 (0.63-1.49)	0.5457	0.8170
rs1800451 (*MBL2*)	AA vs. GG<br>AG vs. GG	20.50 (1.43-294.32)<br>1.18 (0.60-2.32)	0.0725	0.7997						
rs5030737 (*MBL2*)	TT vs. CC<br>TC vs. CC	0.79 (0.17-3.75)<br>1.35 (0.71-2.56)	0.3934	0.9151	0.56 (0.10-3.16)<br>1.43 (0.75-2.73)	0.2983	0.8154	0.57 (0.10-3.28)<br>1.54 (0.82-2.87)	0.2032	0.7270
rs7096206 (*MBL2*)	CC vs. GG<br>CG vs. GG	1.25 (0.65-2.39)<br>0.73 (0.46-1.16)	0.2276	0.9151	1.24 (0.65-2.37)<br>0.73 (0.46-1.15)	0.2080	0.8154	1.50 (0.73-3.07)<br>0.72 (0.45-1.15)	0.1358	0.7270
rs1800482 (*NOS2A*)	CC vs. GG<br>CG vs. GG	2.20 (0.27-18.09)	0.4480	0.9151	1.43 (0.20-10.31)	0.7146	0.8420	1.52 (0.26-8.93)	0.6284	0.8661
rs9282799 (*NOS2A*)	TT vs. CC<br>TC vs. CC	1.24 (0.11-14.20)	0.8573	0.9774	0.60 (0.05-7.84)	0.6862	0.8420	0.60 (0.05-7.25)	0.6744	0.8661
rs1799983 (*NOS3*)	TT vs. GG<br>TG vs. GG	1.36 (0.58-3.20)<br>1.07 (0.78-1.45)	0.6050	0.9151	1.52 (0.68-3.40)<br>1.10 (0.77-1.55)	0.4548	0.8154	1.45 (0.68-3.10)<br>1.05 (0.74-1.50)	0.5099	0.8065
rs2070744 (*NOS3*)	CC vs. TT<br>CT vs. TT	1.07 (0.69-1.65)<br>1.17 (0.84-1.65)	0.5830	0.9151	1.12 (0.70-1.79)<br>1.20 (0.85-1.70)	0.5217	0.8154	1.12 (0.66-1.90)<br>1.15 (0.82-1.62)	0.7123	0.8661
rs662 (*PON1*)	GG vs. AA<br>GA vs. AA	1.29 (0.71-2.35)<br>1.02 (0.70-1.51)	0.6324	0.9151	1.44 (0.82-2.53)<br>1.10 (0.75-1.61)	0.4084	0.8154	1.73 (0.96-3.10)<br>1.24 (0.84-1.81)	0.1373	0.7270
rs854560 (*PON1*)	AA vs. TT<br>AT vs. TT	0.92 (0.47-1.78)<br>1.50 (1.13-2.00)	**0.0428**	0.7847	0.93 (0.51-1.71)<br>1.45 (1.08-1.96)	0.0567	0.8154	0.78 (0.44-1.38)<br>1.44 (0.99-2.07)	0.0507	0.7270
rs1801282 (*PPARG*)	GG vs. CC<br>GC vs. CC	1.17 (0.37-3.75)<br>1.32 (0.88-1.97)	0.3837	0.9151	1.53 (0.56-4.17)<br>1.32 (0.90-1.95)	0.2557	0.8154	1.27 (0.27-5.98)<br>1.48 (0.98-2.25)	0.2154	0.7270
rs1799762 (*SERPINE1*)	4G4G vs. 5G5G<br>4G5G vs. 5G5G	0.95 (0.60-1.51)<br>1.24 (0.83-1.84)	0.4086	0.9151	0.98 (0.60-1.60)<br>1.23 (0.81-1.87)	0.5071	0.8154	1.09 (0.64-1.85)<br>1.38 (0.90-2.10)	0.4067	0.8065
rs1800468 (*TGFB1/B9D2*)	AA vs. GG<br>AG vs. GG	4.28 (0.95-19.17)<br>1.05 (0.63-1.76)	0.1582	0.9151	4.70 (0.92-23.92)<br>1.13 (0.66-1.91)	0.1565	0.8154	4.36 (0.80-23.66)<br>1.21 (0.72-2.05)	0.1638	0.7270
rs1800469 (*TGFB1/B9D2*)	TT vs. CC<br>TC vs. CC	1.37 (0.77-2.45)<br>1.27 (0.80-2.00)	0.4256	0.9151	1.45 (0.82-2.57)<br>1.24 (0.79-1.96)	0.4110	0.8154	1.48 (0.82-2.69)<br>1.24 (0.79-1.95)	0.3925	0.8065
rs1800470 (*TGFB1*)	CC vs. TT<br>CT vs. TT	1.38 (0.81-2.34)<br>1.13 (0.72-1.77)	0.4598	0.9151	1.35 (0.81-2.25)<br>1.11 (0.71-1.75)	0.5027	0.8154	1.40 (0.82-2.36)<br>1.12 (0.70-1.79)	0.4609	0.8065
rs4986790 (*TLR4*)	GG vs. AA<br>GA vs. AA	0.74 (0.09-6.42)<br>1.32 (0.80-2.18)	0.4528	0.9151	0.83 (0.08-8.09)<br>1.30 (0.78-2.18)	0.5284	0.8154	0.79 (0.08-8.16)<br>1.23 (0.72-2.09)	0.6923	0.8661
rs1800629 (*TNF*)	AA vs. GG<br>AG vs. GG	0.68 (0.32-1.46)<br>0.91 (0.55-1.51)	0.6689	0.9151	0.79 (0.36-1.70)<br>0.89 (0.53-1.47)	0.7149	0.8420	0.72 (0.32-1.63)<br>0.96 (0.53-1.72)	0.7859	0.8661
rs1800750 (*TNF*)	AA vs. GG<br>AG vs. GG									
rs361525 (*TNF*)	AA vs. GG<br>AG vs. GG	5.59 (1.55-20.10)<br>1.36 (0.88-2.10)	**0.0143**	0.7562	7.53 (1.57-36.14)<br>1.47 (0.96-2.24)	**0.0127**	0.6731	13.09 (2.87-59.68)<br>1.30 (0.77-2.17)	**0.0027**	0.1458
rs2239185 (*VDR*)	CC vs. TT<br>CT vs. TT	0.64 (0.44-0.93)<br>0.88 (0.59-1.29)	0.1331	0.9151	0.66 (0.43-1.00)<br>0.88 (0.59-1.32)	0.2111	0.8154	0.70 (0.47-1.04)<br>0.92 (0.60-1.41)	0.3082	0.8065
rs731236 (*VDR*)	CC vs. TT<br>CT vs. TT	1.69 (1.04-2.75)<br>1.37 (0.95-1.97)	0.0727	0.7997	1.63 (1.00-2.65)<br>1.35 (0.92-1.98)	0.1002	0.8154	1.54 (0.92-2.59)<br>1.38 (0.92-2.08)	0.1746	0.7270
rs890945 (Chr 5q33.3)	AA vs. TT<br>AT vs. TT	1.60 (0.69-3.71)<br>1.10 (0.73-1.66)	0.4936	0.9151	1.88 (0.91-3.88)<br>1.14 (0.74-1.76)	0.2595	0.8154	1.30 (0.67-2.53)<br>1.11 (0.65-1.87)	0.7295	0.8661
Non-Hispanic blacks										
rs1042713 (*ADRB2*)	AA vs. GG<br>AG vs. GG	0.92 (0.51-1.67)<br>1.00 (0.69-1.45)	0.8962	0.9876	0.99 (0.51-1.93)<br>1.01 (0.68-1.50)	0.9926	0.9926	1.00 (0.45-2.21)<br>1.20 (0.69-2.08)	0.7379	0.8524
rs1042714 (*ADRB2*)	GG vs. CC<br>GC vs. CC	0.26 (0.07-1.01)<br>1.18 (0.77-1.80)	0.1739	0.9065	0.29 (0.07-1.11)<br>1.05 (0.66-1.67)	0.3259	0.8596	0.33 (0.09-1.26)<br>1.05 (0.60-1.83)	0.4376	0.7750
rs429358 (*APOE*)	CC vs. TT<br>CT vs. TT	1.54 (0.65-3.65)<br>1.14 (0.74-1.75)	0.5212	0.9531	1.56 (0.67-3.61)<br>1.33 (0.86-2.06)	0.2941	0.8596	1.35 (0.59-3.12)<br>1.25 (0.82-1.89)	0.4702	0.7750
rs7412 (*APOE*)	TT vs. CC<br>TC vs. CC	0.71 (0.08-6.19)<br>1.27 (0.76-2.10)	0.4971	0.9531	0.74 (0.10-5.77)<br>1.17 (0.73-1.87)	0.6877	0.8687	0.84 (0.11-6.66)<br>1.27 (0.80-2.01)	0.4903	0.7750
rs769214 (*CAT*)	GG vs. AA<br>GA vs. AA	0.72 (0.47-1.09)<br>0.85 (0.60-1.21)	0.3767	0.9531	0.70 (0.45-1.09)<br>0.85 (0.60-1.20)	0.3341	0.8596	0.78 (0.48-1.26)<br>0.88 (0.56-1.36)	0.6160	0.8245
rs2280788 (*CCL5*)	GG vs. CC<br>GC vs. CC									
rs1799864 (*CCR2*)	AA vs. GG<br>AG vs. GG	1.23 (0.38-4.03)<br>0.81 (0.50-1.32)	0.5565	0.9531	1.33 (0.52-3.38)<br>0.82 (0.54-1.26)	0.4676	0.8596	0.97 (0.33-2.83)<br>0.80 (0.52-1.25)	0.4838	0.7750
rs1205 (*CRP*)	AA vs. GG<br>AG vs. GG	0.42 (0.13-1.33)<br>0.83 (0.65-1.05)	0.1394	0.9065	0.35 (0.11-1.12)<br>0.89 (0.68-1.15)	0.0954	0.8596	0.39 (0.11-1.36)<br>0.84 (0.65-1.09)	0.1486	0.7750
rs1417938 (*CRP*)	TT vs. AA<br>TA vs. AA									
rs1800947 (*CRP*)	CC vs. GG<br>CG vs. GG	0.94 (0.23-3.79)	0.9313	0.9876	0.71 (0.18-2.75)	0.6075	0.8596	0.82 (0.22-2.97)	0.7480	0.8524
rs2808630 (*CRP*)	GG vs. AA<br>GA vs. AA	1.12 (0.44-2.89)<br>1.27 (0.96-1.69)	0.2766	0.9065	0.88 (0.36-2.12)<br>1.23 (0.94-1.61)	0.2983	0.8596	0.66 (0.31-1.40)<br>1.24 (0.94-1.64)	0.1511	0.7750

Variant	Genotype	Crude Model			Age-Sex Adjusted Model			Fully Adjusted[b] Model		
		OR (95% CI)	P-value	FDR-adjusted P-value	OR (95% CI)	P-value	FDR-adjusted P-value	OR (95% CI)	P-value	FDR-adjusted P-value
rs3091244 (*CRP*)	AA vs. CC<br>AC vs. CC<br>AT vs. CC<br>CT vs. CC<br>TT vs. CC	0.91 (0.45-1.83)<br>1.15 (0.57-2.31)<br>1.41 (0.60-3.34)<br>0.93 (0.45-1.89)<br>0.65 (0.27-1.55)	0.5729	0.9531	0.94 (0.45-1.97)<br>1.27 (0.62-2.59)<br>1.61 (0.72-3.64)<br>1.10 (0.49-2.46)<br>0.68 (0.27-1.66)	0.5061	0.8596	0.71 (0.36-1.41)<br>1.42 (0.71-2.86)<br>1.74 (0.72-4.19)<br>1.11 (0.47-2.65)<br>0.92 (0.36-2.35)	0.4049	0.7750
rs3093058 (*CRP*)	TT vs. AA<br>TA vs. AA	0.45 (0.15-1.39)<br>0.76 (0.52-1.11)	0.1529	0.9065	0.47 (0.15-1.50)<br>0.80 (0.55-1.15)	0.2242	0.8596	0.51 (0.15-1.71)<br>0.72 (0.49-1.07)	0.1589	0.7750
rs3093066 (*CRP*)	AA vs. CC<br>AC vs. CC	1.06 (0.48-2.32)<br>1.50 (0.85-2.65)	0.2233	0.9065	1.19 (0.52-2.73)<br>1.52 (0.90-2.56)	0.1947	0.8596	0.63 (0.23-1.76)<br>1.54 (0.88-2.71)	0.1406	0.7750
rs11265260 (*CRP*)	GG vs. AA<br>GA vs. AA	2.36 (0.23-23.69)<br>1.04 (0.65-1.64)	0.6808	0.9531	3.02 (0.54-16.92)<br>1.04 (0.59-1.81)	0.5201	0.8596	3.29 (0.62-17.49)<br>1.09 (0.57-2.09)	0.4808	0.7750
rs12093699 (*CRP*)	AA vs. GG<br>AG vs. GG	1.07 (0.62-1.86)<br>1.01 (0.62-1.66)	0.9674	0.9876	1.06 (0.61-1.82)<br>1.00 (0.58-1.72)	0.9751	0.9926	0.90 (0.49-1.68)<br>1.00 (0.53-1.88)	0.9337	0.9734
rs12744244 (*CRP*)	AA vs. CC<br>AC vs. CC									
rs2027471 (*CRP*)	AA vs. TT<br>AT vs. TT	0.74 (0.29-1.89)<br>0.72 (0.50-1.03)	0.2688	0.9065	0.63 (0.21-1.85)<br>0.78 (0.54-1.13)	0.3770	0.8596	0.66 (0.20-2.14)<br>0.72 (0.50-1.03)	0.3307	0.7750
rs2592887 (*CRP*)	AA vs. GG<br>AG vs. GG	1.02 (0.59-1.77)<br>0.88 (0.60-1.30)	0.6682	0.9531	1.00 (0.58-1.74)<br>0.93 (0.70-1.25)	0.8658	0.9445	0.88 (0.48-1.60)<br>0.81 (0.57-1.14)	0.5935	0.8245
rs2794520 (*CRP*)	AA vs. GG<br>AG vs. GG	0.78 (0.32-1.89)<br>0.79 (0.56-1.10)	0.4197	0.9531	0.67 (0.24-1.89)<br>0.87 (0.61-1.23)	0.5486	0.8596	0.74 (0.24-2.25)<br>0.81 (0.58-1.14)	0.5356	0.8201
rs3093075 (*CRP*)	AA vs. CC<br>AC vs. CC	0.78 (0.40-1.55)<br>1.43 (0.86-2.38)	0.1747	0.9065	0.76 (0.37-1.55)<br>1.44 (0.87-2.37)	0.1704	0.8596	0.52 (0.26-1.01)<br>1.57 (0.92-2.68)	**0.0463**	0.7750
rs1799963 (*F2*)	AG vs. GG									
rs6025 (*F5*)	AA vs. GG<br>AG vs. GG	 1.05 (0.20-5.38)	0.9521	0.9876	 1.51 (0.27-8.39)	0.6234	0.8596	 1.79 (0.33-9.71)	0.4831	0.7750
rs1801274 (*FCGR2A*)	AA vs. GG<br>AG vs. GG	1.79 (0.79-4.06)<br>1.44 (0.83-2.50)	0.2476	0.9065	2.05 (0.87-4.85)<br>1.58 (0.85-2.91)	0.1511	0.8596	1.95 (0.80-4.74)<br>1.57 (0.80-3.09)	0.2270	0.7750
rs1800790 (*FGB*)	AA vs. GG<br>AG vs. GG	1.41 (0.35-5.79)<br>0.72 (0.36-1.43)	0.4819	0.9531	1.28 (0.43-3.81)<br>0.76 (0.36-1.62)	0.5612	0.8596	2.48 (1.15-5.33)<br>0.91 (0.42-1.98)	0.4301	0.7750
rs1260326 (*GCKR*)	TT vs. CC<br>TC vs. CC	0.41 (0.10-1.67)<br>0.88 (0.57-1.35)	0.3815	0.9531	0.34 (0.08-1.55)<br>0.82 (0.52-1.31)	0.2738	0.8596	0.37 (0.08-1.78)<br>0.77 (0.47-1.26)	0.2708	0.7750
rs1143623 (*IL1B*)	CC vs. GG<br>CG vs. GG	1.90 (0.42-8.64)<br>0.75 (0.50-1.12)	0.2775	0.9065	2.86 (0.57-14.29)<br>0.75 (0.48-1.15)	0.1619	0.8596	5.64 (1.47-21.65)<br>0.83 (0.55-1.24)	**0.0209**	0.7750
rs1800871 (*IL10*)	TT vs. CC<br>TC vs. CC	0.86 (0.45-1.62)<br>0.99 (0.67-1.46)	0.8556	0.9876	0.83 (0.41-1.69)<br>0.99 (0.65-1.49)	0.8243	0.9201	0.96 (0.47-1.97)<br>0.96 (0.60-1.53)	0.9812	0.9812
rs1800872 (*IL10*)	AA vs. CC<br>AC vs. CC	0.82 (0.42-1.62)<br>0.98 (0.66-1.46)	0.8102	0.9859	0.79 (0.37-1.70)<br>0.98 (0.64-1.50)	0.7763	0.8872	0.91 (0.43-1.94)<br>0.95 (0.59-1.53)	0.9560	0.9759
rs1800896 (*IL10*)	GG vs. AA<br>GA vs. AA	0.82 (0.49-1.36)<br>0.92 (0.57-1.49)	0.7548	0.9859	0.78 (0.45-1.36)<br>0.87 (0.53-1.44)	0.6462	0.8616	0.77 (0.39-1.50)<br>0.85 (0.51-1.43)	0.6587	0.8245
rs2243248 (*IL4*)	GG vs. TT<br>GT vs. TT	1.16 (0.37-3.59)<br>1.13 (0.74-1.75)	0.8122	0.9859	1.73 (0.51-5.85)<br>1.11 (0.73-1.70)	0.5782	0.8596	2.00 (0.60-6.62)<br>0.96 (0.59-1.56)	0.4819	0.7750
rs2243250 (*IL4*)	TT vs. CC<br>TC vs. CC	1.33 (0.56-3.17)<br>1.02 (0.48-2.20)	0.4922	0.9531	1.39 (0.54-3.59)<br>1.01 (0.44-2.34)	0.4483	0.8596	1.79 (0.63-5.06)<br>1.18 (0.43-3.25)	0.2702	0.7750
rs2243270 (*IL4*)	GG vs. AA<br>GA vs. AA	0.84 (0.41-1.72)<br>0.66 (0.41-1.07)	0.4380	0.9531	0.90 (0.41-1.98)<br>0.65 (0.40-1.07)	0.3792	0.8596	0.98 (0.42-2.31)<br>0.71 (0.40-1.24)	0.4725	0.7750
rs1801275 (*IL4R*)	GG vs. AA<br>GA vs. AA	1.45 (0.83-2.53)<br>1.31 (0.65-2.61)	0.4706	0.9531	1.37 (0.72-2.60)<br>1.39 (0.60-3.20)	0.6268	0.8596	1.21 (0.60-2.45)<br>1.36 (0.60-3.11)	0.6892	0.8245
rs1805015 (*IL4R*)	CC vs. TT<br>CT vs. TT	1.17 (0.82-1.68)<br>0.96 (0.67-1.36)	0.6141	0.9531	1.18 (0.77-1.81)<br>1.00 (0.71-1.40)	0.7157	0.8809	0.91 (0.57-1.46)<br>0.86 (0.59-1.26)	0.6899	0.8245
rs5918 (*ITGB3*)	CC vs. TT<br>CT vs. TT	0.65 (0.09-4.68)<br>1.34 (0.98-1.83)	0.2365	0.9065	0.61 (0.08-4.37)<br>1.22 (0.86-1.72)	0.4832	0.8596	1.25 (0.20-8.03)<br>1.32 (0.87-2.00)	0.3932	0.7750
rs11003125 (*MBL2*)	GG vs. CC<br>GC vs. CC	1.34 (0.33-5.47)<br>0.76 (0.46-1.24)	0.4909	0.9531	1.07 (0.32-3.59)<br>0.81 (0.49-1.34)	0.6801	0.8687	1.13 (0.35-3.60)<br>0.79 (0.44-1.41)	0.6556	0.8245
rs1800450 (*MBL2*)	AA vs. GG<br>AG vs. GG	2.45 (0.39-15.35)<br>1.17 (0.47-2.91)	0.6611	0.9531	3.18 (0.83-12.27)<br>1.15 (0.48-2.75)	0.5027	0.8596	3.74 (0.95-14.76)<br>1.38 (0.53-3.56)	0.3488	0.7750
rs1800451 (*MBL2*)	AA vs. GG<br>AG vs. GG	1.10 (0.58-2.10)<br>1.55 (0.98-2.46)	0.1121	0.9065	1.25 (0.62-2.50)<br>1.53 (0.98-2.39)	0.1219	0.8596	1.34 (0.64-2.80)<br>1.42 (0.86-2.35)	0.2947	0.7750
rs5030737 (*MBL2*)	TT vs. CC<br>TC vs. CC	 0.68 (0.13-3.50)	0.6281	0.9531	 0.90 (0.15-5.44)	0.9045	0.9648	 1.22 (0.21-7.25)	0.8156	0.9080
rs7096206 (*MBL2*)	CC vs. GG<br>CG vs. GG	0.46 (0.10-2.20)<br>0.73 (0.44-1.20)	0.2742	0.9065	0.37 (0.07-1.95)<br>0.76 (0.48-1.20)	0.2577	0.8596	0.53 (0.10-2.92)<br>0.66 (0.39-1.12)	0.2419	0.7750
rs1800482 (*NOS2A*)	CC vs. GG<br>CG vs. GG	5.08 (0.80-32.24)<br>0.91 (0.60-1.38)	0.1118	0.9065	5.82 (0.58-58.24)<br>0.84 (0.52-1.34)	0.1579	0.8596	5.89 (0.64-54.20)<br>1.01 (0.65-1.58)	0.1555	0.7750
rs9282799 (*NOS2A*)	TT vs. CC<br>TC vs. CC									
rs1799983 (*NOS3*)	TT vs. GG<br>TG vs. GG									
rs2070744 (*NOS3*)	CC vs. TT<br>CT vs. TT	1.06 (0.22-5.13)<br>1.02 (0.67-1.54)	0.9937	0.9937	0.91 (0.18-4.46)<br>1.02 (0.70-1.48)	0.9837	0.9926	0.73 (0.07-7.88)<br>1.01 (0.70-1.46)	0.9243	0.9734
rs662 (*PON1*)	GG vs. AA<br>GA vs. AA	0.98 (0.65-1.49)<br>1.18 (0.69-2.01)	0.5432	0.9531	1.06 (0.67-1.68)<br>1.17 (0.69-1.96)	0.7697	0.8872	0.91 (0.53-1.56)<br>1.15 (0.70-1.90)	0.4825	0.7750
rs854560 (*PON1*)	AA vs. TT<br>AT vs. TT	1.23 (0.54-2.76)<br>0.93 (0.53-1.63)	0.8249	0.9859	1.25 (0.63-2.49)<br>0.83 (0.47-1.47)	0.5370	0.8596	0.97 (0.41-2.33)<br>0.81 (0.41-1.58)	0.6278	0.8245
rs1801282 (*PPARG*)	GG vs. CC<br>GC vs. CC									

Variant	Genotype	Crude Model			Age-Sex Adjusted Model			Fully Adjusted[b] Model		
		**OR (95% CI)**	**P-value**	**FDR-adjusted P-value**	**OR (95% CI)**	**P-value**	**FDR-adjusted P-value**	**OR (95% CI)**	**P-value**	**FDR-adjusted P-value**
rs1799762 (*SERPINE1*)	4G4G vs. 5G5G<br>4G5G vs. 5G5G	1.01 (0.49-2.06)<br>0.87 (0.58-1.32)	0.7465	0.9859	1.04 (0.46-2.34)<br>0.81 (0.53-1.25)	0.5880	0.8596	1.17 (0.55-2.51)<br>0.72 (0.47-1.11)	0.2224	0.7750
rs1800468 (*TGFB1/B9D2*)	AA vs. GG<br>AG vs. GG									
rs1800469 (*TGFB1/B9D2*)	TT vs. CC<br>TC vs. CC	0.50 (0.19-1.33)<br>1.01 (0.68-1.49)	0.3510	0.9531	0.42 (0.14-1.24)<br>0.97 (0.62-1.52)	0.2705	0.8596	0.52 (0.18-1.53)<br>1.00 (0.61-1.62)	0.4538	0.7750
rs1800470 (*TGFB1*)	CC vs. TT<br>CT vs. TT	1.06 (0.58-1.92)<br>0.86 (0.54-1.38)	0.6709	0.9531	1.02 (0.51-2.02)<br>0.86 (0.52-1.40)	0.7356	0.8827	1.16 (0.55-2.45)<br>1.00 (0.59-1.71)	0.8339	0.9080
rs4986790 (*TLR4*)	GG vs. AA<br>GA vs. AA	1.32 (0.14-12.64)<br>0.92 (0.50-1.70)	0.9160	0.9876	3.06 (0.31-30.18)<br>0.88 (0.48-1.61)	0.5287	0.8596	2.57 (0.25-26.33)<br>0.81 (0.36-1.83)	0.6008	0.8245
rs1800629 (*TNF*)	AA vs. GG<br>AG vs. GG									
rs1800750 (*TNF*)	AA vs. GG<br>AG vs. GG									
rs361525 (*TNF*)	AA vs. GG<br>AG vs. GG									
rs2239185 (*VDR*)	CC vs. TT<br>CT vs. TT	1.16 (0.73-1.85)<br>1.12 (0.74-1.69)	0.7879	0.9859	1.11 (0.67-1.85)<br>1.23 (0.80-1.88)	0.5987	0.8596	1.07 (0.65-1.77)<br>1.50 (0.96-2.33)	0.1262	0.7750
rs731236 (*VDR*)	CC vs. TT<br>CT vs. TT	1.00 (0.67-1.48)<br>1.35 (0.97-1.88)	0.0916	0.9065	0.95 (0.62-1.46)<br>1.37 (0.93-2.01)	0.1212	0.8596	1.05 (0.62-1.77)<br>1.39 (0.93-2.07)	0.1543	0.7750
rs890945 (Chr 5q33.3)	AA vs. TT<br>AT vs. TT	0.53 (0.27-1.05)<br>0.86 (0.56-1.32)	0.2632	0.9065	0.54 (0.29-0.99)<br>0.84 (0.54-1.28)	0.2354	0.8596	0.45 (0.22-0.91)<br>0.79 (0.51-1.23)	0.1294	0.7750
Mexican Americans										
rs1042713 (*ADRB2*)	AA vs. GG<br>AG vs. GG	1.05 (0.61-1.81)<br>0.86 (0.56-1.31)	0.5411	0.8503	1.13 (0.70-1.83)<br>0.89 (0.57-1.38)	0.5093	0.9269	1.04 (0.65-1.66)<br>0.86 (0.50-1.46)	0.5837	0.9226
rs1042714 (*ADRB2*)	GG vs. CC<br>GC vs. CC	0.84 (0.34-2.08)<br>1.15 (0.73-1.81)	0.6941	0.8503	0.81 (0.37-1.79)<br>1.14 (0.75-1.72)	0.6421	0.9371	0.89 (0.36-2.23)<br>1.26 (0.86-1.86)	0.3939	0.7501
rs429358 (*APOE*)	CC vs. TT<br>CT vs. TT	1.00 (0.33-3.02)<br>1.24 (0.87-1.78)	0.4521	0.8503	1.13 (0.40-3.19)<br>1.23 (0.87-1.73)	0.4575	0.9269	1.07 (0.35-3.25)<br>1.26 (0.88-1.80)	0.4133	0.7501
rs7412 (*APOE*)	TT vs. CC<br>TC vs. CC									
rs769214 (*CAT*)	GG vs. AA<br>GA vs. AA	1.34 (0.68-2.65)<br>1.17 (0.68-2.00)	0.5558	0.8503	1.37 (0.68-2.76)<br>1.27 (0.70-2.30)	0.5462	0.9269	1.26 (0.57-2.81)<br>1.12 (0.62-2.02)	0.7026	0.9353
rs2280788 (*CCL5*)	GG vs. CC<br>GC vs. CC	0.89 (0.16-5.07)	0.8891	0.9681	0.84 (0.14-5.13)	0.8393	0.9633	1.08 (0.17-7.02)	0.9353	0.9353
rs1799864 (*CCR2*)	AA vs. GG<br>AG vs. GG	1.51 (0.49-4.66)<br>0.97 (0.59-1.58)	0.6933	0.8503	1.76 (0.53-5.79)<br>1.03 (0.64-1.66)	0.5552	0.9269	1.95 (0.57-6.72)<br>1.00 (0.66-1.52)	0.4089	0.7501
rs1205 (*CRP*)	AA vs. GG<br>AG vs. GG	0.99 (0.42-2.32)<br>1.20 (0.84-1.71)	0.6021	0.8503	0.94 (0.37-2.43)<br>1.16 (0.82-1.64)	0.7037	0.9371	0.89 (0.34-2.35)<br>1.12 (0.76-1.66)	0.7625	0.9353
rs1417938 (*CRP*)	TT vs. AA<br>TA vs. AA	1.24 (0.47-3.25)<br>1.20 (0.87-1.65)	0.6654	0.8503	1.18 (0.45-3.13)<br>1.18 (0.84-1.66)	0.7293	0.9371	1.14 (0.41-3.16)<br>1.20 (0.88-1.65)	0.7056	0.9353
rs1800947 (*CRP*)	CC vs. GG<br>CG vs. GG	0.37 (0.08-1.77)	0.2022	0.6440	0.23 (0.04-1.28)	0.0905	0.6694	0.08 (0.03-0.20)	**<0.0001**	**<0.0001**
rs2808630 (*CRP*)	GG vs. AA<br>GA vs. AA	0.94 (0.39-2.28)<br>0.73 (0.40-1.33)	0.4815	0.8503	0.91 (0.35-2.39)<br>0.79 (0.44-1.41)	0.6512	0.9371	0.98 (0.42-2.29)<br>0.90 (0.50-1.62)	0.8901	0.9353
rs3091244 (*CRP*)	AA vs. CC<br>AC vs. CC<br>AT vs. CC<br>CT vs. CC<br>TT vs. CC									
rs3093058 (*CRP*)	TT vs. AA<br>TA vs. AA									
rs3093066 (*CRP*)	AA vs. CC<br>AC vs. CC	0.37 (0.08-1.63)	0.1794	0.6440	0.35 (0.08-1.53)	0.1534	0.6694	0.23 (0.03-1.57)	0.1263	0.5157
rs11265260 (*CRP*)	GG vs. AA<br>GA vs. AA									
rs12093699 (*CRP*)	AA vs. GG<br>AG vs. GG	1.11 (0.56-2.21)<br>1.03 (0.72-1.47)	0.9320	0.9722	1.12 (0.59-2.13)<br>0.98 (0.65-1.47)	0.8985	0.9802	1.04 (0.51-2.11)<br>0.94 (0.62-1.41)	0.9198	0.9353
rs12744244 (*CRP*)	AA vs. CC<br>AC vs. CC	0.92 (0.14-5.92)<br>0.95 (0.56-1.59)	0.9587	0.9722	0.97 (0.17-5.66)<br>0.92 (0.54-1.57)	0.9313	0.9878	1.07 (0.17-6.77)<br>0.92 (0.59-1.43)	0.9310	0.9353
rs2027471 (*CRP*)	AA vs. TT<br>AT vs. TT	1.02 (0.50-2.05)<br>1.07 (0.73-1.56)	0.9412	0.9722	0.99 (0.46-2.14)<br>1.00 (0.68-1.47)	0.9988	0.9988	0.90 (0.43-1.91)<br>0.93 (0.61-1.44)	0.9266	0.9353
rs2592887 (*CRP*)	AA vs. GG<br>AG vs. GG	1.08 (0.56-2.09)<br>1.18 (0.86-1.61)	0.6828	0.8503	1.04 (0.51-2.11)<br>1.09 (0.80-1.49)	0.8749	0.9766	1.00 (0.49-2.03)<br>1.06 (0.77-1.44)	0.9344	0.9353
rs2794520 (*CRP*)	AA vs. GG<br>AG vs. GG	1.02 (0.49-2.13)<br>1.10 (0.79-1.53)	0.8642	0.9624	0.98 (0.42-2.29)<br>1.05 (0.74-1.47)	0.9466	0.9878	0.92 (0.41-2.08)<br>1.03 (0.71-1.50)	0.9332	0.9353
rs3093075 (*CRP*)	AA vs. CC<br>AC vs. CC									
rs1799963 (*F2*)	AG vs. GG	2.43 (0.67-8.84)	0.1676	0.6440	2.39 (0.56-10.17)	0.2245	0.7161	2.78 (0.69-11.27)	0.1439	0.5163
rs6025 (*F5*)	AA vs. GG<br>AG vs. GG									
rs1801274 (*FCGR2A*)	AA vs. GG<br>AG vs. GG	1.67 (0.91-3.07)<br>1.21 (0.82-1.78)	0.1202	0.6440	1.59 (0.86-2.95)<br>1.09 (0.75-1.58)	0.1401	0.6694	1.61 (0.82-3.17)<br>1.15 (0.78-1.70)	0.1964	0.6015

Variant	Genotype	Crude Model			Age-Sex Adjusted Model			Fully Adjusted[b] Model		
		OR (95% CI)	P-value	FDR-adjusted P-value	OR (95% CI)	P-value	FDR-adjusted P-value	OR (95% CI)	P-value	FDR-adjusted P-value
rs1800790 (*FGB*)	AA vs. GG<br>AG vs. GG	1.00 (0.18-5.57)<br>0.72 (0.40-1.27)	0.5033	0.8503	1.44 (0.27-7.76)<br>0.75 (0.43-1.33)	0.5127	0.9269	1.52 (0.25-9.10)<br>0.81 (0.40-1.67)	0.6986	0.9353
rs1260326 (*GCKR*)	TT vs. CC<br>TC vs. CC	1.56 (0.87-2.81)<br>0.92 (0.60-1.41)	0.1972	0.6440	1.51 (0.83-2.75)<br>0.95 (0.61-1.49)	0.3063	0.8168	1.55 (0.85-2.84)<br>1.02 (0.65-1.60)	0.3550	0.7501
rs1143623 (*IL1B*)	CC vs. GG<br>CG vs. GG	1.69 (1.08-2.64)<br>1.61 (1.16-2.24)	0.0646	0.6440	2.15 (1.42-3.24)<br>1.79 (1.33-2.41)	**0.0113**	0.3600	2.41 (1.60-3.62)<br>1.92 (1.31-2.82)	**0.0021**	0.0514
rs1800871 (*IL10*)	TT vs. CC<br>TC vs. CC	1.64 (1.07-2.50)<br>0.99 (0.62-1.58)	0.1740	0.6440	1.66 (1.04-2.64)<br>0.92 (0.55-1.54)	0.1371	0.6694	1.51 (0.91-2.52)<br>0.74 (0.40-1.38)	0.1025	0.5157
rs1800872 (*IL10*)	AA vs. CC<br>AC vs. CC	1.66 (1.10-2.52)<br>1.01 (0.62-1.63)	0.1771	0.6440	1.68 (1.06-2.64)<br>0.93 (0.54-1.59)	0.1433	0.6694	1.52 (0.92-2.52)<br>0.74 (0.39-1.42)	0.1133	0.5157
rs1800896 (*IL10*)	GG vs. AA<br>GA vs. AA	0.77 (0.30-2.00)<br>0.59 (0.33-1.06)	0.2103	0.6440	0.71 (0.24-2.08)<br>0.58 (0.31-1.07)	0.2224	0.7161	0.84 (0.29-2.43)<br>0.54 (0.26-1.10)	0.2146	0.6186
rs2243248 (*IL4*)	GG vs. TT<br>GT vs. TT	0.59 (0.12-2.82)<br>0.99 (0.67-1.46)	0.7299	0.8516	0.51 (0.11-2.39)<br>1.24 (0.77-1.99)	0.4092	0.9269	0.47 (0.09-2.34)<br>1.19 (0.76-1.86)	0.4369	0.7646
rs2243250 (*IL4*)	TT vs. CC<br>TC vs. CC	0.97 (0.56-1.68)<br>0.83 (0.48-1.42)	0.6867	0.8503	1.05 (0.59-1.88)<br>0.86 (0.51-1.44)	0.6875	0.9371	0.97 (0.56-1.67)<br>0.74 (0.45-1.20)	0.3347	0.7501
rs2243270 (*IL4*)	GG vs. AA<br>GA vs. AA	0.90 (0.51-1.60)<br>0.76 (0.45-1.28)	0.4592	0.8503	0.97 (0.54-1.75)<br>0.77 (0.48-1.25)	0.4229	0.9269	0.89 (0.50-1.59)<br>0.67 (0.42-1.06)	0.1699	0.5550
rs1801275 (*IL4R*)	GG vs. AA<br>GA vs. AA	1.43 (0.79-2.60)<br>0.69 (0.41-1.16)	0.0876	0.6440	1.71 (0.78-3.71)<br>0.71 (0.42-1.21)	0.0888	0.6694	1.70 (0.70-4.15)<br>0.69 (0.38-1.25)	0.1204	0.5157
rs1805015 (*IL4R*)	CC vs. TT<br>CT vs. TT	2.86 (1.09-7.48)<br>0.77 (0.49-1.22)	**0.0377**	0.6440	3.47 (0.91-13.23)<br>0.76 (0.47-1.23)	0.0671	0.6694	4.40 (0.87-22.17)<br>0.74 (0.45-1.23)	0.0597	0.4957
rs5918 (*ITGB3*)	CC vs. TT<br>CT vs. TT	2.62 (0.74-9.20)<br>1.00 (0.53-1.90)	0.3390	0.8503	3.17 (0.78-12.95)<br>0.98 (0.51-1.88)	0.2672	0.7544	5.36 (0.92-31.29)<br>0.92 (0.50-1.71)	0.1064	0.5157
rs11003125 (*MBL2*)	GG vs. CC<br>GC vs. CC	0.83 (0.44-1.55)<br>0.84 (0.46-1.57)	0.6573	0.8503	0.91 (0.47-1.75)<br>0.87 (0.46-1.67)	0.7744	0.9531	0.91 (0.43-1.91)<br>0.86 (0.42-1.75)	0.7837	0.9353
rs1800450 (*MBL2*)	AA vs. GG<br>AG vs. GG	0.53 (0.22-1.24)<br>1.30 (0.70-2.42)	0.3483	0.8503	0.45 (0.17-1.19)<br>1.19 (0.61-2.34)	0.4758	0.9269	0.44 (0.16-1.20)<br>1.35 (0.67-2.72)	0.3133	0.7501
rs1800451 (*MBL2*)	AA vs. GG<br>AG vs. GG	 1.16 (0.51-2.65)	0.7186	0.8516	 1.10 (0.41-2.98)	0.8429	0.9633	 1.05 (0.38-2.92)	0.9153	0.9353
rs5030737 (*MBL2*)	TT vs. CC<br>TC vs. CC									
rs7096206 (*MBL2*)	CC vs. GG<br>CG vs. GG	0.99 (0.25-3.97)<br>1.27 (0.84-1.92)	0.5236	0.8503	0.96 (0.21-4.32)<br>1.20 (0.69-2.07)	0.7419	0.9371	0.30 (0.06-1.47)<br>1.22 (0.64-2.34)	0.4091	0.7501
rs1800482 (*NOS2A*)	CC vs. GG<br>CG vs. GG									
rs9282799 (*NOS2A*)	TT vs. CC<br>TC vs. CC									
rs1799983 (*NOS3*)	TT vs. GG<br>TG vs. GG	0.76 (0.21-2.77)<br>0.55 (0.33-0.94)	0.1060	0.6440	0.56 (0.17-1.79)<br>0.51 (0.33-0.78)	**0.0220**	0.3600	0.59 (0.15-2.37)<br>0.52 (0.34-0.80)	0.0540	0.4957
rs2070744 (*NOS3*)	CC vs. TT<br>CT vs. TT	0.47 (0.17-1.27)<br>0.65 (0.46-0.90)	**0.0480**	0.6440	0.38 (0.13-1.14)<br>0.60 (0.45-0.81)	**0.0225**	0.3600	0.40 (0.12-1.39)<br>0.61 (0.48-0.78)	**0.0457**	0.4957
rs662 (*PON1*)	GG vs. AA<br>GA vs. AA	1.16 (0.66-2.03)<br>0.91 (0.63-1.32)	0.5588	0.8503	1.27 (0.75-2.16)<br>1.03 (0.72-1.47)	0.4913	0.9269	1.30 (0.64-2.67)<br>0.94 (0.61-1.45)	0.3972	0.7501
rs854560 (*PON1*)	AA vs. TT<br>AT vs. TT	0.73 (0.25-2.11)<br>0.79 (0.56-1.11)	0.3961	0.8503	0.63 (0.19-2.05)<br>0.71 (0.49-1.03)	0.2387	0.7161	0.83 (0.24-2.90)<br>0.68 (0.42-1.11)	0.3237	0.7501
rs1801282 (*PPARG*)	GG vs. CC<br>GC vs. CC	1.55 (0.46-5.22)<br>0.71 (0.45-1.10)	0.2101	0.6440	1.88 (0.59-6.02)<br>0.72 (0.43-1.19)	0.2053	0.7161	2.24 (0.62-8.11)<br>0.78 (0.49-1.22)	0.2278	0.6201
rs1799762 (*SERPINE1*)	4G4G vs. 5G5G<br>4G5G vs. 5G5G	1.28 (0.68-2.39)<br>0.97 (0.67-1.43)	0.6346	0.8503	1.11 (0.60-2.07)<br>0.86 (0.60-1.23)	0.5931	0.9371	1.02 (0.54-1.92)<br>0.79 (0.53-1.19)	0.4972	0.8401
rs1800468 (*TGFB1/B9D2*)	AA vs. GG<br>AG vs. GG									
rs1800469 (*TGFB1/B9D2*)	TT vs. CC<br>TC vs. CC	0.81 (0.56-1.17)<br>1.07 (0.63-1.83)	0.5256	0.8503	0.83 (0.54-1.29)<br>1.07 (0.59-1.93)	0.6325	0.9371	0.84 (0.46-1.52)<br>1.00 (0.50-2.02)	0.8112	0.9353
rs1800470 (*TGFB1*)	CC vs. TT<br>CT vs. TT	0.96 (0.56-1.63)<br>1.00 (0.64-1.59)	0.9722	0.9722	0.98 (0.54-1.78)<br>1.00 (0.61-1.66)	0.9908	0.9988	1.00 (0.46-2.17)<br>0.90 (0.51-1.62)	0.8880	0.9353
rs4986790 (*TLR4*)	GG vs. AA<br>GA vs. AA									
rs1800629 (*TNF*)	AA vs. GG<br>AG vs. GG	0.30 (0.03-2.60)<br>0.78 (0.42-1.44)	0.4111	0.8503	0.14 (0.01-1.28)<br>0.72 (0.41-1.27)	0.1894	0.7161	0.15 (0.01-1.54)<br>0.70 (0.46-1.06)	0.0719	0.5033
rs1800750 (*TNF*)	AA vs. GG<br>AG vs. GG	 1.52 (0.54-4.27)	0.4062	0.8503	 1.36 (0.46-3.98)	0.5600	0.9269	 1.34 (0.50-3.59)	0.5402	0.8823
rs361525 (*TNF*)	AA vs. GG<br>AG vs. GG	4.01 (0.14-112.31)<br>0.94 (0.41-2.16)	0.6898	0.8503	3.71 (0.31-44.81)<br>0.96 (0.39-2.36)	0.6694	0.9371	3.41 (0.23-49.43)<br>0.90 (0.43-1.89)	0.6252	0.9353
rs2239185 (*VDR*)	CC vs. TT<br>CT vs. TT	0.84 (0.46-1.53)<br>1.28 (0.75-2.20)	0.1531	0.6440	0.85 (0.44-1.65)<br>1.33 (0.77-2.30)	0.1525	0.6694	0.87 (0.41-1.84)<br>1.40 (0.79-2.50)	0.1475	0.5163
rs731236 (*VDR*)	CC vs. TT<br>CT vs. TT	0.82 (0.45-1.50)<br>0.92 (0.56-1.53)	0.7937	0.9044	0.78 (0.43-1.44)<br>0.97 (0.58-1.62)	0.8035	0.9633	0.70 (0.27-1.81)<br>0.88 (0.50-1.56)	0.7228	0.9353
rs890945 (Chr 5q33.3)	AA vs. TT<br>AT vs. TT	1.04 (0.57-1.91)<br>0.85 (0.49-1.45)	0.6643	0.8503	1.27 (0.67-2.40)<br>0.81 (0.44-1.48)	0.4360	0.9269	1.17 (0.54-2.55)<br>0.88 (0.45-1.75)	0.7335	0.9353

CI, confidence interval; FDR, false-discovery rate; OR, odds ratio. Variants with missing results had unstable statistical models.

a) Defined as urinary albumin-to-creatinine ratio (ACR) above ≥ 30 mg/g regardless of gender. b) Analyses adjusted for age, sex, alcohol consumption, educational attainment, and waist:hip ratio.

Table S7. Significant associations of haplotypes in inflammation genes with log(ACR) and albuminuria outcomes

Outcome	Gene	Haplotype	Frequency (%)	Crude Model			Fully Adjusted[b] Model		
				OR or beta (95% CI)	P-value	FDR-adjusted P-value[a]	OR or beta (95% CI)	P-value	FDR-adjusted P-value[a]
Non-Hispanic whites									
**log(ACR)**	*IL4R*	T_A	78.5	Ref			Ref		
		C_A	0.1	0.50 (0.11,0.89)	**0.0136**	0.2108	0.40 (0.08,0.73)	**0.0168**	0.2728
		C_G	16.3	-0.01 (-0.13,0.10)	0.7906	0.9334	0.01 (-0.11,0.13)	0.8683	0.9642
		T_G	5.2	-0.07 (-0.27,0.14)	0.4931	0.9334	-0.01 (-0.25,0.23)	0.9578	0.9642
		OVERALL			0.6453	0.8384		0.8915	0.9164
	MBL2	G_G_C_G_G	28.8	Ref			Ref		
		G_G_T_G_G	6.6	0.11 (-0.17,0.40)	0.4080	0.9334	0.15 (-0.09,0.40)	0.2068	0.9109
		C_G_C_A_G	14.3	0.01 (-0.13,0.15)	0.8936	0.9554	0.00 (-0.13,0.13)	0.9642	0.9642
		C_C_C_G_G	22.4	-0.06 (-0.18,0.06)	0.2954	0.9334	-0.04 (-0.13,0.05)	0.3762	0.9109
		C_G_C_G_G	25.4	-0.16 (-0.26,-0.07)	**0.0014**	**0.0434**	-0.11 (-0.20,-0.02)	**0.0176**	0.2728
		OTHER	2.5	-0.03 (-0.28,0.23)	0.8368		-0.04 (-0.28,0.19)	0.6933	
		OVERALL			0.0871	0.5226		0.1203	0.5166
	NOS2A	C_G	99.9	Ref			Ref		
		OTHER	0.1	0.88 (0.08,1.68)	**0.0332**		0.50 (-0.16,1.15)	0.1288	
		OVERALL			**0.0332**	0.3984		0.1288	0.5166
**Sex-specific ACR[c]**	*MBL2*	G_G_C_G_G	28.8	Ref			Ref		
		G_G_T_G_G	6.6	1.19 (0.62,2.27)	0.5852	0.9426	1.32 (0.69,2.51)	0.3821	0.9590
		C_G_C_A_G	14.3	1.01 (0.75,1.35)	0.9689	0.9689	1.01 (0.77,1.32)	0.9426	0.9647
		C_C_C_G_G	22.4	0.88 (0.62,1.25)	0.4548	0.9426	0.96 (0.67,1.36)	0.7954	0.9590
		C_G_C_G_G	25.4	0.65 (0.47,0.91)	**0.0132**	0.3696	0.67 (0.47,0.97)	**0.0361**	0.9025
		OTHER	2.5	1.26 (0.70,2.26)	0.4253		1.32 (0.78,2.23)	0.2848	
		OVERALL			0.1024	0.4921		0.1309	0.6393
	NOS2A	C_G	99.9	Ref			Ref		
		OTHER	0.1	3.82 (1.12,13.05)	**0.0341**		1.88 (0.57,6.18)	0.2828	
		OVERALL			**0.0341**	0.3751		0.2828	0.6393
	TNF	G_G_G	77.0	Ref			Ref		
		G_A_G	17.1	0.96 (0.65,1.42)	0.8240	0.9689	0.94 (0.58,1.53)	0.8056	0.9590
		OTHER	5.9	1.56 (1.06,2.30)	**0.0246**		1.97 (1.23,3.13)	**0.0064**	
		OVERALL			0.1342	0.4921		0.0647	0.6393
**ACR ≥ 30 mg/g**	*IL10*	G_C_C	46.8	Ref			Ref		
		A_T_A	24.3	1.00 (0.84,1.18)	0.9963	0.9965	1.06 (0.87,1.31)	0.5361	0.9418
		A_C_C	28.8	1.19 (0.87,1.63)	0.2553	0.9965	1.21 (0.89,1.65)	0.2073	0.8336
		OTHER	0.2	5.79 (1.15,29.08)	**0.0341**		1.40 (0.18,11.06)	0.7375	
		OVERALL			0.1821	0.5008		0.4424	0.6341
	MBL2	G_G_C_G_G	28.8	Ref			Ref		
		G_G_T_G_G	6.6	0.92 (0.48,1.77)	0.7894	0.9965	1.03 (0.56,1.89)	0.9164	0.9635
		C_G_C_A_G	14.3	0.95 (0.71,1.27)	0.7242	0.9965	0.94 (0.69,1.28)	0.6781	0.9418
		C_C_C_G_G	22.4	0.78 (0.56,1.10)	0.1494	0.9965	0.83 (0.57,1.22)	0.3374	0.8435
		C_G_C_G_G	25.4	0.61 (0.46,0.82)	**0.0020**	0.0560	0.63 (0.45,0.90)	**0.0122**	0.2650
		OTHER	2.5	1.52 (0.84,2.75)	0.1591		1.57 (0.90,2.73)	0.1072	
		OVERALL			0.0612	0.3366		0.1205	0.3335

Outcome	Gene	Haplotype	Frequency (%)	Crude Model			Fully Adjusted[b] Model		
				OR or beta (95% CI)	P-value	FDR-adjusted P-value[a]	OR or beta (95% CI)	P-value	FDR-adjusted P-value[a]
	PON1	T_G	31.5	Ref			Ref		
		A_A	34.4	0.99 (0.73,1.34)	0.9328	0.9965	0.86 (0.65,1.14)	0.2748	0.8336
		A_G	0.7	0.67 (0.12,3.67)	0.6335	0.9965	0.68 (0.13,3.43)	0.6223	0.9418
		T_A	33.3	0.81 (0.57,1.15)	0.2242	0.9965	0.67 (0.48,0.94)	**0.0212**	0.2650
		OVERALL			0.4607	0.7431		0.0711	0.3335
	TGFB1	G_C_T	53.5	Ref			Ref		
		G_C_C	7.1	0.97 (0.54,1.75)	0.9288	0.9965	0.93 (0.52,1.67)	0.8000	0.9635
		A_C_T	7.7	1.35 (0.86,2.11)	0.1811	0.9965	1.52 (0.92,2.53)	0.1004	0.5020
		G_T_C	31.5	1.25 (0.96,1.62)	0.0911	0.8503	1.30 (0.98,1.72)	0.0655	0.4094
		OTHER	0.2	0.05 (0.00,6.59)	0.2170		0.07 (0.01,0.68)	**0.0241**	
		OVERALL			0.3004	0.6609		0.1334	0.3335
	TNF	G_G_G	77.0	Ref			Ref		
		G_A_G	17.1	0.93 (0.66,1.30)	0.6513	0.9965	0.97 (0.61,1.55)	0.9080	0.9635
		OTHER	5.9	1.67 (1.17,2.39)	**0.0066**		1.92 (1.19,3.10)	**0.0097**	
		OVERALL			**0.0331**	0.3366		0.0721	0.3335
	VDR	C_T	47.4	Ref			Ref		
		C_C	0.5	2.10 (0.30,14.53)	0.4343	0.9965	1.95 (0.25,15.19)	0.5081	0.9418
		T_T	14.3	1.04 (0.82,1.32)	0.7215	0.9965	1.01 (0.78,1.31)	0.9379	0.9635
		T_C	37.7	1.32 (1.05,1.66)	**0.0206**	0.2884	1.26 (1.00,1.59)	0.0537	0.4094
		OVERALL			0.1627	0.5008		0.3226	0.6341
Non-Hispanic blacks									
**log(ACR)**	*CRP*	T_A_C	26.0	Ref			Ref		
		T_C_C	22.8	0.06 (-0.17,0.29)	0.5893	0.9461	0.00 (-0.18,0.18)	0.9935	1.0000
		A_C_C	22.6	-0.14 (-0.28,0.00)	0.0562	0.8130	-0.19 (-0.29,-0.08)	**0.0016**	**0.0480**
		T_T_C	24.2	-0.15 (-0.37,0.08)	0.1999	0.9461	-0.13 (-0.36,0.11)	0.2807	0.7655
		OTHER	4.4	-0.03 (-0.42,0.36)	0.8617		0.00 (-0.39,0.40)	0.9945	
		OVERALL			0.2339	0.8949		0.2441	0.7092
**Sex-specific ACR[c]**	*IL4*	T_C_A	22.3	Ref			Ref		
		G_C_A	6.4	0.99 (0.49,1.98)	0.9714	0.9845	1.18 (0.52,2.70)	0.6746	0.9102
		T_T_A	7.3	1.65 (0.97,2.80)	0.0639	0.4793	2.18 (1.23,3.85)	**0.0094**	0.2820
		G_T_G	7.5	1.03 (0.51,2.06)	0.9322	0.9845	1.12 (0.47,2.69)	0.7911	0.9102
		T_T_G	49.9	1.12 (0.77,1.62)	0.5304	0.9845	1.37 (0.91,2.06)	0.1294	0.5223
		OTHER	6.6	0.92 (0.56,1.51)	0.7269		1.25 (0.68,2.30)	0.4631	
		OVERALL			0.5111	0.8124		0.2606	0.5419
	MBL2	G_G_C_G_G	11.9	Ref			Ref		
		C_G_C_G_G	45.3	0.81 (0.58,1.12)	0.1968	0.6560	0.76 (0.47,1.21)	0.2294	0.6256
		C_C_C_G_G	14.6	0.61 (0.38,0.98)	**0.0405**	0.4050	0.54 (0.30,0.96)	**0.0362**	0.3620
		C_G_C_G_A	23.2	0.95 (0.59,1.51)	0.8097	0.9845	0.91 (0.52,1.59)	0.7211	0.9102
		OTHER	5.0	1.15 (0.51,2.60)	0.7274		1.33 (0.52,3.40)	0.5314	
		OVERALL			0.1606	0.4818		0.1130	0.5166
	TNF	G_G_G	83.3	Ref			Ref		
		G_A_G	12.6	0.67 (0.46,0.96)	**0.0312**	0.4050	0.77 (0.53,1.12)	0.1591	0.5223
		OTHER	4.0	0.59 (0.32,1.10)	0.0942		0.59 (0.27,1.27)	0.1681	
		OVERALL			**0.0354**	0.4248		0.1722	0.5166
	VDR	C_T	40.5	Ref			Ref		
		C_C	2.9	2.05 (1.13,3.72)	**0.0202**	0.4050	2.54 (1.17,5.53)	**0.0208**	0.3120
		T_C	25.8	1.21 (0.90,1.63)	0.1935	0.6560	1.30 (0.96,1.77)	0.0882	0.5223
		T_T	30.7	1.08 (0.93,1.25)	0.3199	0.8725	1.12 (0.95,1.33)	0.1710	0.5223
		OVERALL			0.0816	0.4818		**0.0496**	0.5166

Outcome	Gene	Haplotype	Frequency (%)	Crude Model			Fully Adjusted[b] Model		
				OR or beta (95% CI)	P-value	FDR-adjusted P-value[a]	OR or beta (95% CI)	P-value	FDR-adjusted P-value[a]
**ACR ≥ 30 mg/g**	*CRP*	T_A_C	26.0	Ref			Ref		
		T_C_C	22.8	1.03 (0.61,1.74)	0.8993	0.9374	1.01 (0.61,1.67)	0.9681	0.9681
		A_C_C	22.6	0.74 (0.54,0.99)	**0.0465**	0.6630	0.76 (0.54,1.06)	0.0979	0.7965
		T_T_C	24.2	0.85 (0.58,1.23)	0.3655	0.8958	0.92 (0.58,1.47)	0.7242	0.8753
		OTHER	4.4	0.81 (0.43,1.53)	0.4964		0.93 (0.39,2.23)	0.8622	
		OVERALL			0.4144	0.7517		0.6546	0.8367
	IL4	T_C_A	22.3	Ref			Ref		
		G_C_A	6.4	1.19 (0.62,2.28)	0.5962	0.8958	1.34 (0.61,2.94)	0.4435	0.8572
		T_T_A	7.3	1.61 (0.85,3.05)	0.1392	0.8760	2.24 (1.26,3.98)	**0.0078**	0.2340
		G_T_G	7.5	1.41 (0.68,2.92)	0.3340	0.8958	1.68 (0.70,4.00)	0.2298	0.8447
		T_T_G	49.9	1.14 (0.70,1.83)	0.5870	0.8958	1.38 (0.80,2.40)	0.2348	0.8447
		OTHER	6.6	0.82 (0.40,1.69)	0.5801		1.07 (0.44,2.64)	0.8718	
		OVERALL			0.5675	0.7567		0.3125	0.7514
	TGFB1	G_C_T	51.3	Ref			Ref		
		G_C_C	21.2	1.10 (0.77,1.56)	0.5813	0.8958	1.19 (0.79,1.79)	0.3827	0.8572
		G_T_C	24.5	0.86 (0.59,1.25)	0.4176	0.8958	0.87 (0.55,1.38)	0.5429	0.8572
		OTHER	2.9	0.27 (0.09,0.80)	**0.0205**		0.19 (0.04,0.88)	**0.0347**	
		OVERALL			0.1550	0.6384		0.1750	0.7000
	VDR	C_T	40.5	Ref			Ref		
		C_C	2.9	1.79 (1.08,2.96)	**0.0254**	0.6630	2.24 (1.10,4.56)	**0.0280**	0.4200
		T_C	25.8	1.04 (0.80,1.35)	0.7505	0.9006	1.05 (0.80,1.39)	0.7043	0.8753
		T_T	30.7	0.96 (0.76,1.20)	0.6868	0.8958	0.99 (0.78,1.24)	0.9117	0.9431
		OVERALL			0.1338	0.6384		0.0894	0.7000
Mexican Americans									
**log(ACR)**	*ADRB2*	A_C	40.8	Ref			Ref		
		A_G	0.1	3.68 (1.66,5.71)	**0.0010**	**0.0280**	3.02 (0.67,5.37)	**0.0141**	0.0987
		G_G	21.6	-0.02 (-0.21,0.18)	0.8623	0.9524	0.00 (-0.16,0.17)	0.9740	0.9752
		G_C	37.5	0.02 (-0.16,0.20)	0.8266	0.9524	0.02 (-0.15,0.18)	0.8252	0.9752
		OVERALL			0.0986	0.3354		0.1663	0.5112
	IL10	G_C_C	30.4	Ref			Ref		
		A_T_A	37.5	0.12 (-0.03,0.28)	0.1129	0.3952	0.11 (-0.04,0.27)	0.1509	0.4598
		A_C_C	31.7	0.16 (-0.01,0.32)	0.0662	0.2648	0.19 (0.02,0.36)	**0.0332**	0.1859
		OTHER	0.4	0.98 (0.15,1.82)	**0.0231**		0.83 (0.21,1.44)	**0.0106**	
		OVERALL			0.0594	0.3354		**0.0457**	0.2742
	IL4R	T_A	71.4	Ref			Ref		
		C_A	0.1	-0.64 (-1.07,-0.22)	**0.0047**	**0.0439**	-0.42 (-0.73,-0.11)	**0.0106**	0.0987
		C_G	15.3	0.14 (-0.07,0.35)	0.1802	0.4587	0.20 (-0.06,0.46)	0.1224	0.4284
		T_G	13.2	-0.05 (-0.28,0.18)	0.6653	0.8871	-0.02 (-0.25,0.21)	0.8302	0.9752
		OVERALL			0.2955	0.5910		0.2063	0.5112
	NOS3	T_G	69.9	Ref			Ref		
		T_T	5.8	0.16 (-0.16,0.49)	0.3081	0.6636	0.16 (-0.14,0.46)	0.2911	0.6664
		C_G	10.7	0.15 (-0.07,0.38)	0.1745	0.4587	0.15 (-0.07,0.37)	0.1642	0.4598
		C_T	13.6	-0.23 (-0.45,-0.00)	**0.0456**	0.2565	-0.30 (-0.50,-0.11)	**0.0042**	0.0987
		OVERALL			0.0730	0.3354		**0.0134**	0.1608

Outcome	Gene	Haplotype	Frequency (%)	Crude Model			Fully Adjusted[b] Model		
				OR or beta (95% CI)	P-value	FDR-adjusted P-value[a]	OR or beta (95% CI)	P-value	FDR-adjusted P-value[a]
	PON1	T_G	45.5	Ref			Ref		
		A_A	21.7	0.02 (-0.26,0.30)	0.8960	0.9524	-0.02 (-0.26,0.22)	0.8595	0.9752
		A_G	1.0	-0.20 (-0.67,0.28)	0.3979	0.7958	-0.30 (-0.66,0.05)	0.0876	0.3504
		T_A	31.8	0.12 (0.00,0.25)	**0.0458**	0.2565	0.09 (-0.01,0.19)	0.0814	0.3504
		OVERALL			0.3648	0.6254		0.3219	0.6438
	VDR	C_T	57.8	Ref			Ref		
		C_C	0.8	0.10 (-0.49,0.68)	0.7354	0.8969	-0.01 (-0.71,0.68)	0.9752	0.9752
		T_T	18.5	0.18 (0.07,0.28)	**0.0020**	**0.0280**	0.16 (0.05,0.27)	**0.0072**	0.0987
		T_C	22.9	0.04 (-0.12,0.20)	0.6270	0.8871	0.05 (-0.13,0.22)	0.5747	0.8649
		OVERALL			0.1118	0.3354		0.2130	0.5112
**Sex-specific ACR[c]**	*CRP*	A_C_C	36.3	Ref			Ref		
		T_T_A	11.4	0.87 (0.46,1.64)	0.6474	0.7936	0.83 (0.49,1.40)	0.4667	0.7821
		T_C_C	21.4	1.02 (0.73,1.44)	0.8858	0.9182	1.08 (0.72,1.63)	0.7035	0.7821
		T_T_C	24.5	1.16 (0.87,1.56)	0.3014	0.7192	1.21 (0.88,1.66)	0.2329	0.6405
		OTHER	6.4	0.39 (0.19,0.83)	**0.0164**		0.41 (0.21,0.81)	**0.0120**	
		OVERALL			0.1876	0.4964		0.1251	0.3753
	IL10	G_C_C	30.4	Ref			Ref		
		A_T_A	37.5	1.53 (1.04,2.24)	**0.0321**	0.2354	1.47 (0.93,2.33)	0.0979	0.5643
		A_C_C	31.7	1.40 (0.88,2.23)	0.1448	0.6371	1.48 (0.85,2.55)	0.1539	0.5643
		OTHER	0.4	3.29 (0.62,17.54)	0.1539		2.83 (0.78,10.23)	0.1074	
		OVERALL			0.0901	0.4505		0.1711	0.3850
	NOS3	T_G	69.9	Ref			Ref		
		T_T	5.8	1.43 (0.72,2.84)	0.2860	0.7192	1.58 (0.75,3.36)	0.2193	0.6405
		C_G	10.7	1.11 (0.70,1.76)	0.6452	0.7936	1.11 (0.69,1.79)	0.6387	0.7821
		C_T	13.6	0.52 (0.32,0.85)	**0.0121**	0.2354	0.40 (0.25,0.66)	**0.0010**	**0.0220**
		OVERALL			0.0884	0.4505		**0.0290**	0.2403
	TNF	G_G_G	87.2	Ref			Ref		
		G_A_G	6.7	0.57 (0.35,0.93)	**0.0267**	0.2354	0.53 (0.33,0.86)	**0.0122**	0.1342
		OTHER	6.1	0.87 (0.40,1.90)	0.7198		0.72 (0.40,1.32)	0.2756	
		OVERALL			0.2590	0.4964		0.0534	0.2403
**ACR ≥ 30 mg/g**	*ADRB2* [d]	A_C	40.8	Ref					
		A_G	0.1	18.73 (1.08,324.33)	**0.0445**	0.4583			
		G_G	21.6	1.04 (0.70,1.55)	0.8375	0.9547			
		G_C	37.5	1.01 (0.74,1.38)	0.9547	0.9547			
		OVERALL			0.3452	0.5970			
	NOS3	T_G	69.9	Ref			Ref		
		T_T	5.8	1.16 (0.54,2.50)	0.6927	0.9547	1.31 (0.58,2.99)	0.5022	0.8266
		C_G	10.7	0.96 (0.63,1.45)	0.8231	0.9547	1.03 (0.66,1.61)	0.8981	0.8981
		C_T	13.6	0.44 (0.25,0.76)	**0.0048**	0.1200	0.36 (0.23,0.55)	**<0.0001**	**<0.0001**
		OVERALL			0.0781	0.5970		**0.0300**	0.2700
	TNF	G_G_G	87.2	Ref			Ref		
		G_A_G	6.7	0.69 (0.42,1.13)	0.1294	0.8088	0.62 (0.39,1.00)	**0.0489**	0.5379
		OTHER	6.1	1.01 (0.48,2.13)	0.9771		0.95 (0.46,1.97)	0.8816	
		OVERALL			0.4776	0.5970		0.3138	0.5762

ACR, albumin-to-creatinine ratio; CI, confidence interval; FDR, false-discovery rate; OR, odds ratio.

a) FDR-adjusted P-values not calculated for the "other" haplotype group. b) All analyses adjusted for age, alcohol consumption, educational attainment, and waist:hip ratio. Analyses of log(ACR) and ACR ≥ 30 mg/g also adjusted for sex. c) Defined as urinary albumin-to-creatinine ratio (ACR) ≥ 17 mg/g in men and ≥ 25 mg/g in women. d) Adjusted model errored and is not presented.
